# Transposable elements and heterochromatic regions are enriched for structural variation and sequence divergence in the genome of wild-type *Caenorhabditis elegans*

**DOI:** 10.1093/g3journal/jkaf092

**Published:** 2025-04-30

**Authors:** Zachary D Bush, Alice F S Naftaly, Devin Dinwiddie, Cora Albers, Kenneth J Hillers, Diana E Libuda

**Affiliations:** Institute of Molecular Biology, Department of Biology, University of Oregon, Eugene, OR 97403, USA; Institute of Molecular Biology, Department of Biology, University of Oregon, Eugene, OR 97403, USA; Institute of Molecular Biology, Department of Biology, University of Oregon, Eugene, OR 97403, USA; Institute of Molecular Biology, Department of Biology, University of Oregon, Eugene, OR 97403, USA; Biological Sciences Department, California Polytechnic State University, San Luis Obispo, CA 93407, USA; Institute of Molecular Biology, Department of Biology, University of Oregon, Eugene, OR 97403, USA

**Keywords:** genome stability, sequence variation, reference genomes, genetic drift, transposons, whole-genome sequencing, *C. elegans*, genome assembly, WormBase

## Abstract

Genomic structural variants (SVs) and transposable elements (TEs) can be significant contributors to genome evolution, gene expression alterations, and genetic disease risk. Recent advancements in long-read sequencing have greatly improved the quality of *de novo* genome assemblies and enhanced the detection of larger and highly repetitive sequence variants at the scale of hundreds or thousands of bases. Comparisons between 2 diverged wild isolates of *Caenorhabditis elegans*, the Bristol and Hawaiian strains, have been widely utilized in the analysis of small genetic variations. To comprehensively detect SVs and TEs, we generated *de novo* genome assemblies and annotations for the N2 Bristol and CB4856 Hawaiian *C. elegans* strains from our lab collection using both long- and short-read sequencing. Within our lab assemblies, we annotate over 3.1 Mb of sequence divergence between the Bristol and Hawaiian isolates: 246,298 homozygous single-nucleotide polymorphisms (SNPs), 73,789 homozygous small insertion-deletions (<50 bp), and 4,334 SVs (>50 bp). We also define the location and movement of specific TEs between N2 Bristol and CB4856 Hawaiian wild-type isolates. Specifically, we find the N2 Bristol genome has 20.6% more TEs from the *Tc1/mariner* family than the CB4856 Hawaiian genome. Moreover, we identified Zator elements as the most abundant and mobile TE family in the genome. Using specific TE sequences with unique SNPs, we also identified 9 TEs that moved intrachromosomally and 8 TEs that moved to new chromosomes between the N2 Bristol and CB4856 Hawaiian genomes. Further, we show an enrichment of genomic variation in transposon sequences and silenced heterochromatic regions of chromosomes in the germline. Taken together, our studies demonstrate how specific regions of the genome, including large-scale repetitive regions, are more susceptible to accumulation of genetic variation and changes to genome structure.

## Introduction

The rise of genomic variation between individuals and genetic drift between populations underly a core process of evolution. Functional characterization of sequence variants can guide our understanding of phenotypic variances within species while also being critical to identifying heritable disease-causing mutations (Haraksingh and Snyder 2013). Genomic variation occurs at multiple scales, from single-nucleotide polymorphisms (SNPs) to short insertions/deletions (indels) to much larger structural variants (SVs). SVs are defined as insertions, deletions, or chromosomal rearrangements that are a minimum of 50 bp in length. SVs can cause loss-of-function mutations through large gene deletions or alter gene expression by disrupting spatial interactions between regulatory sequences (Stranger *et al*. 2007; Hurles *et al*. 2008). Accurate detection of both sequence variants and chromosome rearrangements is critical for understanding how genomic variation may contribute to phenotypic variation in individuals and populations of the same species.

Transposable elements (TEs) are a class of repetitive DNA sequences capable of moving to new locations in the genome. TE mobility is another source of genomic structural variation that can also alter gene expression (Girard and Freeling 1999; Slotkin and Martienssen 2007) and can drive rapid evolutionary changes within species (Feschotte and Pritham 2007; Van’t Hof *et al*. 2016). Notably, transposons or TE remnants account for a significant fraction of the total DNA sequence in many eukaryotic species (Chalopin *et al*. 2015; Gilbert *et al*. 2021), which provides many opportunities for TE-driven structural rearrangements. The *Tc1/mariner* family of DNA transposons is 1 of the most abundant TEs across species (Eide and Anderson 1985; Plasterk *et al*. 1999), and early studies in *Caenorhabditis elegans* found it to be 1 of the few mobile transposons observed under laboratory conditions (Fischer *et al*. 2003). Transposon mobility and repression are tightly regulated through multiple mechanisms including chromatin modification and RNA interference (Sijen and Plasterk 2003; Lee *et al*. 2012), and naturally acquired mutations in the transposase coding sequence can also disrupt their mobility (Lohe *et al*. 1997). Despite their ubiquity and impact on genomic architecture, the comprehensive detection, annotation, and inclusion of TEs in comparative genomic analyses remains challenging due to their highly repetitive sequence nature. Many studies have incompletely characterized the genomic distribution of TEs because older, short-read-based methods could not accurately map the full content and location of repetitive sequences (Goerner-Potvin and Bourque 2018). Further, programs that automatically detect TEs based on sequence homology and conserved sequence elements rely heavily on libraries of older reference sequences that may predate the discovery of TE fragments and newer TE families. As new families of TEs are discovered (Bao *et al*. 2009), along with new technology that aids their annotation and tracking (Riehl *et al*. 2022), determining the genomic composition and mobility of new TEs will enable our understanding of their role in the genome evolution and genome integrity.

Foundational research on genomic variation utilized next-generation short-read sequencing, long-read sequencing, and the direct comparison of reference genome assemblies to identify genomic variants (Lappalainen *et al*. 2019; Mahmoud *et al*. 2019). SNPs and indels, ranging in size from 1 to 50 bp, can be identified with high confidence using short sequencing reads that are 100–150 bp at high read depths (Muzzey *et al*. 2015). In contrast, SVs are challenging to annotate using short-read sequencing because the sequencing reads are often smaller than the size of an SV (Sudmant *et al*. 2015; Mahmoud *et al*. 2019; Lesack *et al*. 2022). Similarly, the highly repetitive sequences of TEs present significant challenges to mapping and annotation with traditional short-read sequencing methods. With the advent of higher quality long-read sequencing technologies, which generate ∼10–30 kb reads with lower genomic coverage, the accurate annotation of large regions of genomic variation such as SVs and TEs has become easier (Sakamoto *et al*. 2021). Methods of genome assembly that leverage the strengths of both short- and long-read sequencing can provide more accurate reference sequences to fully address undiscovered genomic variations previously not detected by short-read sequencing alone. Notably, some new tools to identify SVs via assembly-to-assembly alignments (Delcher *et al*. 1999; Nattestad and Schatz 2016; Li 2018; Goel *et al*. 2019) are not constrained by read-length to identify SVs and depend on high-quality reference assemblies. Overall, a high-quality reference genome assembly using multiple sequencing platforms and tools can generate a more comprehensive, accurate genome that serves as a critical resource for genomic and genetic studies in any model organism.

Not all regions of the genome accumulate sequence variants at the same rate. For instance, in non-coding DNA regions, base substitutions and short indels are frequently less deleterious than changes to exons (Ellegren *et al*. 2003). Moreover, tandemly repeating sequences have enhanced rates of variation (Zhang *et al*. 2022), suggesting that local sequence context may be a major determinant for mutation accumulation. Further, there is increasing evidence that the structural organization of DNA, in the context of different chromatin states, plays a role in shaping the landscape of genetic variation in multiple species (Makova and Hardison 2015). Importantly, while regional differences in the accumulation rates of SNPs and indels have been studied, how structural variations and rearrangements contribute to variable mutation rates is not fully known. The development of methods that can identify both structural variations and larger regions of exceptional sequence divergence provides new opportunities to study how different regions of the genome accumulate different types and sizes of variants.

*Caenorhabditis elegans* was the first multicellular organism to have its genome fully sequenced (*C. elegans* Sequencing Consortium 1998) and has been exploited to pioneer many comparative genomic studies. To understand how genetic variation influences phenotypic differences and genomic processes within species, *C. elegans* researchers primarily utilize 2 highly diverged wild-type strains estimated to have diverged 30,000–50,000 generations ago (Thomas *et al*. 2015): N2 (isolated in Bristol, England) and CB4856 (isolated in Maui, Hawaii) (Nicholas *et al*. 1959; Sulston and Brenner 1974; Hodgkin and Doniach 1997; Crombie *et al*. 2019). Earlier comparisons of the Bristol and Hawaiian lineages were critical for studying genetic variation, gene families, and evolution of genome structures (Koch *et al*. 2000; Wicks *et al*. 2001; Stewart *et al*. 2005; Maydan *et al*. 2010). The *C. elegans* genome, comprised of 5 autosomes and the *X* chromosome, displays a nonuniform distribution of sequence variation when comparing the genomes of wild isolates. Although a large amount of sequence divergence was previously found between the N2 Bristol and CB4856 Hawaiian lineages (Andersen *et al*. 2012; Thompson *et al*. 2015), the increased quality of reference genomes, sequencing technology, and variant detection methods renews the need for comprehensive identification of variations (in particular large structural variations) that previously went undetected in these *C. elegans* genomes.

The first CB4856 Hawaiian genome assembly was completed in 2015 by iteratively correcting the preexisting N2 Bristol reference assembly (*C. elegans* Sequencing Consortium 1998) with short-read sequencing data (Thompson *et al*. 2015). This study identified 327,050 SNPs and nearly 80,000 indels relative to N2; a marked increase relative to previous comparisons, which had identified 6,000–17,000 SNPs and small indels (Wicks *et al*. 2001; Swan *et al*. 2002) between N2 Bristol and CB4856 Hawaiian. Due to the size of the short-read sequences employed in the analysis, the iterative correction method used to assemble the CB4856 Hawaiian genome may not have detected all structural rearrangements and repetitive sequences. In 2019, the first *de novo*  CB4856 Hawaiian assembly from PacBio long-read sequencing extended the length of the Hawaiian genome by ∼5.6 Mb. By comparing this CB4856 Hawaiian assembly to the original N2 assembly, this study was able to characterize over 3,000 SVs (Kim *et al*. 2019). Thus, combining long-read and short-read sequencing in *de novo* genome assembly not only extended the known length of the CB4856 Hawaiian genome, but broadened our understanding of how much genomic variation exists between these wild-type strains.

TEs are highly abundant in the *C. elegans* genome and are likely a source of genomic SVs due to their ability to autonomously excise and subsequently insert themselves into new locations. Early TE analyses of the *C. elegans* genome indicated that ∼12–16% of the genome is comprised of TEs (*C. elegans* Sequencing Consortium 1998; Bessereau 2006) and *Tc1/mariner* elements can be active in laboratory strains (Emmons *et al*. 1983; Liao *et al*. 1983). The Zator superfamily, a recently characterized TE, encodes a transposase that is distantly similar to those in the *Tc1/mariner* superfamily which evolved from bacterial *IS630* transposons. Further, phylogenetic analyses suggest that Zator elements can be considered as a distinct family of eukaryotic TEs evolved from a bacterial *TP36*-like transposon (Bao *et al*. 2009). To our knowledge, the movement of Zator elements and other recently identified TE families has not yet been analyzed in *C. elegans* laboratory strains. While TE distributions have been assessed in wild *C. elegans* strains using older reference genomes and Illumina short-read sequencing (Laricchia *et al*. 2017), the complete TE composition has not yet been reassessed in assemblies built from long-read sequencing nor since the identification of new families of eukaryotic class II transposons (Bao *et al*. 2009).

To more accurately determine the extent of sequence and structural variation between N2 Bristol and CB4856 Hawaiian genomes, we generated 2 high-quality, long-read reference assemblies with the same assembly pipelines for both the N2 and CB4856 strains used in our laboratory. By leveraging recent technological advancements in sequencing and variant detection, we provide a comprehensive annotation of SNPs, indels, structural variations, and TEs between our lineages of the Bristol and Hawaiian strains. From our comprehensive mapping of TEs in our reference genomes, we report Zator elements to be the most abundant and mobile TE family in the *C. elegans* genome. Further, we find that variations are depleted from gene regulatory sequences such as promoters and enhancers. Notably, we find that TE sequences and heterochromatin regions harbor an enrichment of all types of variants detailed in this study, particularly for SVs and highly diverged regions. Taken together, our systematic and comprehensive analysis of genetic variation in these 2 wild-type isolates reveals how different genomic regions and particular TEs may uniquely contribute to genetic drift and the evolution of genome structure.

## Methods

### *Caenorhabditis elegans* culture and sucrose floatation

The N2 Bristol and CB4856 Hawaiian strains of *C. elegans* were grown at 20°C on standard NGM agar plates seeded with the OP50 strain of *Escherichia coli* as a food source. To minimize bacterial contamination in downstream genomic DNA (gDNA) sample preps, we performed sucrose floatation on pooled populations of each isolate. Worms were washed from plates with 8 mL cold M9 buffer and transferred to 15-mL glass centrifuge tubes using a glass Pasteur pipette. Collected worms were centrifuged at 3,000 rpm at 4°C and washed in 4 mL of fresh M9 twice. To separate worms from bacteria and other debris, 4 mL of 60% sucrose solution was added to 4 mL of M9 buffer and worms and vortexed briefly. The mixture was then spun at 5,000 rpm at 4°C for 5 min. Using a glass pipette, the floating layer of worms was transferred to a new glass centrifuge tube on ice and brought up to 4 mL in fresh M9. Worms were then incubated at room temp for 30 min and gently vortexed every 5 min. Worms were washed 3 times in equal volumes of fresh M9 before storing collected worms in M9 at 20°C before gDNA extraction.

### Long-read and short-read sequencing

Genomic DNA was extracted from worms using the Qiagen DNeasy Blood and Tissue Kit. Sequencing was performed on pooled populations of N2 and CB4856 after reducing bacterial contamination by sucrose float for each strain. For PacBio HiFi long-read sequencing, library preparation was performed on pooled populations of worms for each isolate by the University of Oregon's Genomics and Cell Characterization Core Facility and sequenced on the Sequel II system. Raw PacBio HiFi subreads were then refined into circular consensus sequencing (CCS) and assessed using FASTQC. CCS reads were then used for all further applications. For Illumina short-read sequencing, library preparation was performed on pooled populations of worms for each isolate by the University of Oregon's Genomics and Cell Characterization Core Facility. The short-read libraries were then sequenced on an Illumina HiSeq4000 (2 × 150 bp).

### Long-read genome assembly and short-read refinement

PacBio HiFi CCS long-reads were aligned to the *E. coli* genome using BWA (Li and Durbin 2009) (version 0.7.17), and reads that aligned to the bacterial genome were removed. Following our sucrose floatation protocol, we found that <0.2% of reads aligned to the bacterial genome (0.1% for CB4856 and 0.02% for N2). *De novo* genome assembly was performed for N2 Bristol and CB4856 Hawaiian using Canu (Koren *et al*. 2017) (version 1.7). For each isolate, we generated 3 independent sets of contigs from Canu. For both genomes, all Canu runs produced identical contig sets and only one set was used for downstream applications (Supplementary Fig. 5). To refine the long-read assemblies, short-reads from each isolate were aligned to their respective long-read assembly using BWA-MEM (version 0.7.17). Aligned reads in SAM format were sorted and converted to BAM format using SAMtools (Li *et al*. 2009). Using Picard (https://broadinstitute.github.io/picard/), read groups were added via AddOrReplaceReadGroups, and duplicate reads were filtered using MarkDuplicates. Some bases may have been inaccurately called due to lower sequencing coverage, larger error rate in PacBio sequencing, or predominating alleles present in the population of each isolate that could be revealed by greater sequencing depth afforded by Illumina sequencing. GATK's HaplotypeCaller (McKenna *et al*. 2010) and FreeBayes (Garrison and Marth 2012) were utilized to generate VCF files representing potentially inaccurate sites in each initial assembly. Coverage thresholds were manually determined using the Integrative Genomics Viewer (IGV) for each assembly (Robinson *et al*. 2011). Sites were filtered according to manual values using VCFtools (Danecek *et al*. 2011; Danecek and McCarthy 2017). Error correction was performed on single-nucleotide alleles using BCFtools *consensus* (Danecek and McCarthy 2017) and alternate indel alleles. After filtering potential sites by sequencing depth thresholds determined for each chromosome, this left 4,237 and 36,145 corrections for the N2 Bristol and CB4856 Hawaiian genomes, respectively. Of these sites, <0.7% were unable to be resolved, and all of these were short indels comprising <0.001% of each genome.

### Assessing genome assembly completeness

Each genome assembly was evaluated using QUAST-LG with PacBio and Illumina reads provided for read analysis (Supplementary Data Files 11 and 12; Gurevich *et al*. 2013; Mikheenko *et al*. 2018). To determine whether any assembly artifacts remained after polishing, sequencing reads from each genome were then re-aligned to their respective, completed genome assembly. No significant losses in read alignment or coverage were observed. To further assess the quality and completeness of our N2 Bristol and CB4856 Hawaiian assemblies, we used BUSCO (Simão *et al*. 2015; Manni *et al*. 2021). BUSCO was run in a Docker container (https://busco.ezlab.org/busco_userguide.html) in genome mode. For each assembly, the quality and presence of expected orthologous genes was checked against the nematoda and metazoan lineage databases.

### SNP and indel calling in N2 and CB4856 assemblies

Illumina short-reads from the N2 Bristol and CB4856 Hawaiian genome were trimmed using Trimmomatic (Bolger *et al*. 2014) to remove adapter and barcode sequences. The trimmed CB4856 reads were then aligned to the N2 Bristol reference genome using BWA-MEM so that SNPs and indels present between N2 Bristol and CB4856 Hawaiian could be identified. All resulting variant positions comparing our N2 Bristol and CB4856 Hawaiian genomes are in relation to the N2 Bristol assembly. Aligned reads in SAM format were then sorted using SAMtools (Li *et al*. 2009) and converted to BAM files. Using Picard read groups were added via AddOrReplaceReadGroups, and duplicate reads were filtered using MarkDuplicates as described above. BAM files with filtered duplicate reads were used to call variants using a combination of GATK HaplotypeCaller, FreeBayes, and BCFtools. The 3 resulting VCF files containing SNPs and indels were then concatenated, further filtered for duplicate sites and low-quality variants, and sorted using BCFtools. SNPs with QUAL scores of 30 or greater, a minimum of 10 variant reads, and a minimum of 30 total, high-quality reads were retained.

### Calling structural variants using whole-genome alignments

All assembly-to-assembly alignments were performed using Minimap2 (Li 2018). SyRI (Goel *et al*. 2019) was then used to parse the resulting SAM files and call SVs and highly divergent regions [HDRs (Structural rearrangements were plotted with the aid of Plotsr within the SyRI package)]. “NOTAL” or non-alignable regions in each genome were retained as SVs. To acquire NOTAL regions in each query genome, the Minimap2 alignment was repeated with the original reference, and query genomes swapped. The sizes of HDRs depicted in Table 1 are sizes relative to the reference genome in each comparison (i.e. N2 Bristol in Table 1). HDRs called by SyRI represent regions of pairwise alignment between reference genome assemblies where there are multiple gaps present at the same corresponding locus in each genome. These could be due to the presence of multiple SNPs, indels, or SVs of varying complexity that each genome acquired independently at the same site. These are distinct from “hyper divergent regions” as seen in prior studies (Thompson *et al*. 2015; Lee *et al*. 2021) that use different approaches (e.g. sliding window analyses) to measure the local density of variants in kilobase-scale regions. All SV and HDR calls from SyRI are available in Supplementary Files 3 and 4. Example IGV visualizations for SV calls and HDRs are included in Supplementary Fig. 4.

**Table 1.** Comparisons between the N2 Bristol genome and CB4856 Hawaiian genome.

	Chromosomes						Total
	*I*	*II*	*III*	*IV*	*V*	*X*	
N2 Bristol chromosome length	15,114,068	15,311,845	13,819,453	17,493,838	20,953,657	17,739,129	100,431,990
CB4856 Hawaiian chromosome length	15,045,644	15,257,363	13,206,755	17,183,882	20,547,529	17,584,915	98,826,088
N2 Bristol bases aligned	15,100,574	15,303,320	13,222,676	17,330,119	20,947,147	17,738,394	99,642,230 (99.21%)
% Syntenic aligned bases	93.31	88.56	90.61	95.42	87.04	98.73	92.23
SNPs*^a^*	30,394	48,365	29,881	30,497	87,300	19,861	246,298
Indels*^a^*	11,460	13,716	10,530	11,221	20,063	6,799	73,789 (275,442 bp)
SVs	863	808	649	619	925	470	4,334 (2,654,902 bp)
HDRs	185	270	165	138	356	60	1,174 (6,864,884 bp)

^a^All variants listed are only those for which the CB4856 Hawaiian genome was homozygous.

### Converting gene annotations between assemblies

We converted gene annotations from the N2 reference assembly (cel235) to our N2 Bristol and CB4856 Hawaiian assemblies. The gene annotations for the WBcel235 genome assembly were downloaded in GFF3 format from Ensembl (http://ftp.ensembl.org/pub/release-105/gff3/caenorhabditis_elegans/). Unlike previously established tools that require pre-generated chain files (James *et al*. 2003), Liftoff (Shumate and Salzberg 2021) can accurately remap gene annotations onto newly generated assemblies using Minimap2 assembly-to-assembly alignments. Rather than aligning whole genomes, Liftoff aligns only regions listed in the annotation files so that genes may be remapped even if there are large structural variations between 2 genomes. The Liftoff program was then used to remap annotations between the WBcel235 assembly onto each new genome assembly for N2 Bristol and CB4856 Hawaiian (Supplementary Data Files 5 and 6).

### Testing the association of variant sites in gene annotations and chromatin profiles

For each chromosome, to determine whether SNPs or indels were enriched within gene annotations, fold enrichment analyses were performed using the genomic association tester (GAT) (Heger *et al*. 2013) tool (https://github.com/AndreasHeger/gat.git). The observed enrichment of each variant type in gene annotations was compared with overlaps in simulated distributions SNPs or indels. First, the genomic intervals available for simulated distributions were restricted to mutually exclusive “workspaces” for the central domain of each chromosome and the combined arm-like regions. Thus, all of the following overlaps, enrichment values, and *P*-values were computed separately for variants, annotations, and chromatin profiles that reside within the central vs arm-like domains. Simulated distributions were created using 20,000 iterations whereby each variant type was randomly and uniformly distributed across each workspace. All variant distributions were compared against intergenic, gene, intron, exon, UTR, transcription factor (TF)-binding sites, promoter, enhancer, and TE annotations. Comparing the observed enrichment to the simulated distributions, statistical significance was assigned to the observed fold enrichment with *P*-values calculated from a hypergeometric test calculated within GAT. Per-chromosome BED files for SNP intervals were created from their original VCF using AWK. Per-chromosome BED files for indel intervals were calculated using a custom script. The GFF3 formatted annotations generated via liftoff were then broken down by chromosome, gene, exon, and UTR regions. Because intron regions were not explicitly written into each GFF3 file, they were calculated using BEDtools (Quinlan and Hall 2010). First, a joint BED file containing the UTR and exon regions was made using AWK and sorted first by chromosome and then by position. Using BEDtools these intervals were combined, and intronic regions were calculated by finding regions in gene intervals not covered by either UTR or exons. Intergenic spaces on each chromosome were calculated with the gene BED files and chromosome sizes as inputs. Germline ChIP-seq (NCBI BioProject PRJNA475794) data were aligned to the N2 genome and peaks were called in MACS3 (Feng *et al*. 2012) using parameters as previously described (Tabuchi *et al*. 2018; Supplementary Data Files 7–9). Germline-specific genomics datasets were chosen as mutations and variations that arise in the germline are more likely to persist through development and fix in larger populations (Yu *et al*. 2024).

### TE identification and tracking

The TransposonUltimate pipeline was run for both our N2 Bristol and CB4856 Hawaiian genome assemblies (Supplementary Data File 10). MUST and SINE finder were run independently and integrated into the filtering steps of the pipeline manually. Additionally, we added LTR retriever to the TE identification ensemble to supplement the LTR harvest and LTR finder. To identify uniquely identifiable copies of TEs that can be tracked for evidence of transposition, we first identified TE sequences that overlapped with SNPs using BEDtools. To ensure that a polymorphic TE sequence is retained in both the CB4856 Hawaiian and N2 genome, the variant TE bases in CB4856 Hawaiian TEs were applied to each corresponding N2 Bristol TE sequence, and these sequences were cross-referenced with the original TransposonUltimate output for CB4856 Hawaiian for matches. Unique polymorphic TE sequences found in both genomes were then assessed for translocation events by examining genomic start coordinates in each genome. Utilizing whole-genome alignments for each chromosome, TEs were first predicted to have moved if the starting coordinates for each TE pair did not correspond to relative changes in coordinates due to alignments. We then filtered our transposition dataset based on visualization of long- and short-read alignments in IGV and required that the 50-bp sequences flanking each TE that moved be no more similar than 90% based on local pairwise sequence alignments. Alignment scoring parameters were chosen to match those settings in Minimap2's “asm5” alignment parameters for aligning genomes of relatively low divergence.

## Results

### *De novo* genome assembly using combined long- and short-read sequencing

**Fig. 1.**
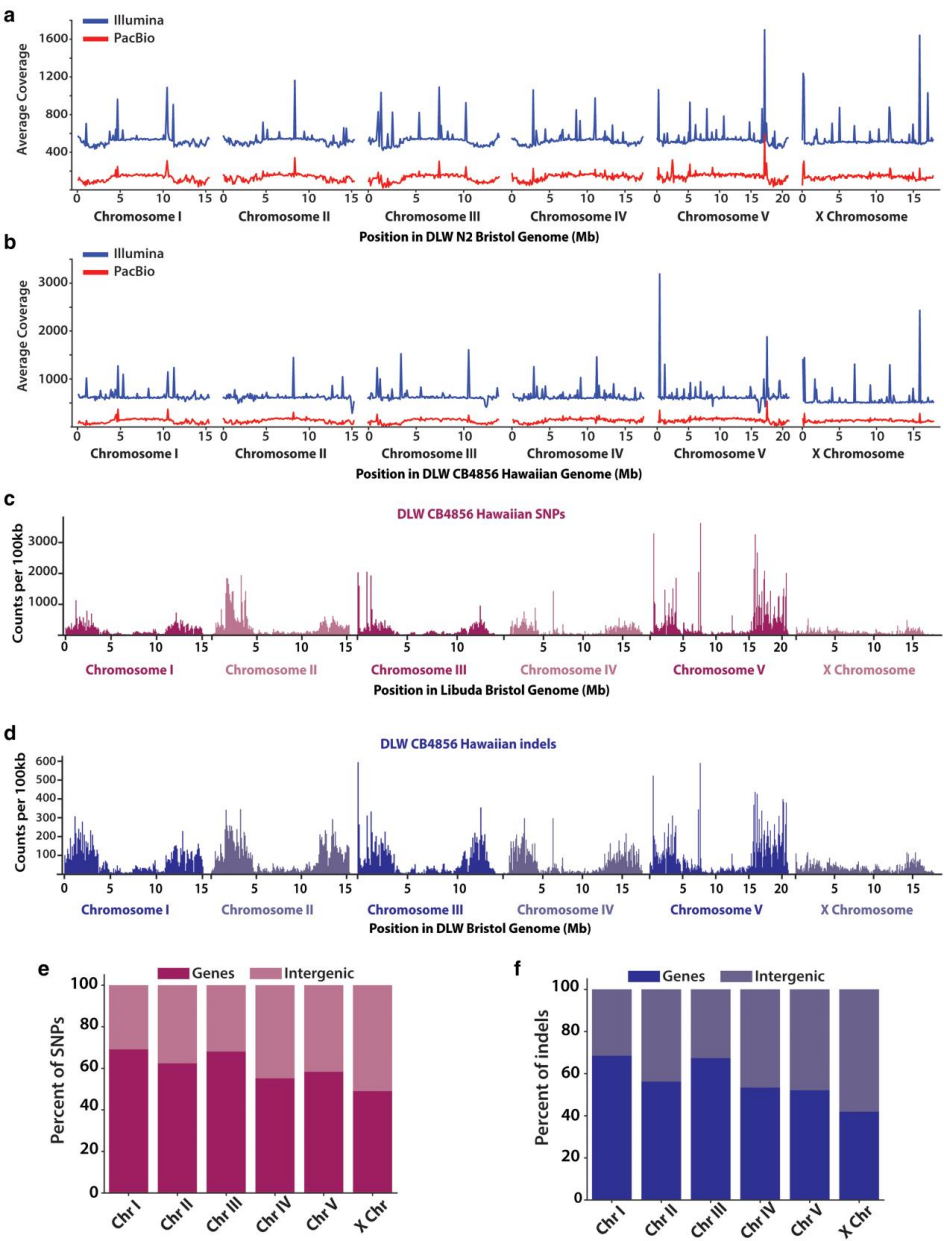
Genomic distribution of SNPs and indels between the N2 Bristol and CB4856 Hawaiian genomes. a) Line plots showing the average sequencing coverage in 100-kb bins across each chromosome in the N2 Bristol genome. b) Line plots showing the average sequencing coverage in 100 kb bins across each chromosome in the CB4856 Hawaiian genome. For each plot in a and b), the coverage for Illumina short-read sequencing is shown in blue, and sequencing coverage for PacBio long-reads is shown in red. For both a and b), sequencing reads from the genome of each isolate were re-aligned to the completed genome assemblies, respectively. c) Histograms depicting the distribution of CB4856 Hawaiian SNPs across each N2 Bristol chromosome in 100 kb bins. d) Histograms of the distributions of CB4856 Hawaiian indels across each N2 Bristol chromosome in 100 kb bins. e) The proportion of SNPs that overlap with remapped gene annotations vs intergenic regions in the N2 Bristol genome. f) The proportion of indels that overlap with gene vs intergenic regions in the Bristol genome.

To perform systematic comparisons of different wild-type laboratory isogenic strains, we generated *de novo* assemblies of N2 Bristol and CB4856 Hawaiian. The N2 Bristol genome was assembled from PacBio HiFi long-reads with 136× coverage producing 121 contigs and a 100.4 Mb genome (Fig. 1a). The CB4856 Hawaiian genome was generated from PacBio HiFi long-reads with 132× coverage from 169 contigs to give a 98.8 Mb assembly (Fig. 1b). These long-read assemblies were then supplemented with Illumina paired-end short-reads with a sequencing depth of 540× and 628× for N2 Bristol and CB4856 Hawaiian, respectively (Fig. 1a and b).

To assess the quality of our reference genomes, we examined assembly-to-assembly alignments between our N2 Bristol and Hawaiian genomes and quantified the orthologous gene content for each assembly. Since the genomes of these 2 isolates are highly homologous, whole-genome alignments should show a high proportion of aligned bases synteny when comparing our N2 Bristol to CB4856 Hawaiian assemblies. Indeed, 99.2% of bases across our N2 Bristol genome were aligned to our CB4856 Hawaiian genome assembly, and more than 92.2% of bases within alignments were syntenic (Table 1). Analysis of universal single-copy orthologs (Simão *et al*. 2015; Manni *et al*. 2021) in our *de novo*  N2 Bristol and CB4856 Hawaiian genomes revealed >98% completeness (Supplementary Fig. 1) and validated that our assemblies are high quality.

### *De novo* genome assemblies of the N2 Bristol and CB4856 Hawaiian isolates enhance detection of genomic variation

To comprehensively detect genomic variations between the N2 Bristol and CB4856 Hawaiian strains, we performed sequence alignments and comparisons with our reference genomes that were assembled using both short- and long-read sequencing technology and the same assembly method. First, we aligned our CB4856 Hawaiian short-reads to our N2 Bristol assembly to quantify SNPs and indels (Supplementary Data Files 1 and 2). This analysis revealed a total of 337,584 SNPs and 94,503 indels across the genome, of which 246,298 SNPs and 73,789 indels are homozygous and not segregating in our populations of each strain (Table 1; Fig. 1c and d). Similar to previous observations (Thompson *et al*. 2015), we note a few highly variable regions with a greater density of SNPs and short indels in the center regions of the autosomes, particularly on chromosomes *IV* and *V* (Fig. 1c and d). We also find that many SNPs and indels overlapped with gene annotations, particularly on chromosomes *I* and *III* where more than 60% of SNPs and indels overlap with gene sequences (Fig. 1e and f). Taken together, our detection of SNPs and indels indicate an abundance of sequence variation.

To identify large sequence variants and chromosome rearrangements, we used whole-genome alignments as opposed to the alignment of sequencing reads with our reference genomes (see *Methods*). This analysis identified a total of 4,364 SVs (Supplementary Data Files 3 and 4, example visualizations in Supplementary Fig. 4), which are categorized as insertions, deletions, and other chromosomal rearrangements spanning at least 50 bp (Table 1). We also identified 1,174 HDRs (Goel *et al*. 2019) across the genome. HDRs are defined here as a class of variant called by SyRI describing regions of the genome over 50 bp in length that result in low-quality pairwise alignments due to the presence of multiple gaps and mismatches within reciprocal whole-genome alignments given specific scoring parameters (Goel *et al*. 2019). Importantly, the HDRs we report from the output of SyRI are distinct from the similarly named “hyper divergent regions” later characterized in the *C. elegans* genome (Lee *et al*. 2021), where they were defined differently as regions of at least 9 kb in length that display exceptional variant density relative to a genome-wide average following sequencing read alignments. Overall, >3.1% of the N2 Bristol genome (∼100.0 Mb) displayed variation from SNPs, indels, SVs, while HDRs cover ∼6.8% of the genome. Notably, SVs represent only 1.3% of the variants called between N2 Bristol and CB4856 Hawaiian, but account for over 83% of the total base pairs affected by SNPs, indels, and SVs combined (Table 1). Including heterozygous variants, our short-read analysis detected 3% more SNPs and 18% more indels than previously discovered using short-read assemblies of N2 Bristol and CB4856 Hawaiian (Thompson *et al*. 2015). Utilizing whole-genome alignments (Li 2018; Goel *et al*. 2019), we identified 985 more SV sites than previously reported when comparing a long-read CB4856 assembly to a short-read N2 assembly (Nattestad and Schatz 2016; Kim *et al*. 2019). This increased sensitivity in variant site detection highlights the power of combining long-read and short-read sequencing to create accurate genome assemblies for comparative genomic studies.

### Millions of base pairs affected by genomic structural variation and HDRs

**Fig. 2.**
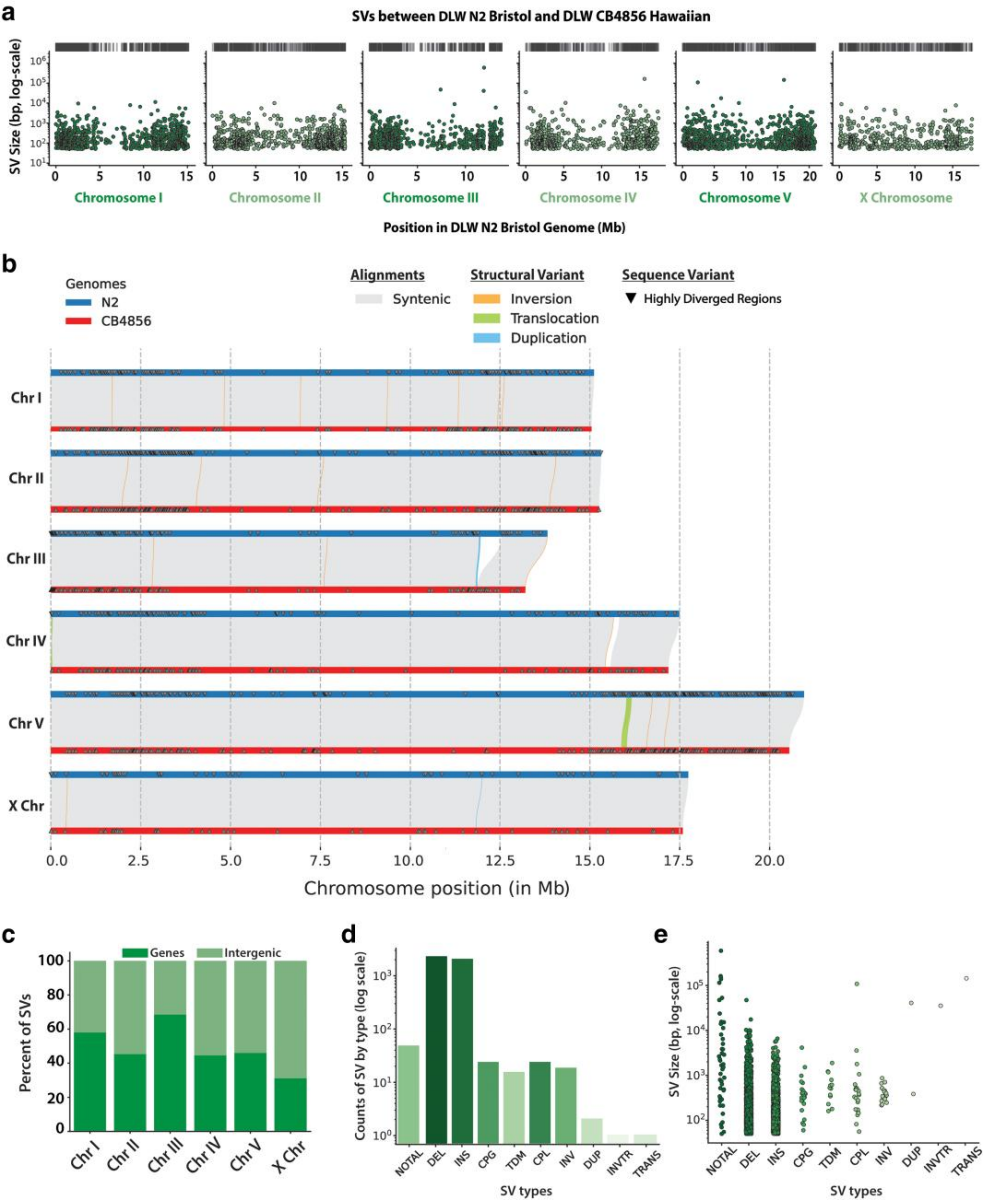
Genomic distribution and size of SVs between the N2 Bristol and CB4856 Hawaiian genomes. a) Scatter plots depicting the spatial distribution and size of SVs across the length of each chromosome. Black dashes above each scatter plot correspond to the genomic locations of SVs that are >20 kb in size. b) Chromosome alignment plot depicting syntenic regions between N2 Bristol and CB4856 Hawaiian, SVs, and HDRs. The width of lines showing SVs is proportional to their size. Only rearrangements 1 kb or greater in size are shown. c) Stacked bar plots showing the percentage of CB4856 Hawaiian SVs that overlap with intergenic and gene-coding regions of the N2 Bristol genome. d) Bar plots showing the number of each type of SV identified. e) Strip plots showing the log-scaled size distribution of SVs separated by type. For SV types: NOTAL, non-aligned regions; DEL, deletion; INS, insertion; CPG, copy gain in query genome; CPL, copy loss in query genome; TDM, tandem repeat region; INV, inversion; DUP, duplication; TRANS, translocation; INVTR, inverted translocation. For d and e), different colors only correspond to the different types of SV identified.

We next analyzed which regions of the genome were most affected by structural variation. Broadly, the distribution of SVs and HDRs across each chromosome resembles the genomic distribution of SNPs and indels (Fig. 2a and b). For both SVs and HDRs, we note that many of these sites reside on the arm-like domains of the autosomes, with the *X* chromosome displaying far fewer counts of structural variation and HDRs (Fig. 2a; Table 1). While most SVs range in size from 50 bp to 1 kb (Fig. 2a and e), we did find multiple instances of SVs that are 10 kb or greater in size on each chromosome, with some approaching hundreds of kilobases in size. Due to the large sizes of SVs, they have the potential to overlap with many coding sequences in the genome. In comparison, 52–69% of SNPs and indels overlapped with genes on the autosomes, while 49 and 42% of SNPs and indels overlapped with genes on the *X* chromosome, respectively. Thus, we asked whether SVs overlapped with coding regions at the same frequency as SNPs and indels. On the autosomes, 44.5–68.5% of SVs overlapped with gene regions compared with 31.1% of SVs overlapping genes on the *X* chromosome (Fig. 2c). Consistent with our previous analyses of SNPs and indels, SVs and HDRs are also present in greater numbers on the autosomes than on the *X* chromosome. Taken together, the sequence of the *X* chromosome appears more stable across the long divergence times separating the N2 Bristol and CB4856 Hawaiian isolates.

To assess which types of SVs are most prevalent in the genome, we determined the number and size of each type of SV we identified (Fig. 2d and e). Previous studies have identified many large insertions, deletions, and expansions/contractions of tandemly repeated regions (Kim *et al*. 2019; Zhang *et al*. 2022), but less is known about the genome-wide prevalence of other types of SVs such as inversions, duplications, and translocations. Most SVs identified were large insertions and deletions, with relatively fewer SVs being characterized as duplications or rearrangements like inversions (Fig. 2d). Among the SVs detected, we identified 47 non-alignable structures, 2 duplications, 18 inversions, and 2 translocations. Non-alignable regions (NOTALs) are highly diverged regions containing many repeats and low-complexity sequences that are inhibitory to whole-genome alignment. From our whole-genome alignments of the N2 Bristol and CB4856 Hawaiian genomes, the non-alignable regions between the 2 genomes comprise 1.39 Mb of sequence, ranged in size from 50 to 592 kb, and comprise <0.5% of coding genes in the Bristol genome. The SVs identified ranged in size from 50 bp to 592 kb (Fig. 2e), and HDRs ranged from 50 bp to 199 kb (Supplementary Data Files 3 and 4). Insertions and deletions are the most common type of SV, and their size distribution includes a greater proportion of 50–200 bp variants. The proportion of duplications and other rearrangements at magnitudes of 1–100 kb, in contrast, is much higher (Fig. 2e). We identified one 156 kb region in the N2 genome that was translocated upstream on the right end of CB4856 Hawaiian chromosome *V* (*V*: 15,871,614–16,027,614 bp), while the other translocation (38 kb in size) was found to be inverted near a telomere of CB4856 Hawaiian chromosome *IV* (*IV*: 176:38,447 bp). The largest duplication was found on Hawaiian chromosome *III* (*III*: 11,819,363–11,860,261). Together, our analyses detected a larger quantity and variety of SVs in the N2 and CB4856 genomes and further illuminated the contribution of large SVs and HDRs to genome evolution.

### Movement of DNA transposons between the N2 Bristol and CB4856 Hawaiian lineages

**Fig. 3.**
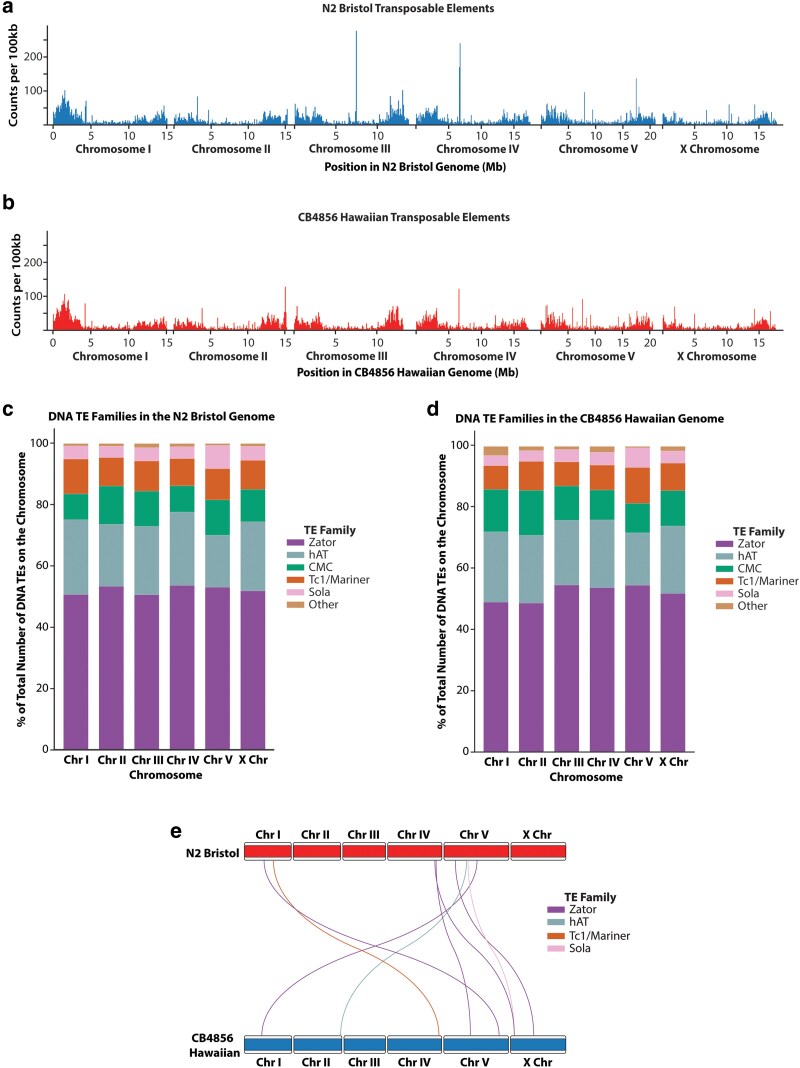
Genomic distributions of TEs in the N2 Bristol and CB4856 Hawaiian genomes. a) Histograms depicting the distributions of TEs across the N2 Bristol genome in 100-kb bins. b) Histograms depicting the distributions of TEs across the CB4856 Hawaiian genome in 100-kb bins. c and d) Stacked bar plot depicting the percent of total DNA TEs on N2 Bristol c) and CB4856 Hawaiian d) chromosomes accounted for by specific DNA transposon families. For TE families: CMC, CACTA, Mirage and Chapaev families; hAT, hobo and Activator families; Other, MITE, Novosib and Helitron families. e) Ideogram depicting the locations of individual DNA TEs that moved to different chromosomes between the N2 Bristol genome and the CB4856 Hawaiian genome. N2 Bristol chromosomes are represented by the boxes on the top, and CB4856 Hawaiian chromosomes represented by the boxes on the bottom. Each line represents an individual TE sequence, traced from its position on the N2 Bristol genome to its unique position on the CB4856 Hawaiian genome. TEs predicted to have translocated are colored according to transposon class.

To identify and locate known TE sequences in our N2 Bristol and CB4856 Hawaiian assembled genomes, we used a TE identification pipeline that applies an ensemble of programs to find all known RNA and DNA TE families (Riehl *et al*. 2022). We found that approximately 14.7% and 14.3% of our N2 Bristol and CB4856 Hawaiian assemblies, respectively, are composed of TE sequences (Table 2). For both genome assemblies, the distribution of TEs was concentrated in the terminal third, arm-like regions of each chromosome (Fig. 3a and b). Class II DNA TEs represented 96% of all TEs identified in each genome, and Zator elements are 52% of these class II DNA TEs present in each genome (Table 2; Fig. 3c and d). Further, we also found that our N2 Bristol genome has 20.6% more TEs from the *Tc1/mariner* family than our CB4856 Hawaiian genome assembly (Table 2).

**Table 2.** TEs identified in the N2 Bristol genome (this study) vs CB4856 Hawaiian genome (this study).

	N2 Bristol	CB4856 Hawaiian
Class I transposable elements (retrotransposons)	710 (2,688,730 bp)	776 (2,522,357 bp)
Gypsy	557 (2,195,895 bp)	592 (2,031,038 bp)
Copia	134 (472,195 bp)	161 (465,785 bp)
SINE	9 (2,146 bp)	9 (1,945 bp)
ERV	7 (8,280 bp)	6 (7,569 bp)
LINE	3 (10,214 bp)	8 (16,038 bp)
Class I intrachromosomal transpositions*^a^*	0	
Class I interchromosomal transpositions*^a^*	0	
Class II transposable elements (DNA transposons)	17,682 (12,055,357 bp)	17,310 (11,606,010 bp)
Tc1/Mariner	1,870 (1,298,386 bp)	1,550 (1,131,443 bp)
hAT	3,999 (3,988,461 bp)	3,818 (3,725,667 bp)
CMC	1,679 (3,138,647 bp)	2,011 (3,260,455 bp)
Zator	9,159 (3,009,341 bp)	8,980 (2,907,391 bp)
Novosib	46 (12,060 bp)	28 (12,088 bp)
Helitron	39 (368,980 bp)	43 (329,238 bp)
Sola	821 (226,645 bp)	699 (196,797 bp)
MITE	69 (12,837 bp)	181 (42,931 bp)
Class II interchromosomal movement*^a^*	8	
Class II intrachromosomal movement*^a^*	9	

^a^All TE sequences with predicted transpositions are relative to the N2 Bristol genome.

Since the N2 Bristol and CB4856 Hawaiian lineages were geographically isolated for thousands of generations, we sought to utilize our new TE annotation set to identify individual transposition events that occurred over the course of divergence between the 2 strains. Transposon movement is often characterized by “local hopping” and reintegration into site. Depending on the TE family in question, transposition intervals have been recorded at the kilobase and megabase magnitudes (Guimond *et al*. 2003; Muñoz-López and García-Pérez 2010). Using whole-genome alignments and the SNPs we previously defined between these 2 lineages, we identified specific TE sequences with unique polymorphisms that enables individual transposons to be tracked between the N2 Bristol and CB4856 Hawaiian genome assemblies. Of the 18,392 total TEs identified in the N2 Bristol genome, 9,377 TEs were uniquely identifiable by sequence polymorphisms. Among all copies of N2 Bristol TEs that are distinguishable by sequence polymorphism, only 1,535 of these sequences were also detectable in the CB4856 Hawaiian genome. Utilizing our reciprocal whole-genome alignments, we then looked for SNP-bearing TE sequences that appeared at coordinates outside of their alignment-matched corresponding start sites. While the vast majority of TEs were found to have not moved within either genome, we did track 8 class II DNA TEs that appeared on different chromosomes in the CB4856 Hawaiian genome (Fig. 3e; Table 3; Supplementary Data File 13). Specifically, we detected 5 Zator elements and 1 each of *Tc1/mariner*, Sola, and hAT elements on different chromosomes between the 2 lineages. Our initial analysis of TEs outside of their alignment-matched loci identified 33 putative intrachromosomal TE movements. Twelve of these TEs, however, share 100% identical flanking sequences, and although reciprocal genome alignments suggest intrachromosomal movement, analysis of the breakpoints surrounding these TEs makes assessing their movement challenging. In some of these cases, for example, we find TEs nested in other TE sequences of the same family, and they were excluded from further analysis due to the challenge of inferring whether they moved locally within the enveloping TE sequence (Supplementary Data File 13). To strengthen our analysis, we then further filtered our list of intrachromosomal TE movements to those whose flanking sequences were <90% identical by local pairwise sequence alignments. This stringency left 9 TE sequences with greater evidence for intrachromosomal movement. Their transposition distance varied greatly, with 4 of these TEs moving <1 kb from their original position and 1 transposition detected over 5.8 Mb away (Table 4; Supplementary Fig. 6). In the transposition event of 1 particular Zator element, we find that the CB4856 Hawaiian sequence is duplicated in 2 places on the same chromosome, and thus it is difficult to determine which event was a duplication vs the original transposition (see asterisks in Table 4; Supplementary Fig. 6). In our analysis of all detectable transposition events, Zator and hAT elements display the most movement among class II DNA TEs, though it is unclear whether other families are more active and their transposition events were not retained in the genome at the population level due to deleterious reintegration sites and/or natural selection. Overall, the landscape of TEs remains largely unchanged across the history of divergence between the N2 Bristol and CB4856 Hawaiian lineages.

**Table 3.** Intra- and interchromosomal movement of TEs by family.

Class II DNA TE family	Interchromosomal movements (8 total)	Intrachromosomal movements (9 total)
Zator	5	7
hAT	1	2
CMC	0	0
Tc1/Mariner	1	0
Sola	1	0

**Table 4.** Individual intrachromosomal movement of TEs.

Class II DNA TE family	Chromosome	Movement distance (bp)
*Zator^a^*	*II*	141
*Zator^a^*	*II*	453
*hAT*	*III*	60,251
*hAT*	*III*	301
*Zator*	*III*	12,526
*Zator*	*III*	3,136
*Zator*	*IV*	5,881,942
*Zator*	*IV*	348
*Zator*	*V*	562,801

^a^The exact sequence was found to be duplicated in two positions on the Hawaiian chromosome, precluding distinction of which copy arose from duplication versus transposition.

### Variants are enriched in TEs and depleted from genes and gene regulatory regions

**Fig. 4.**
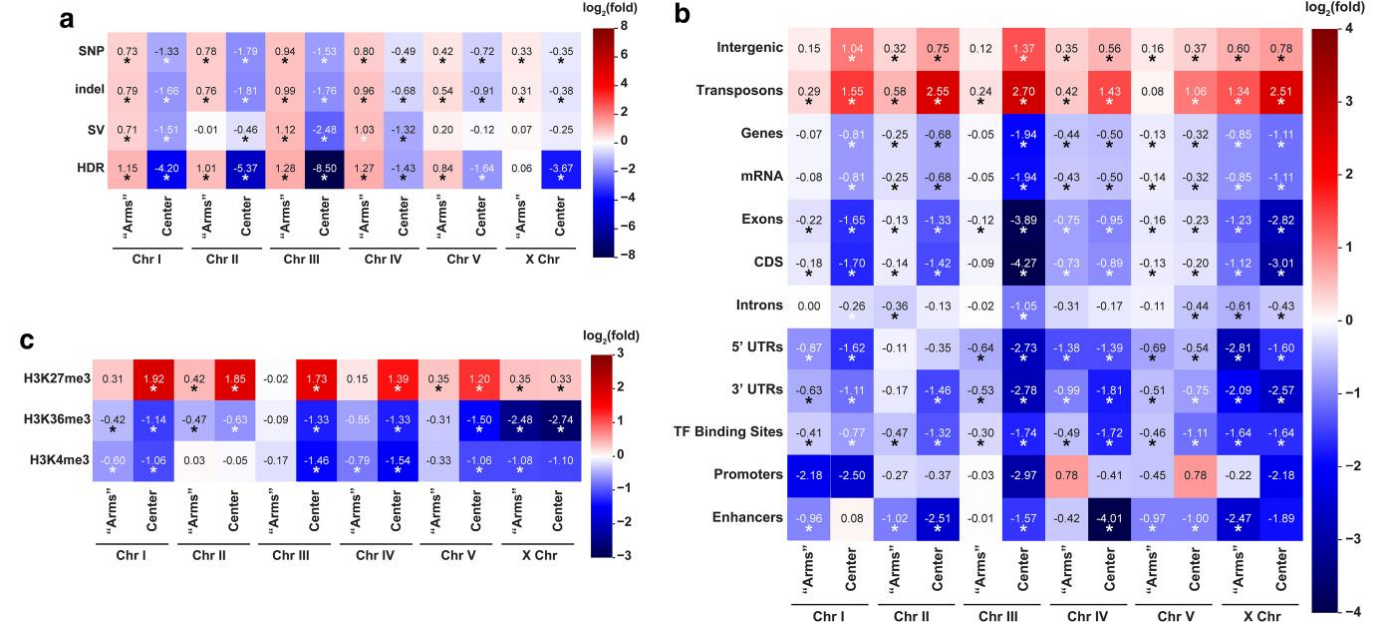
Genomic variants are depleted from coding regions and enriched in TEs and heterochromatin. a) Heatmap showing the Log_2_(fold) enrichment or depletion of each variant type in the “arms” (combined) or central region of each chromosome. b) Heatmap showing the Log_2_(fold) enrichment or depletion of all variant types (combined) in intergenic regions, TEs, genes, sub-gene annotations, and regulatory sequences. c) Heatmap showing the Log_2_(fold) enrichment or depletion of all variant types (combined) in different chromatin profiles. ChIP-seq data were taken from germline-specific datasets and calculated with MACS3 (see *Methods*). Asterisks below values indicate that the degree of overlap between variants and each annotation is significantly different (*P* < 0.05 by hypergeometric test) than the distribution of overlaps generated by 10,000 simulated null distributions.

Prior studies report has detailed punctuated regions of sequence divergence at the scale of SNPs and indels (Thompson *et al*. 2015; Kim *et al*. 2019); therefore, we wanted to confirm the enrichment of all variant sites in the “arm-like” regions of each chromosome. Indeed, we found that over 78% of SNPs, indels, SVs, and HDRs are found in the arm-like regions relative to the center region of each chromosome (Fig. 4a; genome-wide averages: 75.12% of SNPs, 78.24% of indels, 71.39% of SVs, 90.77% of HDRs). To determine whether the enrichment of SNPs, indels, and SVs in the chromosomal arm-like regions was significant, we compared the observed distribution of each variant category with random permutations of each category of variant (Heger *et al*. 2013). SNPs, indels, and HDRs on the autosomes were significantly enriched in the arm-like regions (Log_2_(fold) values for SNPs: 0.42–0.94; indels: 0.54–0.99; HDRs: 0.84–1.28; *P* < 0.001 by hypergeometric test). SVs, however, were only significantly enriched on the arm-like regions of autosomes *I*, *III*, and *IV* (Log_2_(fold) values 0.71, 1.12, and 1.03, respectively; *P* < 0.001 by hypergeometric test). The enrichment of all variants on the arm-like regions of the *X* chromosome was slightly weaker, and only significant for SNPs and indels (Log_2_(fold) values 0.33 and 0.31, respectively; all *P* < 0.05 by hypergeometric test). Taken together, we find that all types and sizes of genomic variants generally share the “arms”-vs-“center” distribution pattern across each chromosome.

We next tested whether variants accumulate in intergenic vs genic sequences. As an aggregate, variants are enriched in the intergenic regions of almost all chromosomal domains regardless of whether they are in the arm-like or center-like region of the chromosome (Fig. 4b). To determine what other kinds of sequences within intergenic regions may be driving this effect, we measured the overlap of our variant dataset with our transposon annotations. We find a consistent and much stronger enrichment trend of all variants specifically within TE sequences (Fig. 4b), and this trend is upheld when SNPs, indels, or SVs are analyzed separately (Supplementary Fig. 2). SVs were markedly more enriched in TEs (Log_2_(fold) values 0.39–2.84; *P* < 0.05), whereas SNPs and indels were relatively modest in their enrichment in these regions (Log_2_(fold) values −0.18 to 0.89; *P* < 0.05). SVs account for most of the base pairs affected by variation between the N2 Bristol and CB4856 Hawaiian genomes, and ∼32% of the base pairs in SVs reside within TE sequences. Taken together, genomic variants (particularly SVs) are enriched in intergenic TE sequences and thus TE-dense regions are the most prone to changes in genome structure.

To assess how gene and sub-gene annotations contribute to the accumulation, we analyzed the overlap of all variants in these regions. Based on our remapped gene annotations (see *Methods*), ∼61.8% of the N2 Bristol genome is comprised of gene sequences, with exons and introns representing 28.6 and 33.2% of the genome, respectively. Thus, if variant sites were uniformly distributed across the genome, then we would expect corresponding proportions of variants to overlap within each annotation. While we did find that many variants do overlap with gene sequences, our more detailed enrichment analyses find that these are likely occurring within intronic sequences (Fig. 4b; Supplementary Fig. 2). Broadly, we find significant depletions of variants in coding sequences on all chromosomes (Fig. 4b). Short indels under 50 bp, display the strongest depletion from CDS and exon sequences on every chromosomal region (Log_2_(fold) values −1.32 to −3.65; all *P* < 0.05, Supplementary Fig. 2b). To summarize, genes have fewer variants than expected by random chance and the depletion of variants in genomic annotations is the strongest in the central regions of each chromosome where there are many highly conserved, essential genes (*C. elegans* Sequencing Consortium 1998).

Since each variant type displayed significant depletions in coding regions of the genome, we then assessed whether regulatory and other intergenic sequences outside of genes were also depleted for genomic variation. Our aggregate variant dataset revealed significant depletions of variants in TF binding sites on every chromosomal domain (Log_2_(fold) values −0.3 to −1.74; all *P* < 0.05). Notably, enhancers were significantly more depleted of variants (Log_2_(fold) values −0.96 to −4.01; for all *P* < 0.05) than promoter sequences. SVs were notably depleted at TF binding sequences on every chromosome (Log_2_(fold) values −0.56 to −1.93). Of all variant types, the depletion of variants in these regions appears to be driven by SVs. SVs were consistently depleted from TF binding sites on every chromosomal domain (Log_2_(fold) values −0.89 to −2.61; all *P* < 0.05). In conclusion, gene regulatory sequences are depleted of variants, and SVs are remarkably absent in these regions.

### Genomic variation is enriched in silenced heterochromatic regions of the genome

Increasing evidence supports the role of hierarchical chromosome structures, such as chromatin, in contributing to the emergence and accumulation of genomic variants (Prendergast *et al*. 2007; Makova and Hardison 2015). Chromatin, the hierarchical organization of DNA around histone proteins, can adopt DNA-accessible or dense, inaccessible conformations to regulate gene/TE silencing vs gene expression (Rando and Winston 2012; Ho *et al*. 2014; Lawson *et al*. 2023). To test whether specific chromatin modifications and their associated chromatin states are associated with the accumulation of variants in different genomic regions, we measured how often variants overlapped with peaks of canonical heterochromatic and euchromatic modifications present in germline cells. Histone H3 lysine 4 trimethylation (H3K4me3) and histone H3 lysine 36 trimethylation (H3K36me3) are euchromatic modifications associated with transcribed genes in the germline (Liu *et al*. 2005; Pokholok *et al*. 2005; Mikkelsen *et al*. 2007; Rando and Winston 2012; Ho *et al*. 2014). In contrast, chromatin modifications like histone H3 lysine 9 trimethylation (H3K9me3) and histone H3 lysine 27 trimethylation (H3K27me3) mark heterochromatic and thus transcriptionally inactive regions, such as transposons, silenced genes, and intergenic regions, which all have lower DNA accessibility (Zhang *et al*. 2007; Connolly *et al*. 2013; Jamieson *et al*. 2013; Ho *et al*. 2014; Basenko *et al*. 2015; Lewis 2017). Our analysis reveals that variants are highly enriched in H3K27me3 heterochromatin in the central region of each autosome (Fig. 4c; Log_2_(fold) values 1.2–1.92; all *P* < 0.05). In contrast, our aggregate variant set shows a depletion of variants from both H3K36me3 and H3K4me3 euchromatic regions in the central region of each autosome, except H3K4me3 regions on chromosome *II* (Log_2_(fold) values −0.63 to −1.54; all *P* < 0.05). Notably, the *X* chromosome showed a similar trend, although magnitude of association on the “arms” vs “center” is roughly the same in contrast to the centers of autosomes showing the strongest effects (Fig. 4c). Each variant type considered separately does not perfectly recapitulate this trend (Supplementary Fig. 3). Taken together, variants are broadly enriched and accumulate in heterochromatic, silenced regions of the genome.

## Discussion

Detection and characterization of sequence variation between individuals or across species is fundamental to our functional understanding of genomic elements and the consequences of variation. Since the first draft of the *C. elegans* genome was released in 1998, the HDRs N2 Bristol and CB4856 Hawaiian have been used extensively for comparative genomics studies (*C. elegans* Sequencing Consortium 1998; Koch *et al*. 2000; Wicks *et al*. 2001; Maydan *et al*. 2010; Andersen *et al*. 2012; Lee *et al*. 2021). The combined usage of short-read and long-read sequencing to assemble genomes and to compare them has both increased the quality of reference genomes as well as enhanced the genome-wide detection of sequence variants, new genes, and new genomic regions (Kim *et al*. 2019, 2021; Sarsani *et al*. 2019; Yoshimura *et al*. 2019). In this study, we generate *de novo* assemblies for the N2 Bristol and CB4856 Hawaiian *C. elegans* isolates from our lab lineage using short-read and long-read sequencing. Our examination of the many types of genomic variants that arise in these diverged strains emphasizes the role that specific sequences and their associated chromatin structures have in shaping the evolution of genome structure across large timescales. Further, these genomes will serve as additional tools for future comparative genomics studies, especially in the functional characterization of structural variations identified through whole-genome alignments.

### Highly variable arm-like domains on *C. elegans* chromosomes

The arm-like regions of *C. elegans* chromosomes exhibit a striking degree of variation that is highly correlated with large domains of increased recombination, which is a pattern observed in many species (Rockman and Kruglyak 2009; Andersen *et al*. 2012; Kern and Hahn 2018; Lee *et al*. 2021). In *C. elegans*, these divergent autosomal arm-like domains coincide with a disproportionate fraction of newer, rapidly evolving genes when compared with the center regions of each chromosome, which house highly conserved essential genes (*C. elegans* Sequencing Consortium 1998; Kamath *et al*. 2003). The development of new tools to detect larger structural variations through alignment of assemblies or long sequencing reads has revealed many SVs on the chromosomal arm-like domains (Kim *et al*. 2019; Mahmoud *et al*. 2019). The fact that SVs are enriched in the arm-like regions, which also display elevated levels of recombination, is notable given the fact that large SVs such as inversions are typically inhibitory to recombination (Miller *et al*. 2019). The arm-like regions of *C. elegans* chromosomes are enriched for many repetitive elements, including TEs, tandem repeats, and low-complexity repeat sequences (*C. elegans* Sequencing Consortium 1998; Surzycki and Belknap 2000). The presence of many SVs in the arm-like regions could be due to errors in double-strand DNA break repair and heterologous recombination in regions adjacent to highly repetitive sequences, thereby causing chromosomal rearrangements. Similar rearrangement events are known to contribute to many human genomic disorders like Prader–Willi syndrome or Charcot–Marie–Tooth disease (Stankiewicz and Lupski 2010; Carvalho and Lupski 2016). Future investigations assessing the occurrence of SVs adjacent to highly repetitive regions and sites of homologous recombination will increase our understanding of how differences in genomic organization arise between divergent *C. elegans* lineages.

### An emergent role of new TE superfamilies in genomic structural variation

Our data indicate that TEs and their mobility within and between chromosomes may contribute to genomic structural variation. While Sola and Zator elements are relatively recent in their discovery within *C. elegans* and other eukaryotic genomes (Bao *et al*. 2009; Riehl *et al*. 2022), our data suggest there may be many active TE copies in these families, particularly Zator elements. Historically, much attention has been given to the impact of *Tc1/Mariner* transposition on genomic architecture, but the contribution of the more recently identified Zator elements to changes in genome structure and gene regulation merits further future investigation. Our analysis of TE mobility examines 2 endpoints across the long period of divergence between the Bristol and Hawaiian lineages. It remains unclear, however, whether many of these newly characterized TEs remain active and whether they contribute to the growing catalog of phenotypic variances displayed between different laboratory lineages of Bristol and Hawaiian *C. elegans*.

### The role of chromatin in shaping patterns of genomic variation

Our data shows that many SVs are retained in silenced, heterochromatic regions of the *C. elegans* genome. While natural selection may lead to a broad loss of SVs in coding regions of the genome due to their ability to negatively impact gene function, germline heterochromatin that overlaps facultatively silenced coding regions may also be slightly more susceptible to variant accumulation. New sequence variants and structural variations are the foundation of genome evolution. Short sequence variants (SNPs and indels) are the most common type of genomic variant, and studies have found that mutation rates for these short sequence variants are not equal across the genome (Wolfe *et al*. 1989; Hardison *et al*. 2003; Hodgkinson and Eyre-Walker 2011). Chromatin modifications and chromatin accessibility likely play a significant role in how variants accumulate in structurally distinct compartments of the genome (Makova and Hardison 2015). For example, differences in chromatin organization within somatic cancer cells can shape local heterogeneities in the mutation rate (Schuster-Böckler and Lehner 2012). Further, germline variants have much more profound impacts than somatic variants in shaping the genetic variation of individuals between populations, and they produce lasting effects on the evolution of genome structures within species (Yu *et al*. 2024). Thus, it remains possible that coding sequences that are silenced as facultative heterochromatin in germline cells may have a greater evolutionary potential if SVs and other variants are more often retained in these silenced regions. Our results suggest germline chromatin states may influence genome evolution, thereby serving as the foundation for future research to understand how distinct chromatin states directly affect the specific rates at which SNPs, indels, or SVs accumulate across the genome while still under selective pressures to eliminate deleterious alleles.

How might chromatin states and sequence context dictate where variations appear? Base substitutions and short indels that occur naturally in regions of open chromatin may be more likely to be repaired. DNA repair proteins and other factors that monitor and correct sequence mismatches are more likely to eliminate new mutations and variants if the region is physically accessible (Prendergast *et al*. 2007). Further, we found an abundance of variant sites nested within TE sequences, which also reside in heterochromatic domains. In the case of TEs, 1 hypothesis for this enrichment is that the accumulation of variants in in TEs could be protective of genome integrity. TE insertion into genes or regulatory sequences can be highly disruptive, and there are multiple mechanisms to silence their mobility to protect genome integrity (Lawson *et al*. 2023). For example, an enhanced mutation rate in TEs provides an alternative method to silence their movement since mutations could disrupt TE excision or expression (Lohe *et al*. 1997). TE sequences, therefore, could be more likely to accumulate both small sequence variants as well as structural variations. Taken together, the landscape of chromatin states that silence specific genes or TEs is likely contributing to patterns of sequence divergence and changes in genome structure.

### Conclusion

In summary, our *de novo* generation of genome assemblies of both N2 Bristol and CB4856 Hawaiian isolates reveals how specific regions of the genome, including large-scale repetitive regions, are more susceptible to accumulation of genetic variation and changes to genome structure. Our findings suggest that heterochromatic regions of the genome are susceptible to the generation of many small and large genomic variants. These silenced regions of the genome are likely strong contributors to the large-scale differences in genome structure between individuals as well as across populations, and these SVs may underpin a greater proportion of the phenotypic variation and genetic variation than previously appreciated. In conclusion, our comprehensive detection and analysis of sequence variations between 2 isogenic wild-type populations revealed how TEs and different chromatin states contribute to the evolution of genome structure.

## Supplementary Material

jkaf092_Supplementary_Data

## Data Availability

The PacBio long-read and the Illumina short-read data generated in this study have been submitted to the NCBI BioProject database (https://www.ncbi.nlm.nih.gov/bioproject/) under accession number PRJNA907379. Code for our transposon annotation and tracking method can be found on github (https://github.com/libudalab/transposon-tracking). Strains are available upon request. Supplementary materials are available on GSA FigShare at https://doi.org/10.25387/g3.28833551. Supplemental material available at G3 online.
