# Supplementary material for: Transposable elements and heterochromatic regions are enriched for structural variation and sequence divergence in the genome of wild-type Caenorhabditis elegans
Source: G3 (Bethesda). 2025 Apr 30;15(7):jkaf092. doi: 10.1093/g3journal/jkaf092 (PMC12239620; doi:10.1093/g3journal/jkaf092)
Supplement: jkaf092_Supplementary_Data [file jkaf092_supplementary_data.zip › 28833551/Supplemental_Figure_S1.pdf]

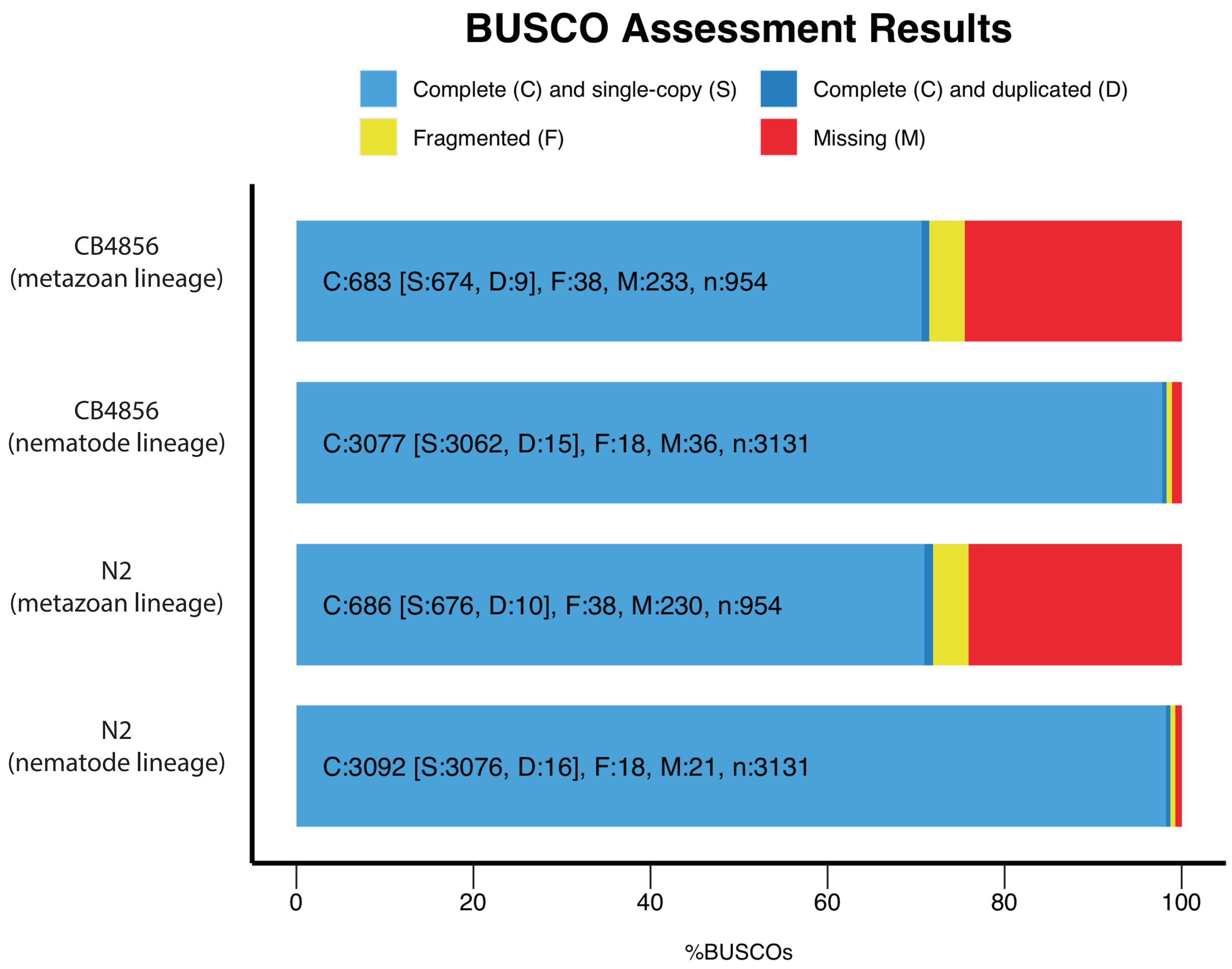

Supplemental Figure S1. BUSCO analysis of the N2 Bristol and CB4856 Hawaiian genome assemblies. The presence of orthologous genes from metazoan and nematode lineages are shown for each genome assembly. Each orthologous gene analyzed is depicted as either Complete (C, blues), Fragmented (F, yellow), or Missing (M, red). Complete orthologs are then further categorized as single-copy (S, light blue) or duplicated (D, dark blue).
