# Supplementary material for: Transposable elements and heterochromatic regions are enriched for structural variation and sequence divergence in the genome of wild-type Caenorhabditis elegans
Source: G3 (Bethesda). 2025 Apr 30;15(7):jkaf092. doi: 10.1093/g3journal/jkaf092 (PMC12239620; doi:10.1093/g3journal/jkaf092)
Supplement: jkaf092_Supplementary_Data [file jkaf092_supplementary_data.zip › 28833551/Supplemental_Figure_S3.pdf]

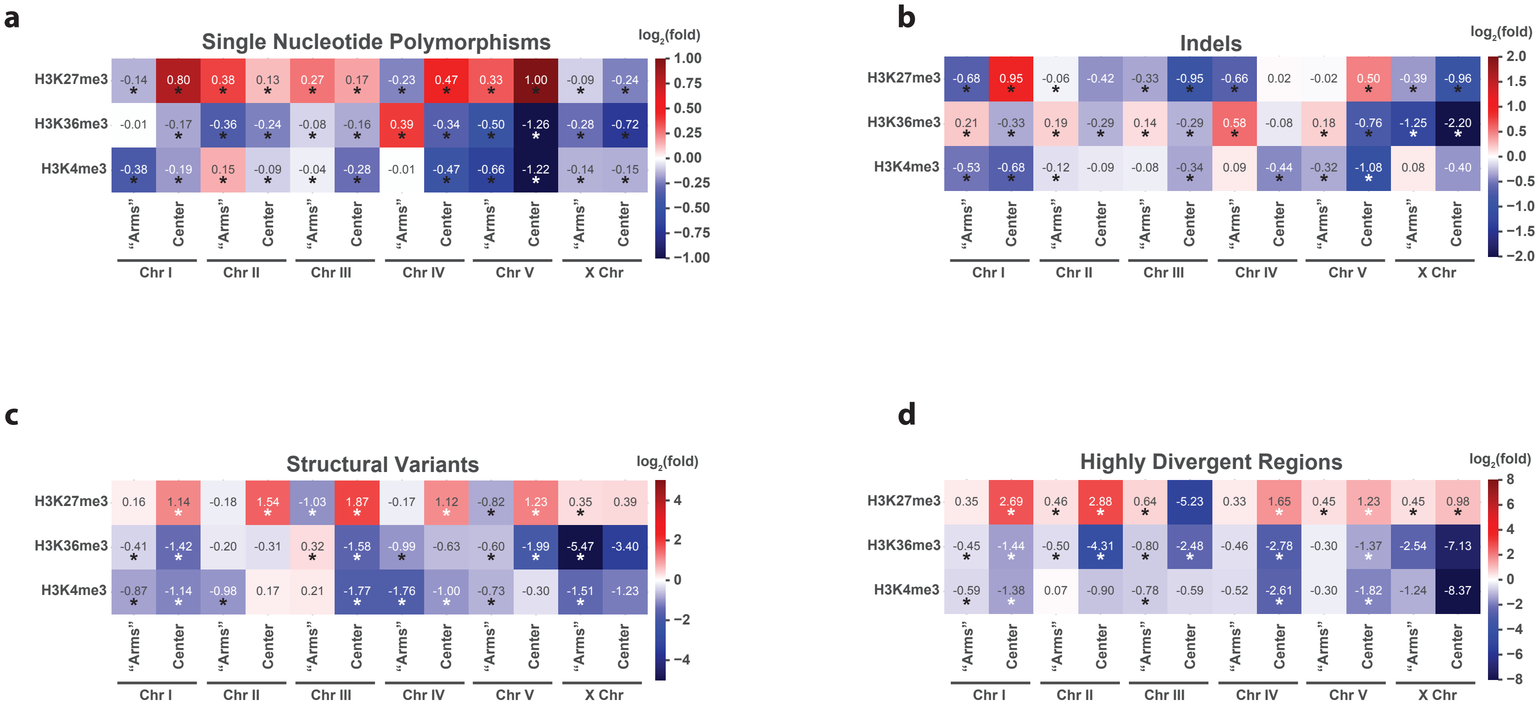

Supplemental Figure S3. Detailed associations of each variant type and HDRs in heterochromatin vs euchromatin. a) Heatmap showing the Log2(fold) enrichment or depletion of SNPs in different chromatin profiles. b) Heatmap showing the Log2(fold) enrichment or depletion of indels in different chromatin profiles. c) Heatmap showing the Log2(fold) enrichment or depletion of SVs in different chromatin profiles. d) Heatmap showing the Log2(fold) enrichment or depletion of HDRs in different chromatin profiles. ChIP-seq data were taken from germline-specific datasets and calculated with MACS3 (see methods). Asterisks below values indicate that the degree of overlap between variants and each annotation is significantly different that the distribution of overlaps generated by 10,000 simulated null distributions.
