# Supplementary material for: Transposable elements and heterochromatic regions are enriched for structural variation and sequence divergence in the genome of wild-type Caenorhabditis elegans
Source: G3 (Bethesda). 2025 Apr 30;15(7):jkaf092. doi: 10.1093/g3journal/jkaf092 (PMC12239620; doi:10.1093/g3journal/jkaf092)
Supplement: jkaf092_Supplementary_Data [file jkaf092_supplementary_data.zip › 28833551/Supplemental_Figure_S4.pdf]

a

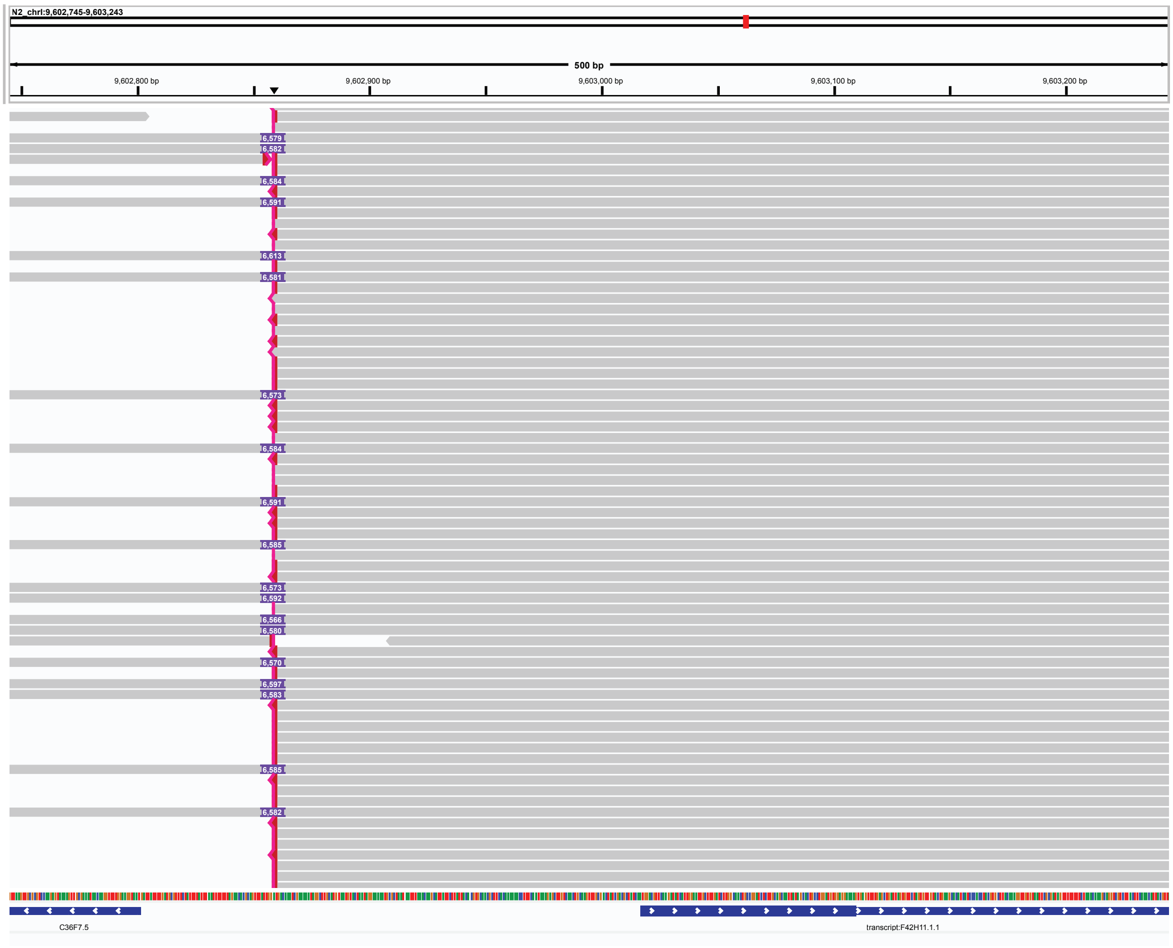
N2_chrI:9,602,745-9,603,243
500 bp
9,602,800 bp
9,602,900 bp
9,603,000 bp
9,603,100 bp
9,603,200 bp
6,579
6,582
6,584
6,591
6,613
6,581
6,573
6,584
6,591
6,585
6,573
6,592
6,566
6,580
6,570
6,597
6,583
6,585
6,582
C36F7.5
transcript:F42H11.1.1

b

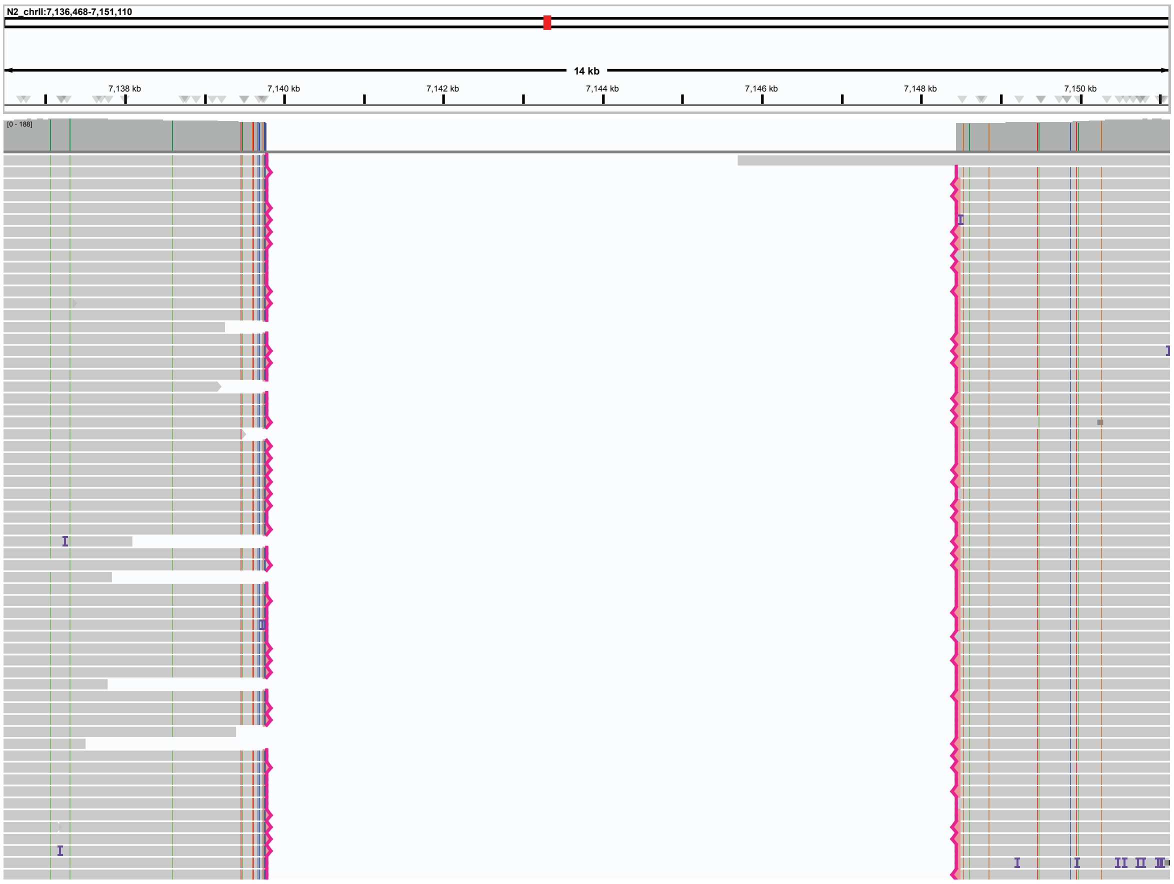
N2_chrII:7,136,468-7,151,110
14 kb
7,138 kb
7,140 kb
7,142 kb
7,144 kb
7,146 kb
7,148 kb
7,150 kb
[0 - 188]

c

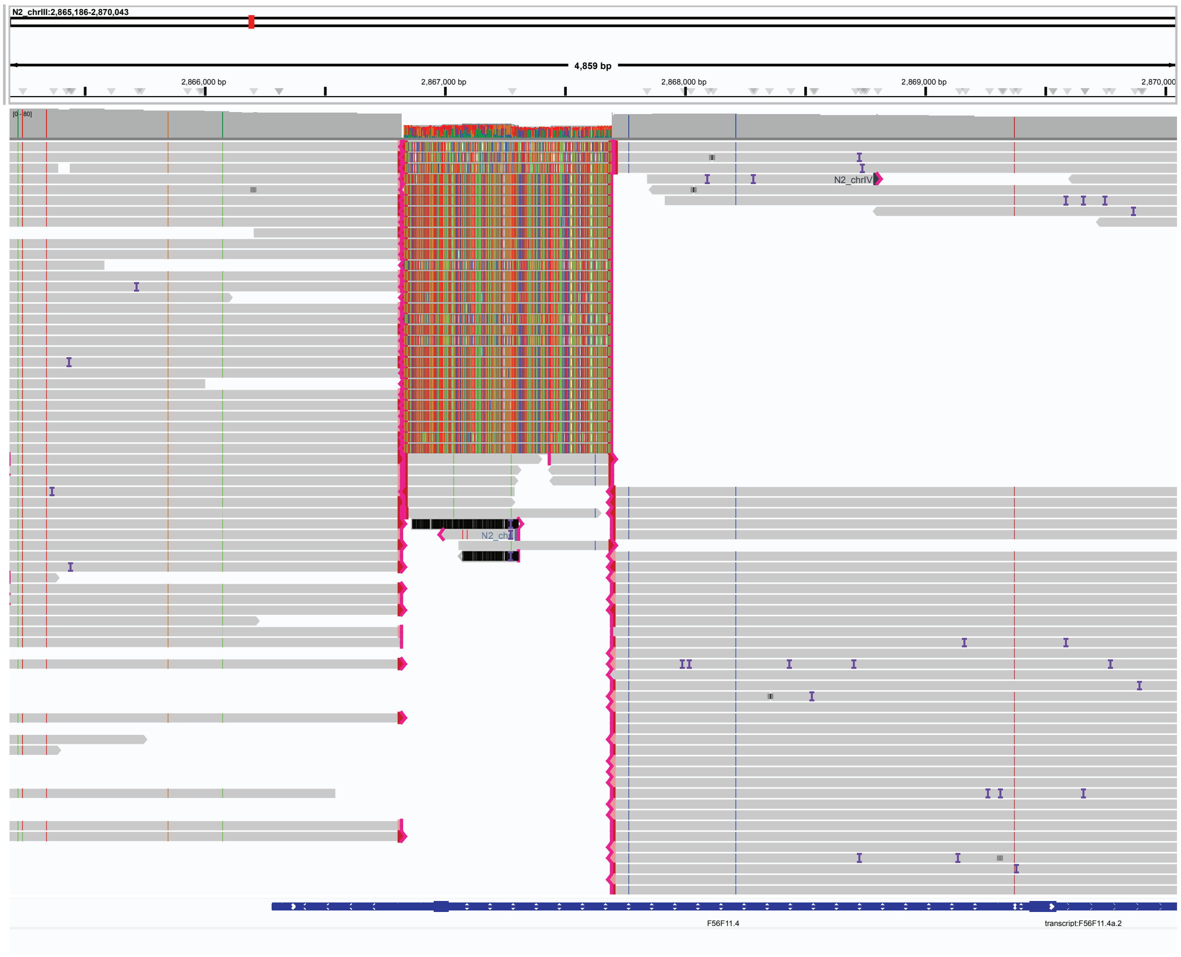
N2_chrIII:2,865,186-2,870,043
4,859 bp
2,866,000 bp
2,867,000 bp
2,868,000 bp
2,869,000 bp
2,870,000
N2_chrIV
F56F11.4
transcript:F56F11.4a.2

d

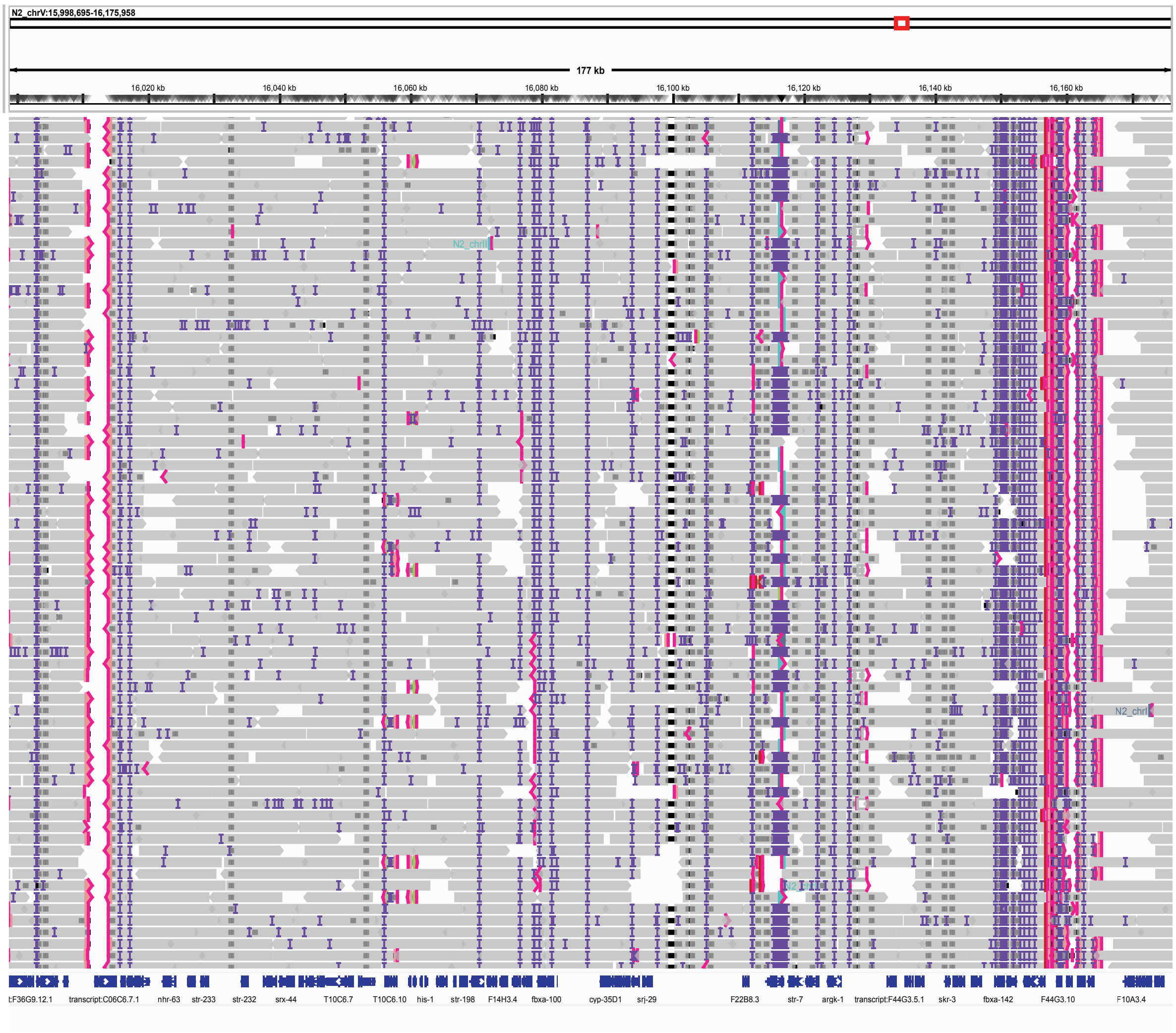
N2_chrV:15,998,695-16,175,958
177 kb
16,020 kb
16,040 kb
16,060 kb
16,080 kb
16,100 kb
16,120 kb
16,140 kb
16,160 kb
t:F36G9.12.1
transcript:C06C6.7.1
nhr-63
str-233
str-232
srx-44
T10C6.7
T10C6.10
his-1
str-198
F14H3.4
fbxa-100
cyp-35D1
srj-29
F22B8.3
str-7
argk-1
transcript:F44G3.5.1
skr-3
fbxa-142
F44G3.10
F10A3.4

e

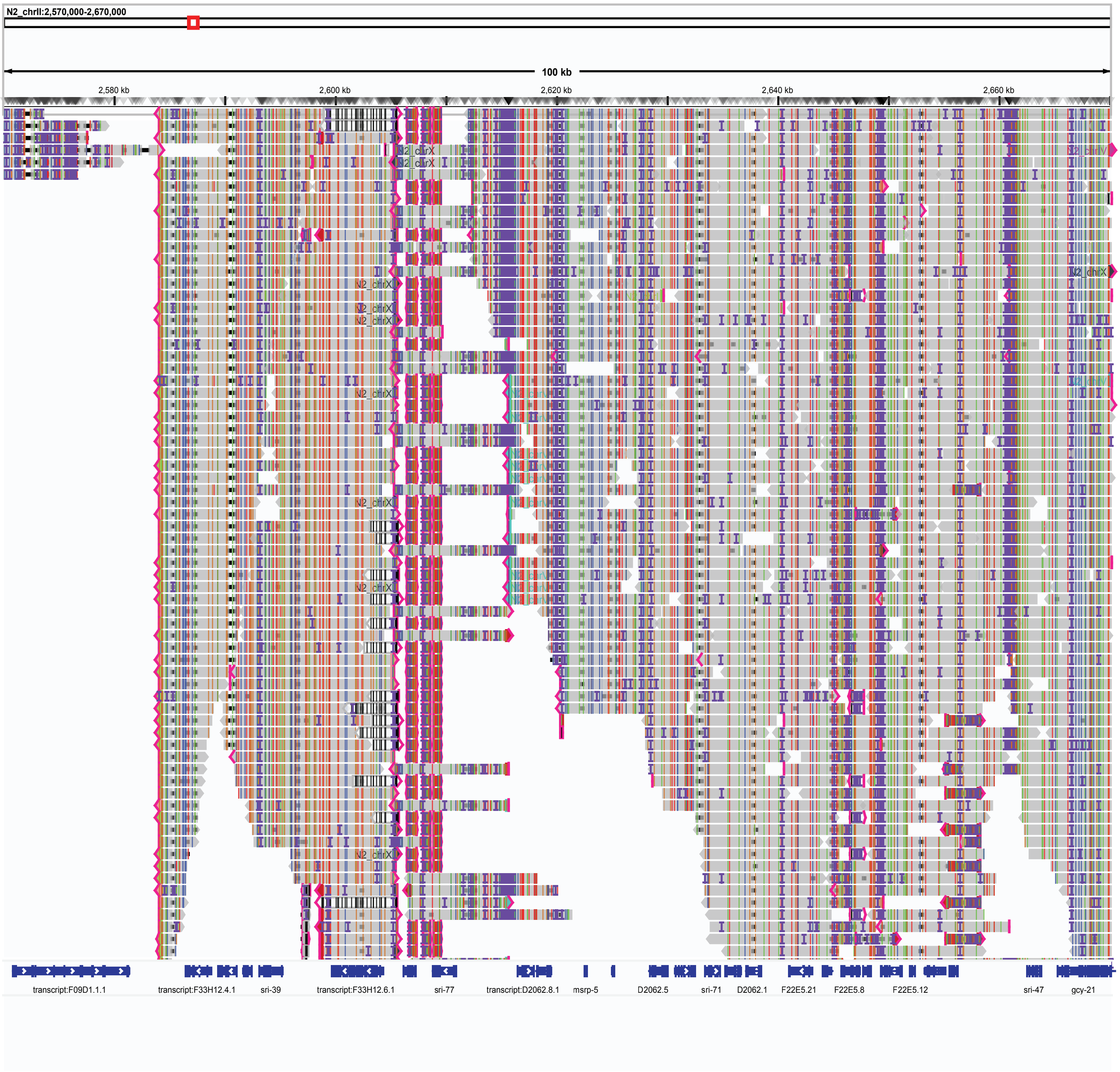


**Integrative Genomics Viewer (IGV) Visualizations of Structural Variants in the CB4856 Hawaiian genome.** a) An insertion over 6.5kb in length on chromosome I. b) A 55kb deletion on chromosome II. c) An 865bp inversion on chromosome III. d) A 144kb translocation on chromosome V. e) An 85kb Highly Diverged Region (HDR) on chromosome II. For all visualizations, PacBio sequencing reads from the CB4856 Hawaiian genome are aligned to the N2 Bristol genome. Gapped red markers in c-d indicate clipped reads at the site of each rearrangement's breakpoints. The track below the alignments indicate the presence of annotated genes in the N2 Bristol genome. All coordinates shown are relative to the N2 Bristol genome.
