# Supplementary material for: Transposable elements and heterochromatic regions are enriched for structural variation and sequence divergence in the genome of wild-type Caenorhabditis elegans
Source: G3 (Bethesda). 2025 Apr 30;15(7):jkaf092. doi: 10.1093/g3journal/jkaf092 (PMC12239620; doi:10.1093/g3journal/jkaf092)
Supplement: jkaf092_Supplementary_Data [file jkaf092_supplementary_data.zip › 28833551/Supplemental_Figure_S5.pdf]

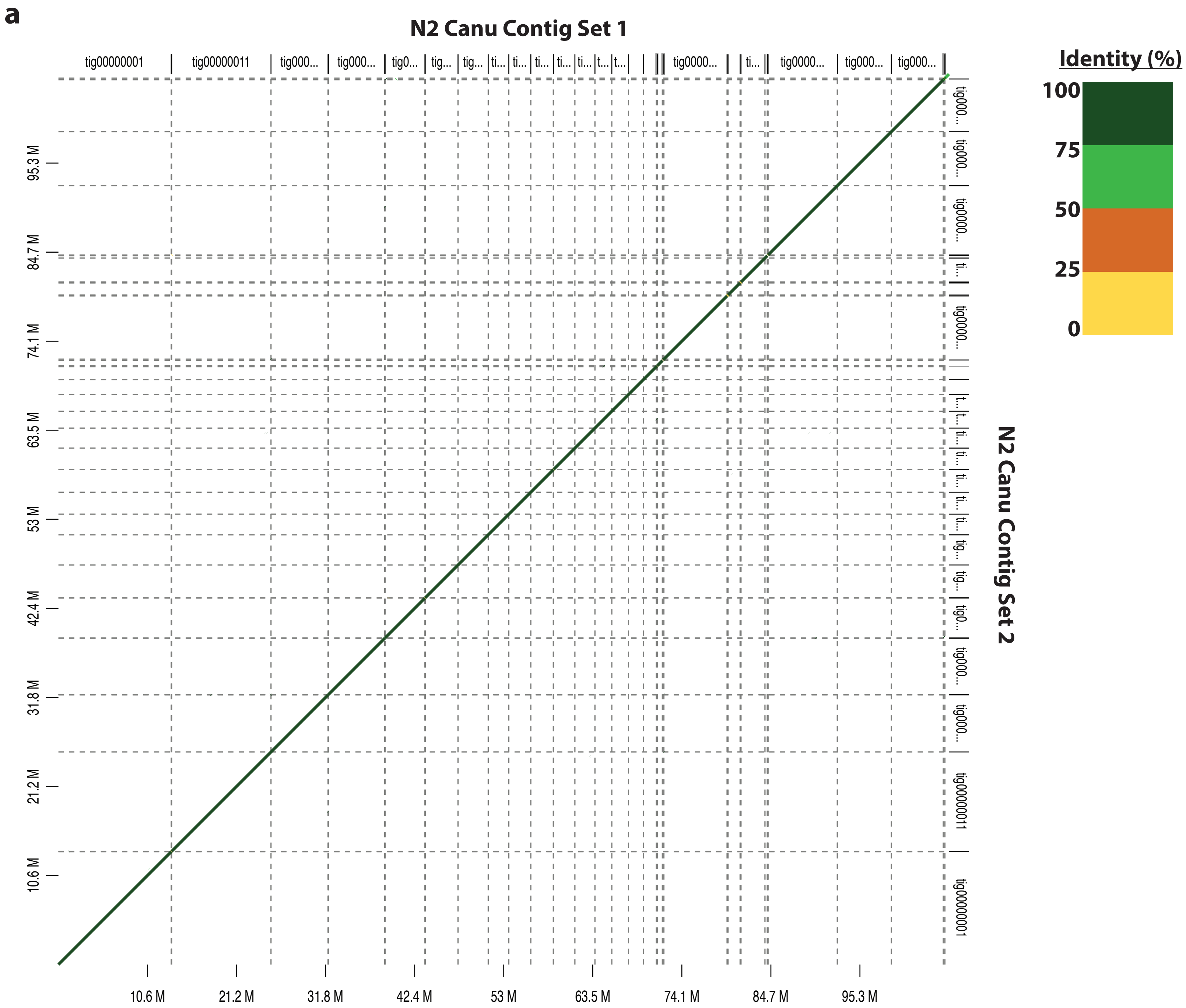
a
N2 Canu Contig Set 1
N2 Canu Contig Set 2
Identity (%)
100
75
50
25
0
tig00000001
tig00000011
10.6 M
21.2 M
31.8 M
42.4 M
53 M
63.5 M
74.1 M
84.7 M
95.3 M

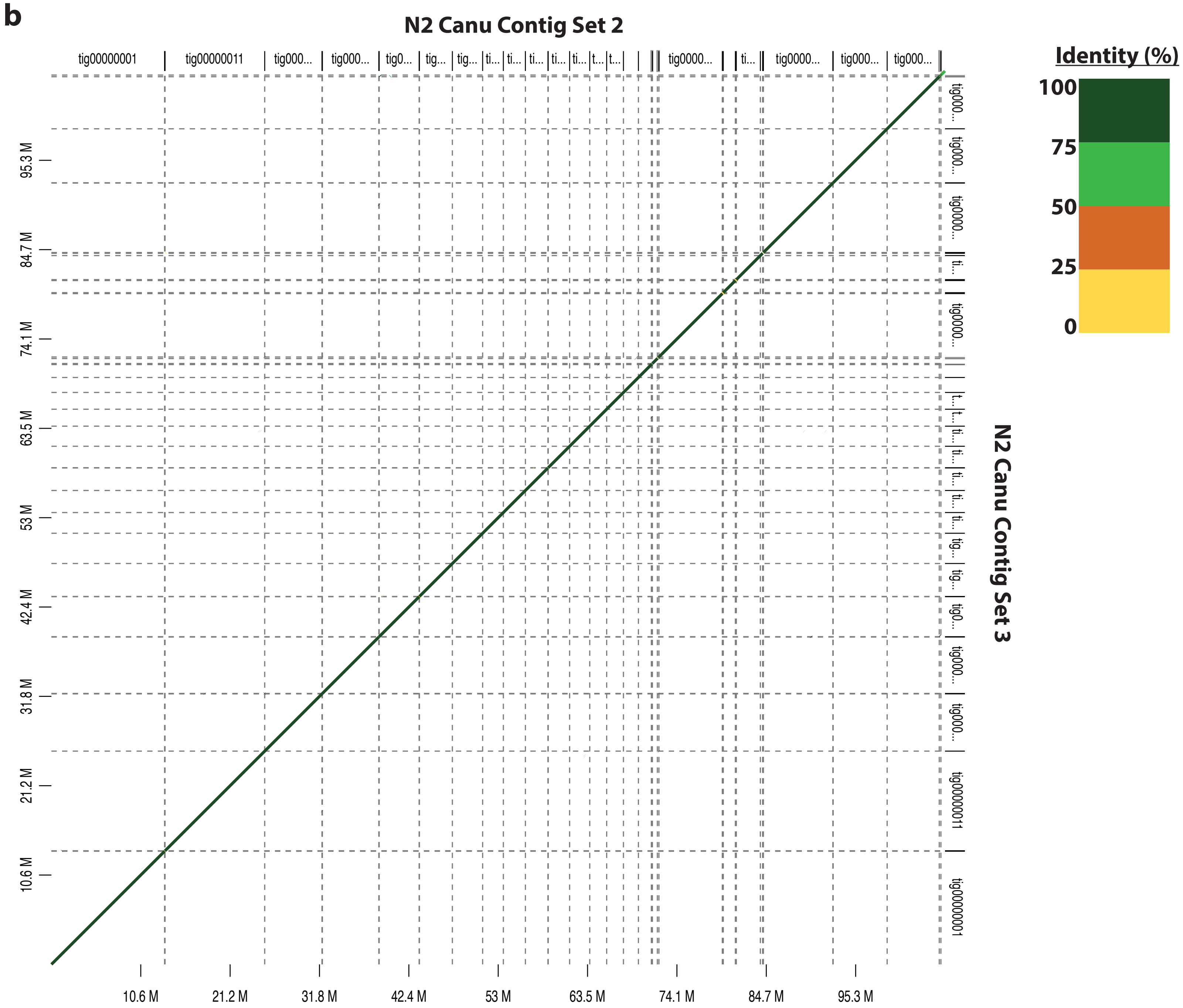
b
N2 Canu Contig Set 2
N2 Canu Contig Set 3
Identity (%)
100
75
50
25
0
tig00000001
tig00000011
10.6 M
21.2 M
31.8 M
42.4 M
53 M
63.5 M
74.1 M
84.7 M
95.3 M

c

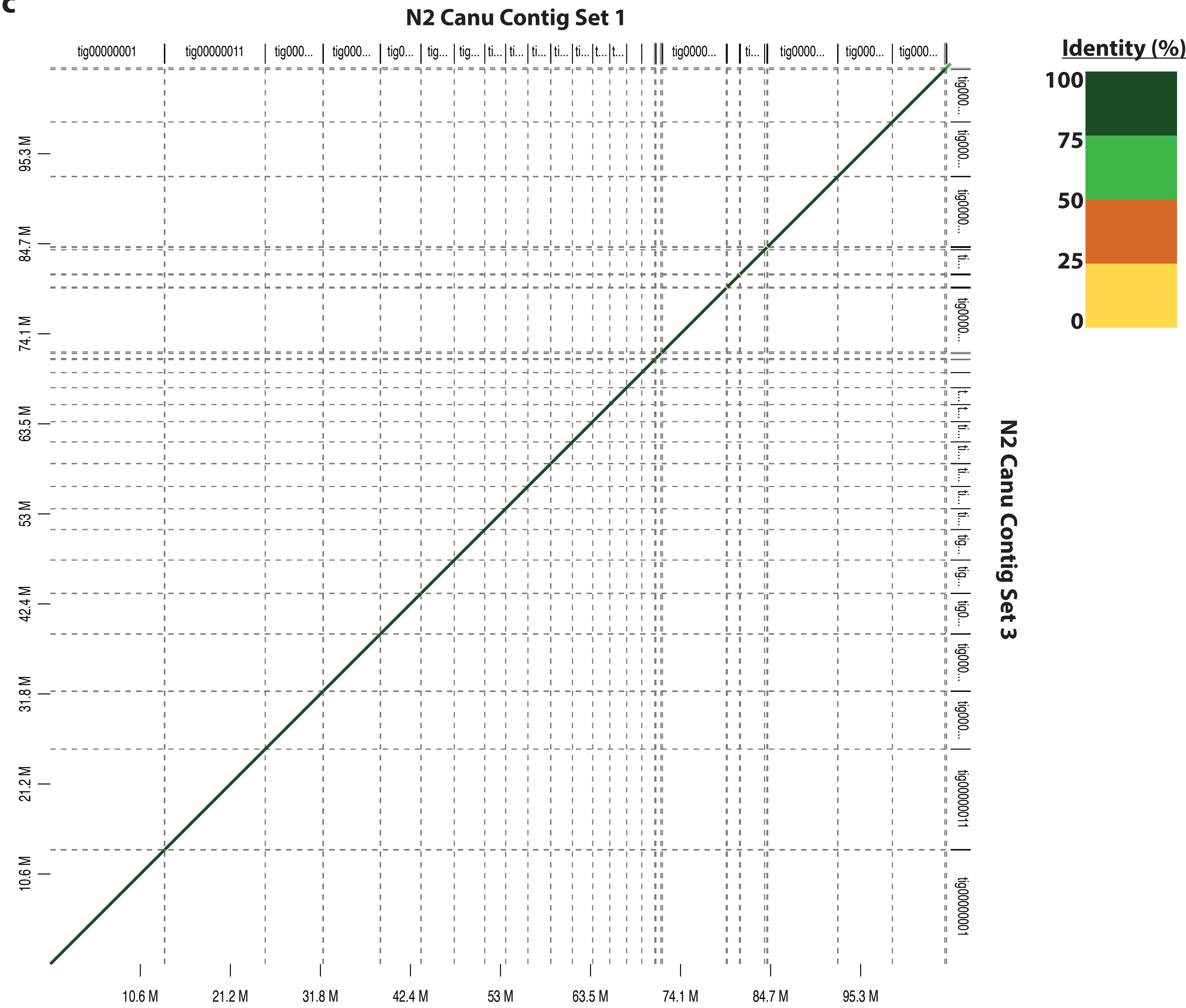

N2 Canu Contig Set 1
N2 Canu Contig Set 3
Identity (%)
100
75
50
25
0
tig00000001
tig00000011
10.6 M
21.2 M
31.8 M
42.4 M
53 M
63.5 M
74.1 M
84.7 M
95.3 M

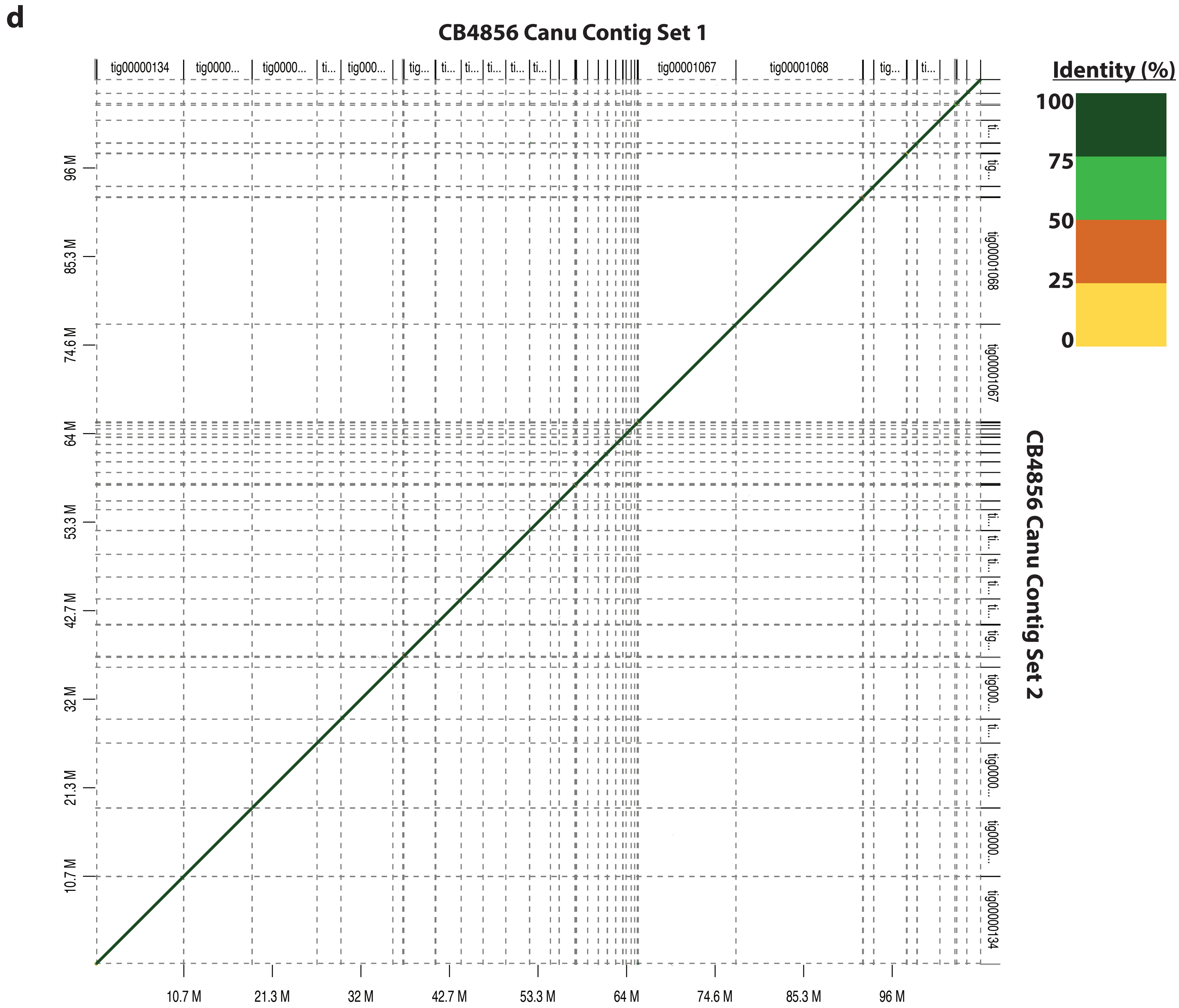
d
CB4856 Canu Contig Set 1
CB4856 Canu Contig Set 2
Identity (%)
100
75
50
25
0
tig00000134
tig00001067
tig00001068
tig00001068
tig00001067
tig00000134
10.7 M
21.3 M
32 M
42.7 M
53.3 M
64 M
74.6 M
85.3 M
96 M

e

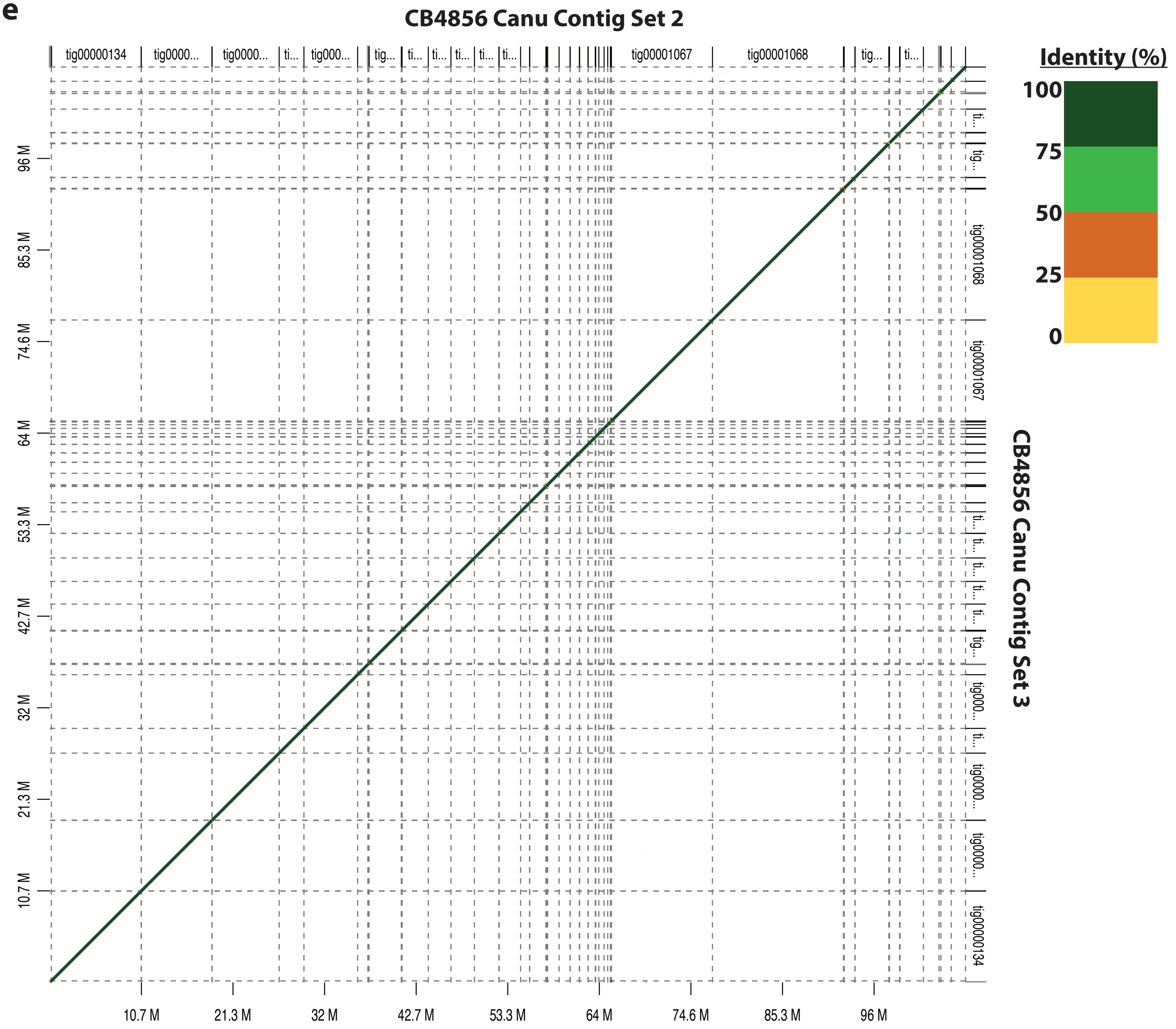
CB4856 Canu Contig Set 2
tig00000134
tig0000...
tig0000...
ti...
tig000...
tig...
ti...
tig00001067
tig00001068
tig...
CB4856 Canu Contig Set 3
tig00001068
tig00001067
tig000...
tig0000...
tig0000...
tig00000134
96 M
85.3 M
74.6 M
64 M
53.3 M
42.7 M
32 M
21.3 M
10.7 M
Identity (%)
100
75
50
25
0

f

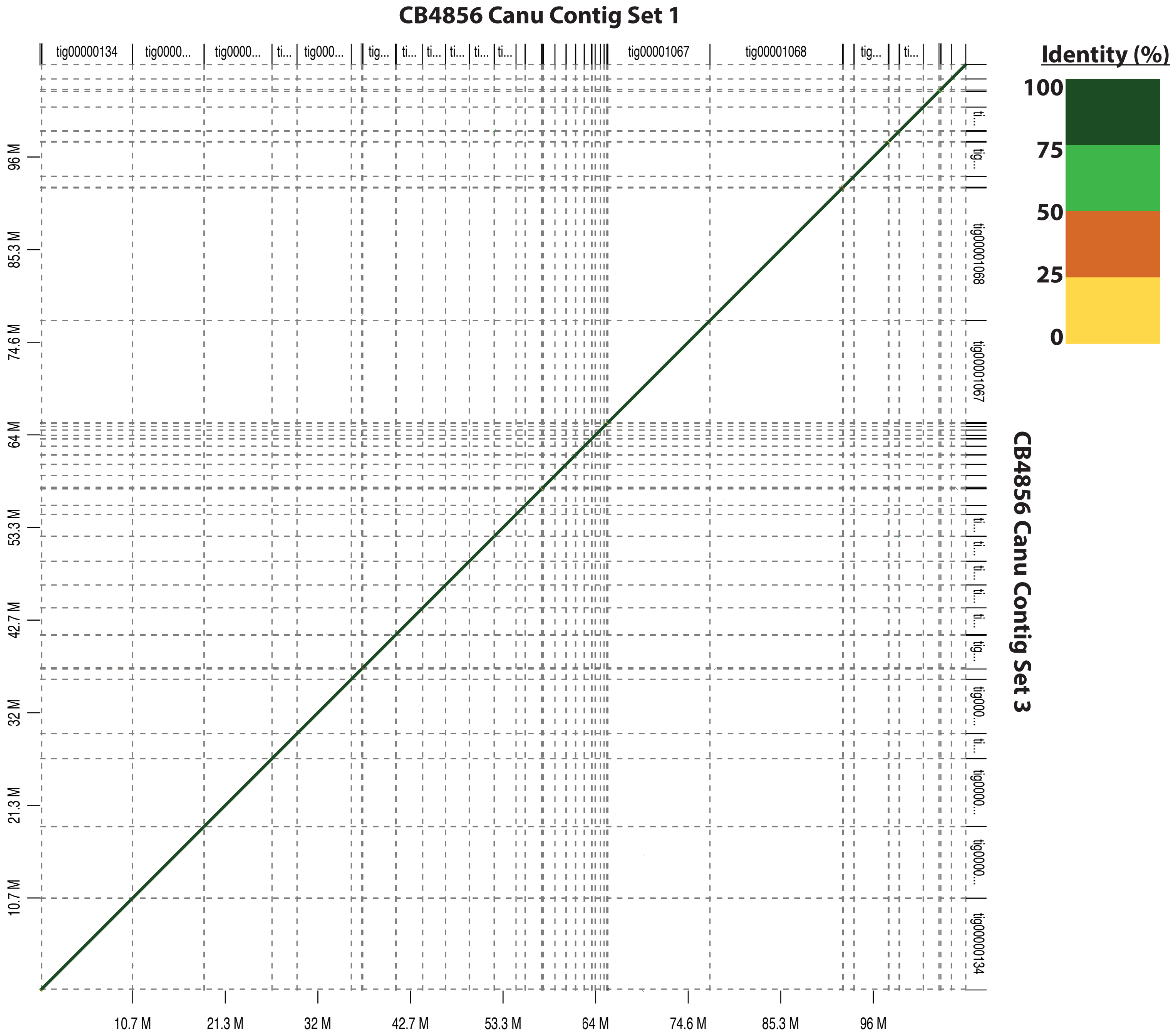

CB4856 Canu Contig Set 1
tig00000134
tig0000...
tig0000...
ti...
tig000...
tig...
ti...
ti...
ti...
ti...
ti...
tig00001067
tig00001068
tig...
ti...
Identity (%)
100
75
50
25
0
96 M
85.3 M
74.6 M
64 M
53.3 M
42.7 M
32 M
21.3 M
10.7 M
ti...
tig...
tig00001068
tig00001067
ti...
ti...
ti...
ti...
ti...
tig...
tig000...
ti...
tig0000...
tig0000...
tig00000134
CB4856 Canu Contig Set 3
10.7 M
21.3 M
32 M
42.7 M
53.3 M
64 M
74.6 M
85.3 M
96 M

Supplemental Figure S5. Pairwise alignments of contig sequences. a) Dotplot showing alignment and the percent of shared sequence identity for N2 Bristol contig set 1 versus N2 Bristol contig set 2. b) Dotplot showing alignment and the percent of shared sequence identity for N2 Bristol contig set 2 versus N2 Bristol contig set 3. c) Dotplot showing alignment and the percent of shared sequence identity for N2 Bristol contig set 1 versus N2 Bristol contig set 3. d) Dotplot showing alignment and the percent of shared sequence identity for CB4856 Hawaiian contig set 1 versus CB4856 Hawaiian contig set 2. e) Dotplot showing alignment and the percent of shared sequence identity for CB4856 Hawaiian contig set 2 versus CB4856 Hawaiian contig set 3. f) Dotplot showing alignment and the percent of shared sequence identity for CB4856 Hawaiian contig set 1 versus CB4856 Hawaiian contig set 3.
