# Supplementary material for: Transposable elements and heterochromatic regions are enriched for structural variation and sequence divergence in the genome of wild-type Caenorhabditis elegans
Source: G3 (Bethesda). 2025 Apr 30;15(7):jkaf092. doi: 10.1093/g3journal/jkaf092 (PMC12239620; doi:10.1093/g3journal/jkaf092)
Supplement: jkaf092_Supplementary_Data [file jkaf092_supplementary_data.zip › 28833551/Supplemental_Figure_S6.pdf]

a

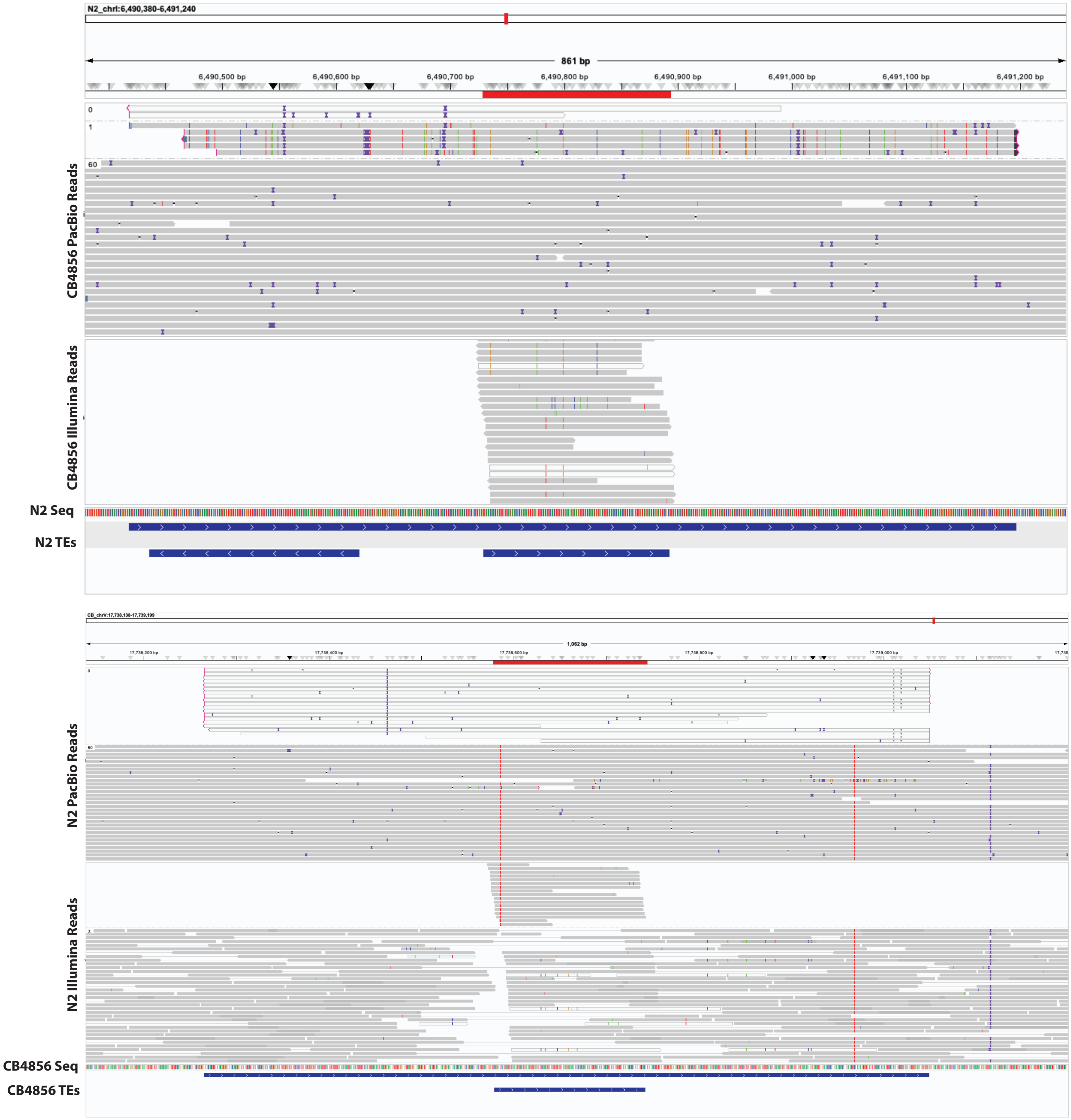


```
target            0 AAATACAAAACCTAAAC--TTTCCCAAACATGATACATATGCTGCTTAGATGCTGAAACTACCTGATTTTCATAACGAGACCGCTGAAAAAGTTTTGAGGTTTTCAAAATTCAACTTTTT
                  0 --|||||||||||||||--||||..||||||||||||||||.|||||||||||||||||||||||||||||||||||||||||||||||||||||||||||||||||.||||||||||||
query             0 --ATACAAAACCTAAACTTTTTCTGAAACATGATACATATGATGCTTAGATGCTGAAACTACCTGATTTTCATAACGAGACCGCTGAAAAAGTTTTGAGGTTTTCAATATTCAACTTTTT

target          118 TGATGAAAAAGTAGAGATTTTCGCACAAAATGTTGAATTTTAAAAATCTCAAAACTTTTTCAGCGGTCTCGTTATGAAAATCAGGTAGTTTCAGCATCTAAGCAGCATATTTATCATGTT
                120 ||.|||||||||||||||||||||||||||||||||||||||||||||||||||||||||||||||||||||||||||||||||||||||||||||||||||||||||||||||||||||
query           118 TGGTGAAAAAGTAGAGATTTTCGCACAAAATGTTGAATTTTAAAAATCTCAAAACTTTTTCAGCGGTCTCGTTATGAAAATCAGGTAGTTTCAGCATCTAAGCAGCATATTTATCATGTT

target          238 TGGAAAAAAGTTTAGGTTTAGTATT 263
                240 ||||||||||||||||||||||||| 265
query           238 TGGAAAAAAGTTTAGGTTTAGTATT 263
```

b

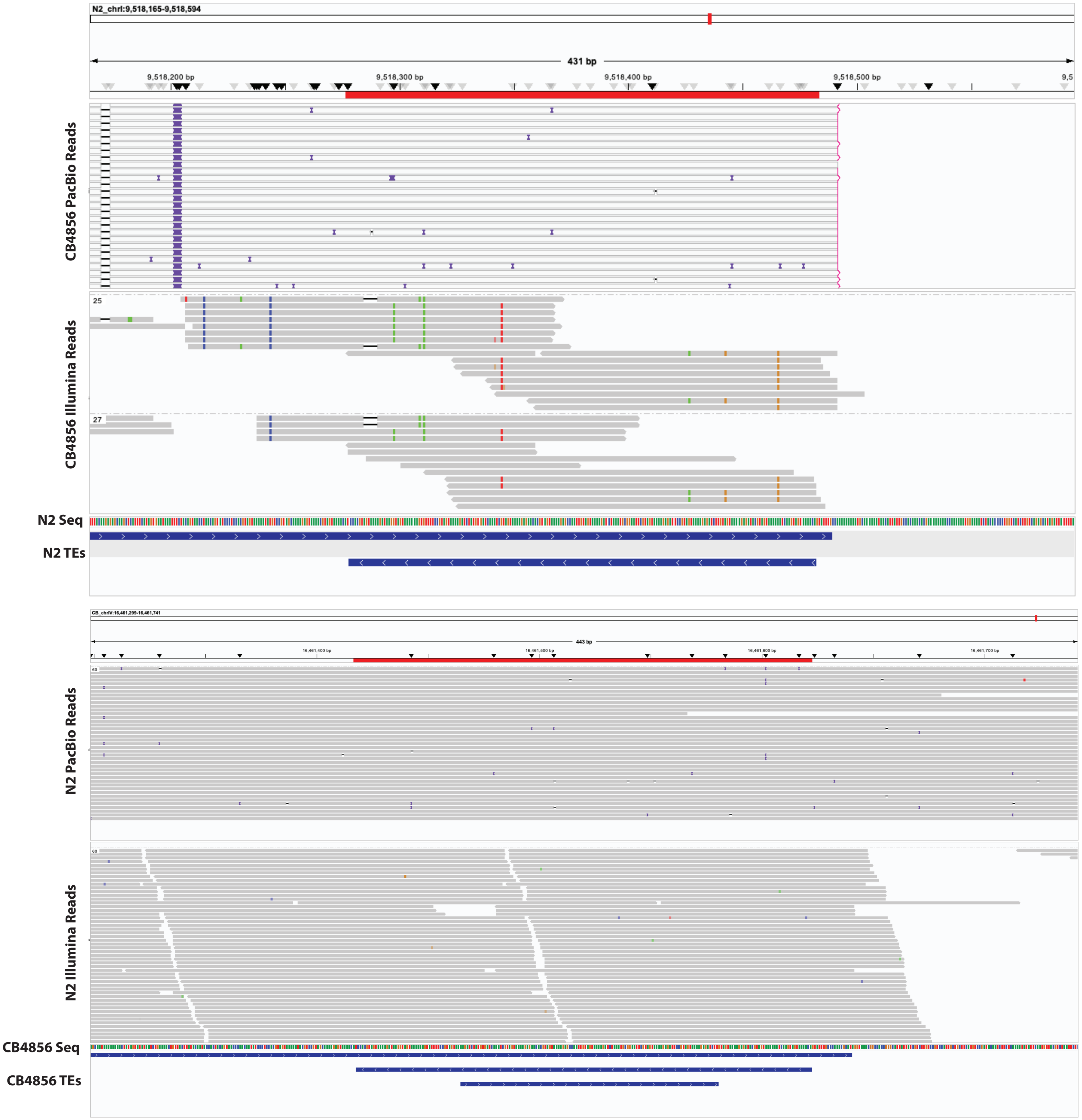


```
target          0 ----------CACGTAGTAAAAATATGTCCGTAGCAAAAGAGTAAAACGTAGTAAAACATCCACGTAGTAAAAGATAAGGTAGTAAAAAAGTGTTTTCCGTAGTAAAAGATCTATGTGAT
                0 ----------|||||||||||||||.||||||||||||||----------|||||||||||||||||||||||||||||||||||||||||||.|||||||||||||||||||||||||||
query           0 AATATCCCTCCACGTAGTAAAAATACGTCCGTAGCAAAAG----------AGTAAAACATCCACGTAGTAAAAGATAAGGTAGTAAAAAAGTATTTTCCGTAGTAAAAGATCTATGTGAT

target        110 CAAATCGTAGTAAAAGAGAACACGTAGTAAAAGAGCCAATTGTAGTAAAAGATCTAAATGCGGAATTCGTAGTAAAAGAGATTCCGTAGTAAAAGAGATCTTGTAGTAAAAGAGATACGT
              120 ||||||||||||||||||||||||||||||||||||||||||||||||||||||||||||||||||||||||||||||||||||||||||||||||||||||||||||||||||||||||
query         110 CAAATCGTAGTAAAAGAGAACACGTAGTAAAAGAGCCAATTGTAGTAAAAGATCTAAATGCGGAATTCGTAGTAAAAGAGATTCCGTAGTAAAAGAGATCTTGTAGTAAAAGAGATACGT

target        230 AGTAAAAAATCAAGTAGTAAAACAGTATTTGCCAAAAAAAAATGCAAA---AAA----TA----ACATTATTGCCACAAAC-ACGA 304
              240 |||||||||||||||||||||||||||||||||-----.|||.|||.|---|||----.|----.|||||||------|.|-.|-. 326
query         230 AGTAAAAAATCAAGTAGTAAAACAGTATTTGCC-----CAAACGCATAGGTAAACGGCCACCGGGCATTATT------ATCGTC-T 304
```

c

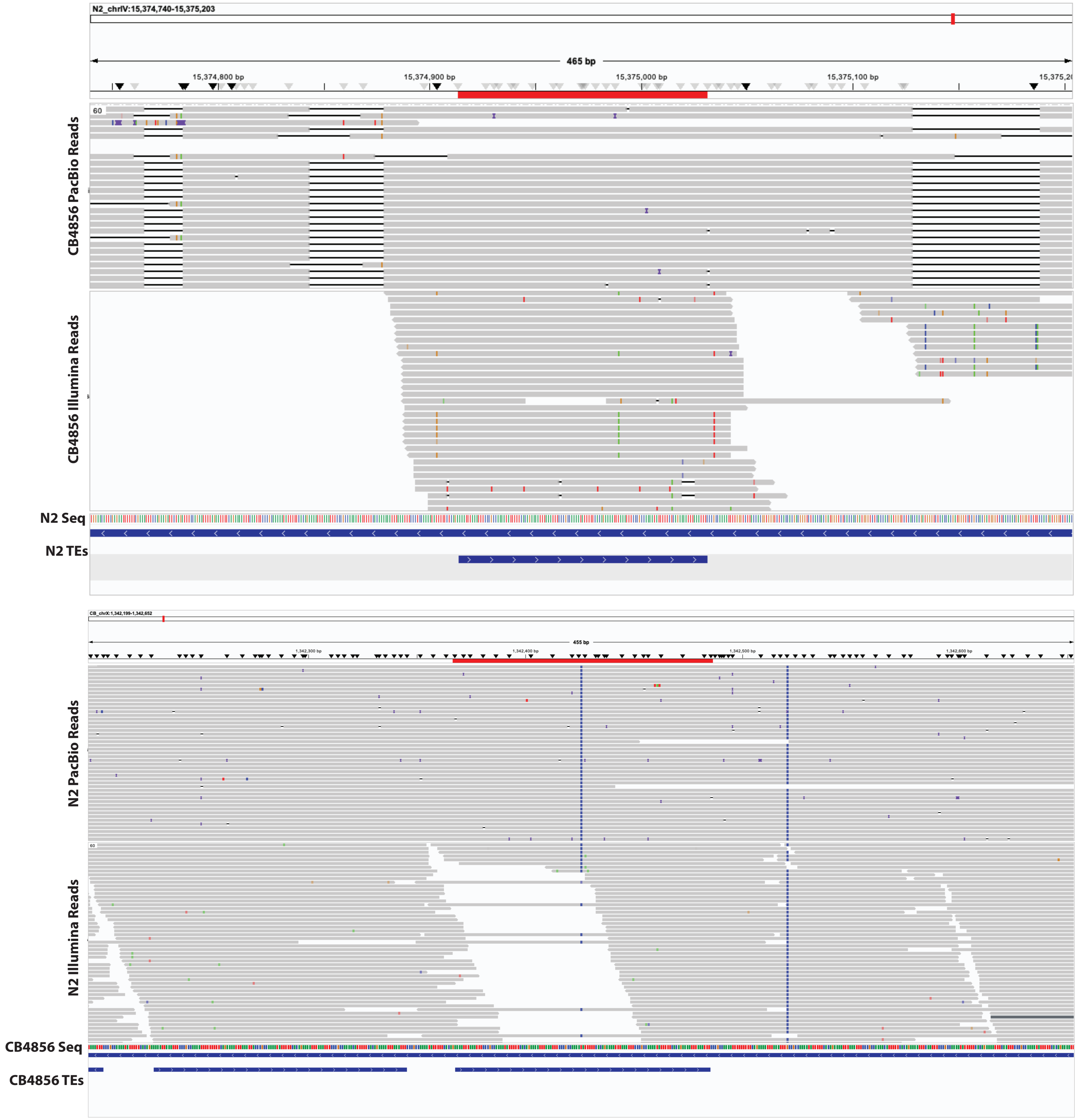


```
target           0 AATATTTTTCACAAAAAATTTAAATTTCCCTCCAAAATATTTTTCTCAGAAAATTTAAATTTCCCGCCAAAATATTTTTCACAGAAAATTTAAATTTCCCGCCAAAATATTTTTCACAGA
                 0 ||.||||||||||.||||||||||||||||.|||||||||||||||.|||||||||||||||||||||||||||||||||||||||||||||||||||||||||||||||||||||||||
query            0 AACATTTTTCACAGAAAATTTAAATTTCCCGCCAAAATATTTTTCACAGAAAATTTAAATTTCCCGCCAAAATATTTTTCACAGAAAATTTAAATTTCCCGCCAAAATATTTTTCACAGA

target         120 AAATTTAAATTTCCCGCCAAAATATTTTTCTCAGAAAATTTAAATTTCCCGCCAAAA-TTTTGGGTC-------TTACCACGGTGGGTCTCACCACGACGGGTCT 217
               120 ||||||||||||||||||||||||||||||.||||||||||||||||||||||||||-||||...||-------|||--|...||---|-|.|||--|....|.| 225
query          120 AAATTTAAATTTCCCGCCAAAATATTTTTCACAGAAAATTTAAATTTCCCGCCAAAATTTTTTTTTCAGAAAATTTA--AATTTG---C-CGCCA--AAATATTT 217
```

d

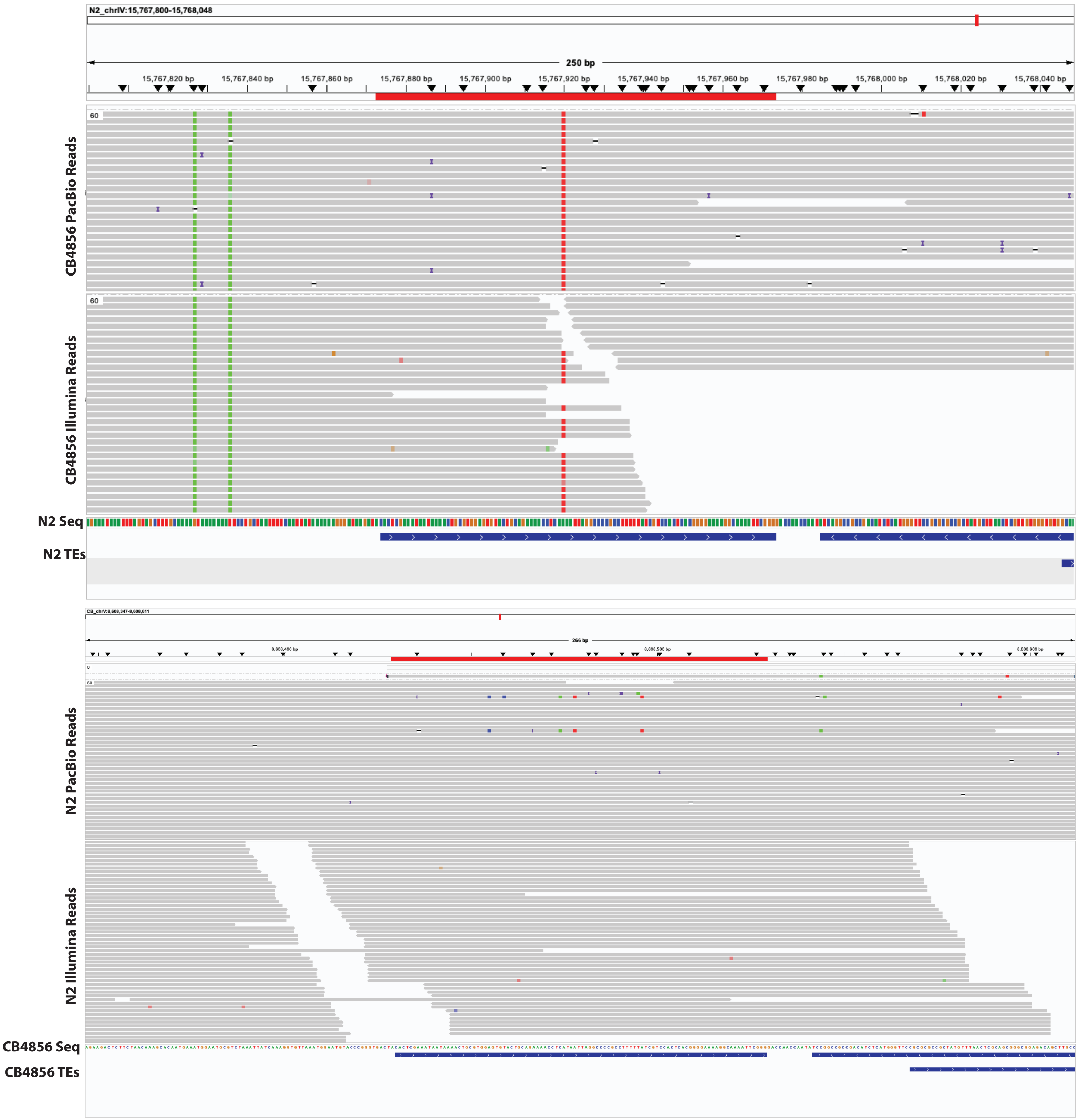


```
target            0 AAGTAAAAAAATTCCTGCT-AATTTTCAA---T-TATAAA---AA---A---GGGATAAGA-TACACTCGAAATAATAAAACTGCGTGGAGTGTACTGCAGAAAACCTCAAAATTAGGCC
                  0 ----------||.|.|-||-||||.||||---|-|-||||---||---|---|||-|--||-||||||||||||||||||||||||||||||||||||||||||||||||.|||||||||
query             0 ----------ATGCGT-CTAAATTATCAAAGGTGT-TAAATGGAATGTACCCGGG-T--GACTACACTCGAAATAATAAAACTGCGTGGAGTGTACTGCAGAAAACCTCATAATTAGGCC

target          105 CCGCCTTTTTATCGTCCACTCACGGGGAAAAGGCAAAATTCGGGGACCAACCAATATCAGGCCGCCGACATCTCATGGGTTCCGCGCGCCGCTA 199
                120 ||||||||||||||||||||||||||||||||||||||||||||||||||||||||||.||||||||||||||||||||||||||||||||||| 214
query           105 CCGCCTTTTTATCGTCCACTCACGGGGAAAAGGCAAAATTCGGGGACCAACCAATATCCGGCCGCCGACATCTCATGGGTTCCGCGCGCCGCTA 199
```

e

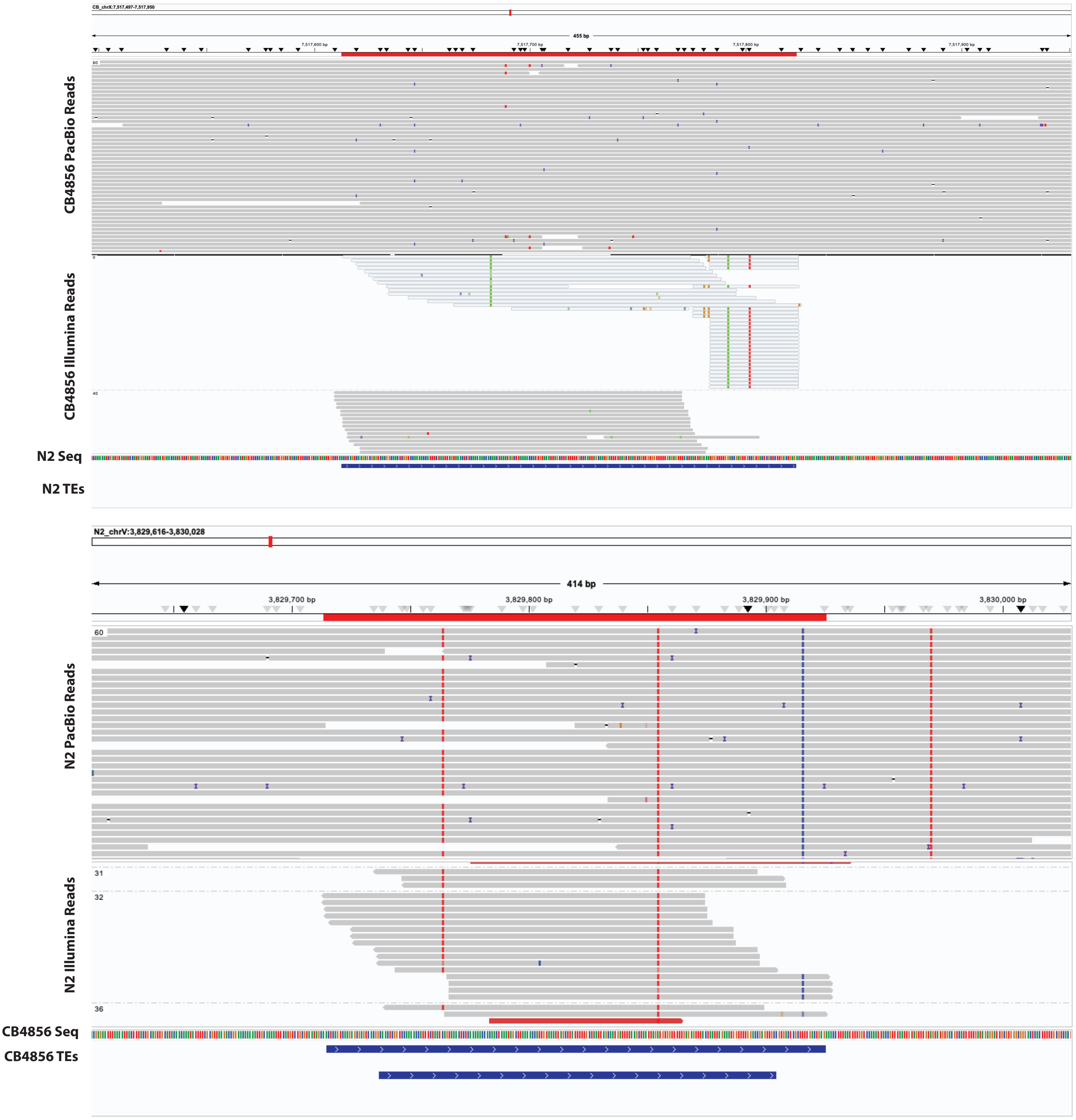


```
target            0 C----GTT---AAGACCGGAACAAAAATTGCA-AAATTTCATGTCTGC-GTAATAG-C-AACTCAGTCAAGAGCGAGTCACGGCAACTCGGTCCAAAACCATTTCTAATCAGTAAACTCT
                  0 |----|||---|||.||--|.|-----|.|.|-|.|||||----||||-.||||.|-|-||||||||||||||||||||||||||||||||||||||||||||||||||.||||||||||
query             0 CCATAGTTTCCAAGCCC--AGC-----TGGTATACATTTC----CTGCAATAATTGTCTAACTCAGTCAAGAGCGAGTCACGGCAACTCGGTCCAAAACCATTTCTAATTAGTAAACTCT

target          109 CAAAAACCACAACTAAATAGCTTAAAACCATTGTAAATTAGCTTATTTTTGCTAATTAGCAATGATTTTAAGCTAATTAGATGTGGTTTTTGAGAGTTTACTAATTAGAAATGGTTTTGG
                120 ||||||||||||||||||||||||||||||||||||||||||||||||||||||||||||||||||||||||||||||||.|||||||||||||||||||||||||||||||||||||||
query           109 CAAAAACCACAACTAAATAGCTTAAAACCATTGTAAATTAGCTTATTTTTGCTAATTAGCAATGATTTTAAGCTAATTAGTTGTGGTTTTTGAGAGTTTACTAATTAGAAATGGTTTTGG

target          229 ACCGAGTTGCCGTGACTCGCTTTTGACTGAGAAACCTTTAAT----TTTGGTCCAA--ATTACCGTTTA-ATTGT---AGTTTGTAGTATG 310
                240 ||||||||||||||||||||.|||||||||||----|||||----..|..|-|||--|.||--|-..|-|||||---||-.||-||||.. 331
query           229 ACCGAGTTGCCGTGACTCGCTCTTGACTGAGAA----TTAATAAACAATAAT-CAAGCAATA--G-GAAGATTGTTGGAG-GTG-AGTACC 310
```

f

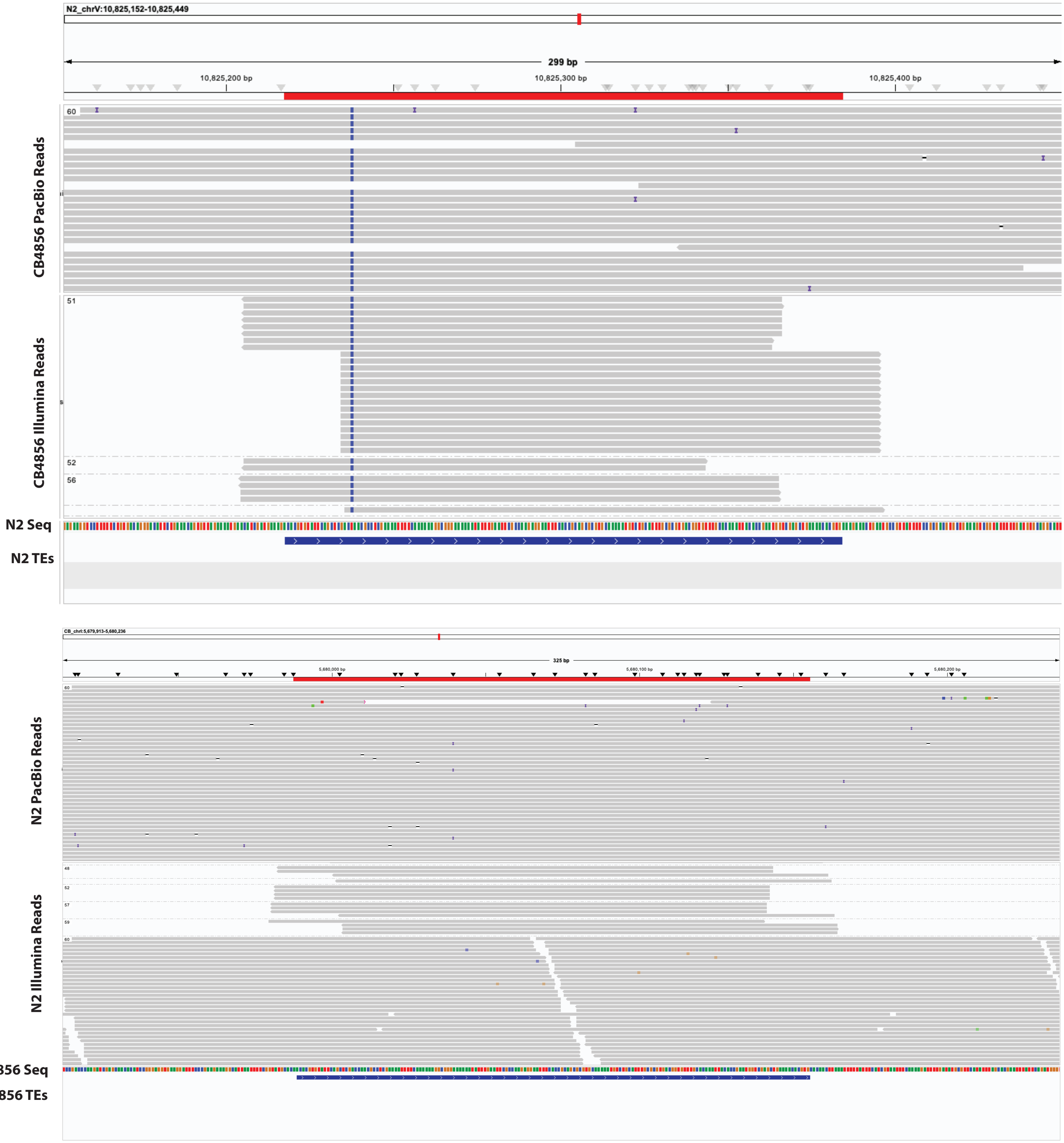


```
target            0 GTGACAGG-GA--CATT--CTGAA-ACAGTG--TTAGAAAATGAACATCG--ATG-AAAGACACGTGTATTCAGCTCGACTAACGCCTCGAAAATTTTCAAAAAAGGCGGGAAAAAAATAT
                  0 |.|--|||-||--.|||--|.|||-|.||-|--||----|||---..|||--|||-|.|-|||||||||||||||||||.|||||||||||||||||||||||||||||||||||||
query             0 GGG--AGGTGAAGGATTTCCCGAAGAAAG-GCATT----AAT---TTTCGGTATGCACA-ACACGTGTATTCAGCTCGACCAACGCCTCGAAAATTTTCAAAAAAGGCGGGAAAAAAATAT

target          109 TTGAATTCGCCAAGAGGAATTTCACCGCAGCGCGTGCAAAAATGTCTGCATTTGCGCGTGACGGTGTTTGCACAAATTACACCGAATGGTCGAGCTGAAAACACGTGAAAGAAAC-TGAA
                120 |||||||||||||||||||||||||||||||||||||||||||||||||||||||||||||||||||||||||||||||||||||||||||||||||||||.|--.|||-|.||
query           109 TTGAATTCGCCAAGAGGAATTTCACCGCAGCGCGTGCAAAAATGTCTGCATTTGCGCGTGACGGTGTTTGCACAAATTACACCGAATGGTCGAGCTGAAAACACGTGCA--CAACTTAAA

target          228 TGCCGTTATTTTCCGCG-ACGTGCTGAAAG---TCGGTAT-TT 266
                240 |--..|||-||||..||-|..|.|-.||||---|.|.|||-.. 283
query           227 T--TTTTA-TTTCATCGTAATTTC-AAAAGATTTTGATATGCG 266
```

g

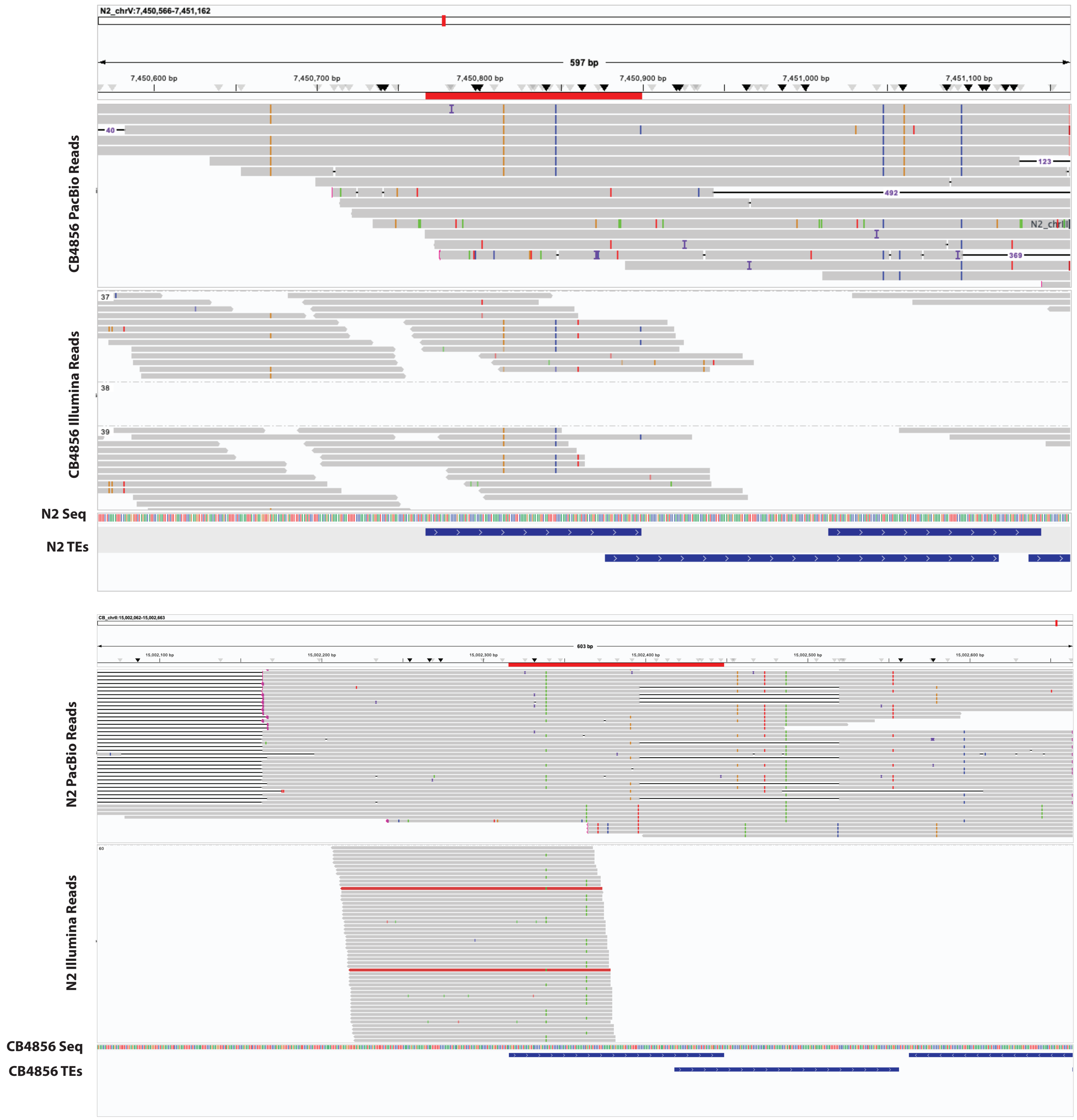


```
target          0 AATTTTTCCCAAACTACAGTAATCCTACAGTAACCCTACAGTACCTCTACAGTACTACTACAGTACCCCGACCATATCCCACCACTAACCTAAAACCAATATCCCTTCAAAAGACGAAAA
                0 |||||||||||||||||||||||||||||||||||||||||||||||||||||||||||||||||||||||||||||||||||||||||||||||||.||||||||||||||||||||||
query           0 AATTTTTCCCAAACTACAGTAATCCTACAGTAACCCTACAGTACCTCTACAGTACTACTACAGTACCCCGACCATATCCCACCACTAACCTAAAACCGATATCCCTTCAAAAGACGAAAA

target        120 GTCAATTTTTCCCAAACTACAGTAATCCTACAGTAACCCTACAGTACCTCTACAGTACTACTACAGTACCCCGACCATATCCCACCACTAACCTAAAACCAATATCCCTTCA 232
              120 |||||||||.||||||||||||||||||||||||||||||||||||||||||||||||||||||||||||||||||||||||||||||||||.||||||||||| 232
query         120 GTCAATTTTCCCCAAACTACAGTAATCCTACAGTAACCCTACAGTACCTCTACAGTACTACTACAGTACCCCGACCATATCCCACCACTAACCTAAAACCGATATCCCTTCA 232
```

h

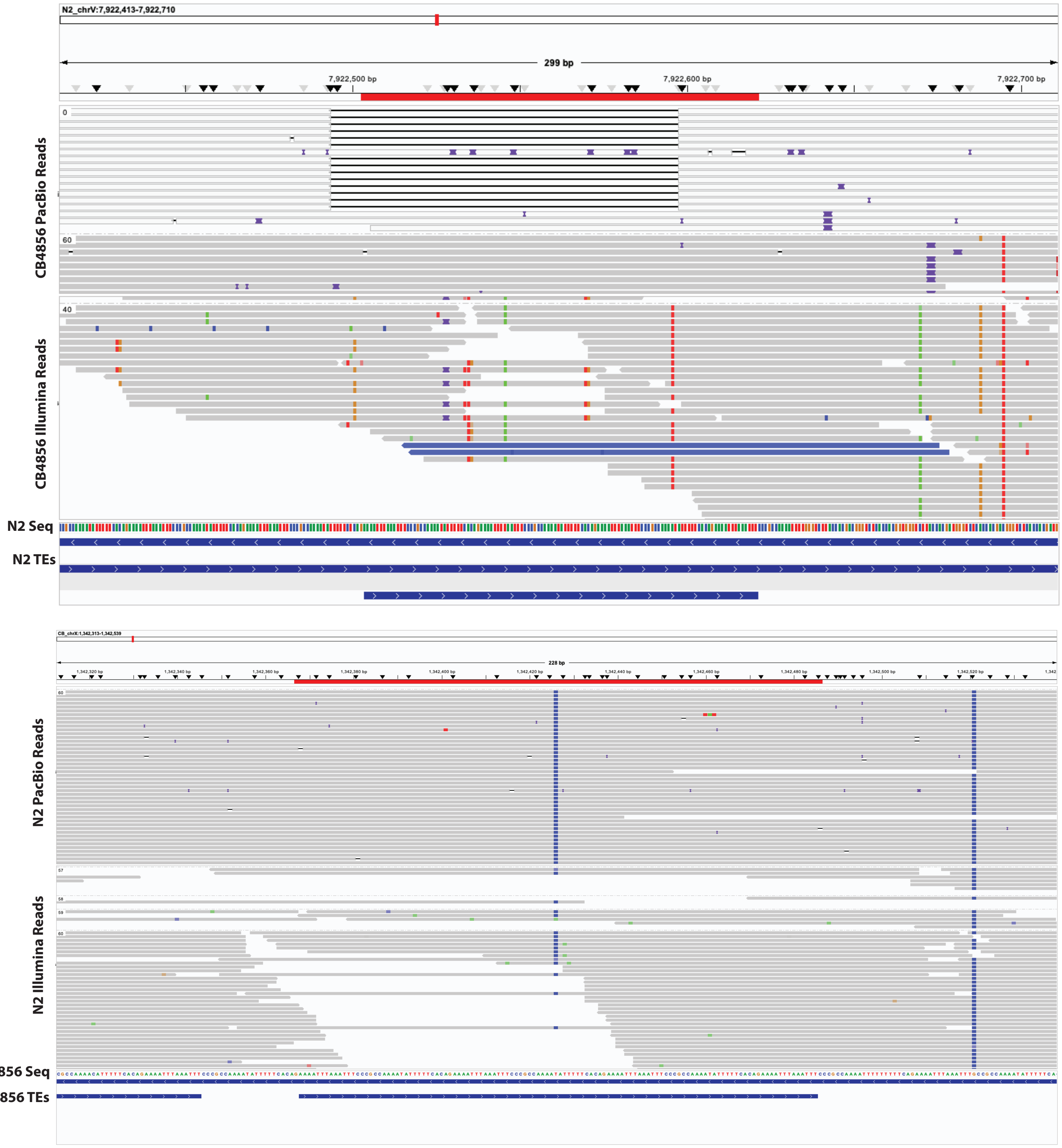


```
target             0 AATATTTTTCACAGAAAATTTAAATTTCCCGCCAAAATATTTTTCTCAGAAAATTTAAATTTCCCGCCAAAATATTTTTCACAGAAAATTTTAATTTCCCGCCAAAATATTTTTCACAGA
                   0 ||.||||||||||||||||||||||||||||||||||||||||||.|||||||||||||||||||||||||||||||||||||||||||||.||||||||||||||||||||||||||||
query              0 AACATTTTTCACAGAAAATTTAAATTTCCCGCCAAAATATTTTTCACAGAAAATTTAAATTTCCCGCCAAAATATTTTTCACAGAAAATTTAAATTTCCCGCCAAAATATTTTTCACAGA

target           120 AAATTTAAATTTCCCGCCAAAATATTTTTCACAGAAAATTTAAATTTCCCGCCAAAATTTTGGGTCTTACCACGACGGGT--------CTCACCACGGTGGGTCT 217
                 120 |||||||||||||||||||||||||||||||||||||||||||||||||||||||||||||---|-||..||-||....|--------|-|.|||...|--.|.| 225
query            120 AAATTTAAATTTCCCGCCAAAATATTTTTCACAGAAAATTTAAATTTCCCGCCAAAATTTT---T-TTTTCA-GAAAATTTAAATTTGC-CGCCAAAAT--ATTT 217
```

i

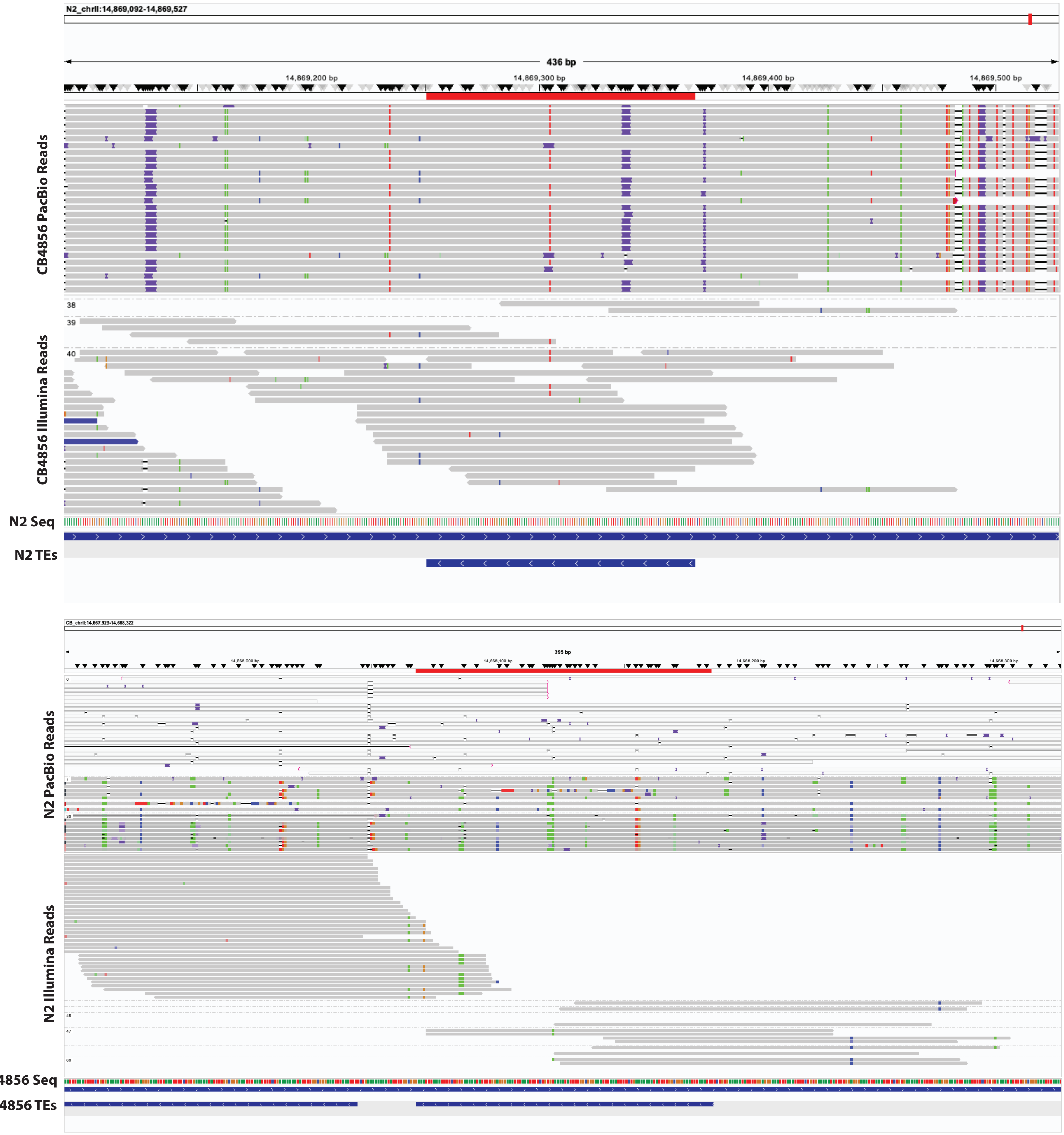


```
target            0 AAATATTTTGGAGGGAAATTTAAATTTTCTG-TGAAAAATATTTTGGAGGGAAATTTAAATTTTCTGTGAAAAATATTTTGGAGGGAAATTTAAATTTTCTGTGAAAAATATTTTGGCGG
                  0 -||||||||||.|||||||||||||||||||-..|||||||||||||.||||||||||||||||||||||||||||||||||||||||||||||||||||||||.|||||||||||||||
query             0 -AATATTTTGGCGGGAAATTTAAATTTTCTGAAAAAAAAATATTTTGGCGGGAAATTTAAATTTTCTGTGAAAAATATTTTGGAGGGAAATTTAAATTTTCTGTGTAAAATATTTTGGCGG

target          119 GAAATTTAAATTTTCTGAAAAAAAATATTTTGGCGGGAAATTTAAATTTTCTGAAAAAAAATATTTTGGCGGGAAATTTAAATTTTCTGTGAAAAATA-T 217
                120 |||||||||||||||||||||||||||||||||||||||||||||||||||||-..|||||||||||||.||||||||||||||||||||||||||||-| 219
query           119 GAAATTTAAATTTTCTGAAAAAAAATATTTTGGCGGGAAATTTAAATTTTCTG-TGAAAAATATTTTGGAGGGAAATTTAAATTTTCTGTGAAAAATATT 217
```

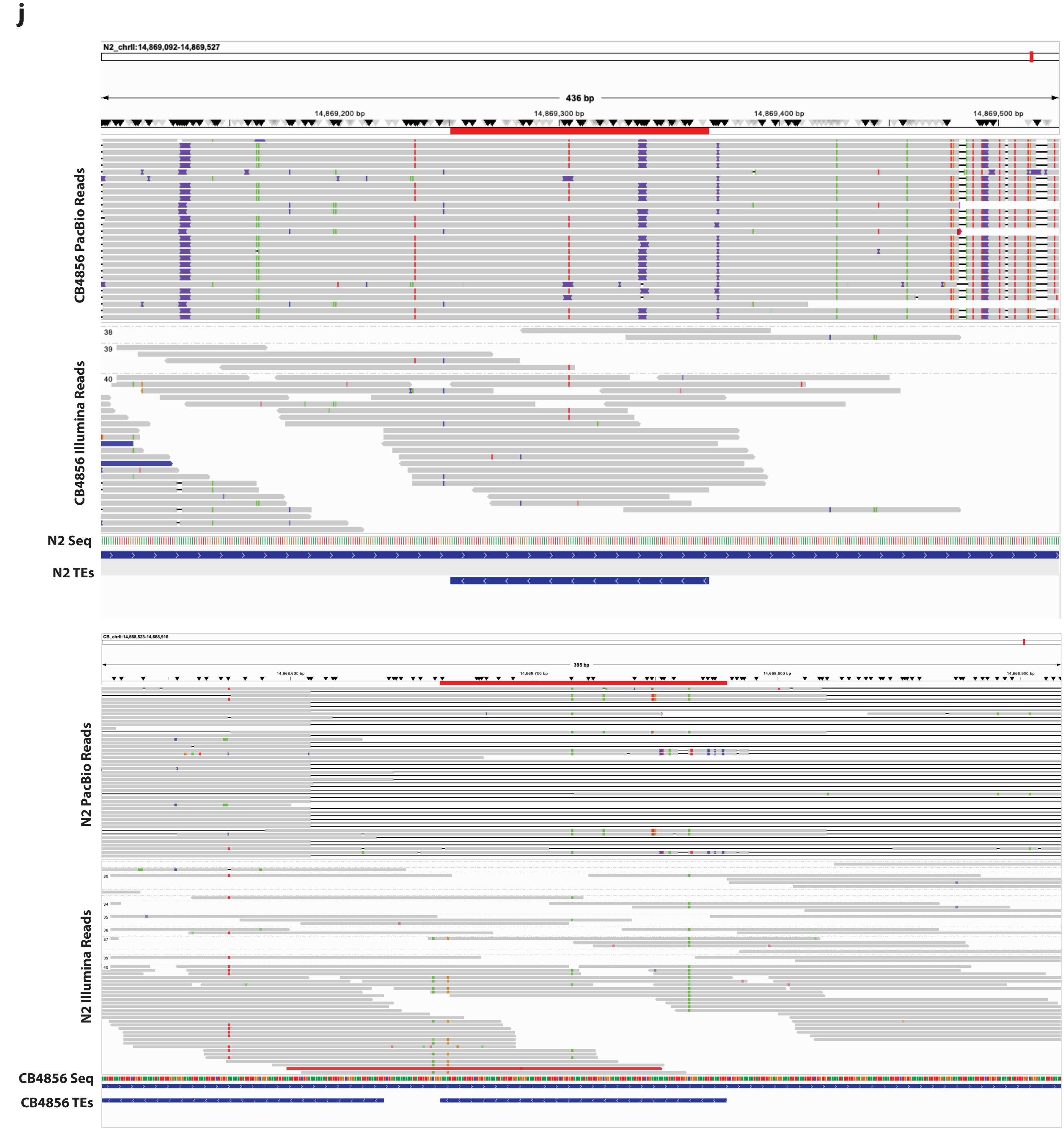


```
target               0 AAATATTTTGGAGGGAAATTTAAATTTTCTG-TGAAAAATATTTTGGAGGGAAATTTAAATTTTCTGTGAAAAATATTTTGGAGGGAAATTTAAATTTTCTGTGAAAAATATTTTGGCGG
                     0 -||||||||||.||||||||||||||||||-..|||||||||||||.||||||||||||||||||||||||||||||||||||||||||||||||||||||||.|||||||||||||||
query                0 -AATATTTTGGCGGGAAATTTAAATTTTCTGAAAAAAAAATATTTTGGCGGGAAATTTAAATTTTCTGTGAAAAATATTTTGGAGGGAAATTTAAATTTTCTGTGTAAAATATTTTGGCGG

target             119 GAAATTTAAATTTTCTGAAAAAAAAATATTTTGGCGGGAAATTTAAATTTTCTGAAAAAAAAATATTTTGGCGGGAAATTTAAATTTTCTGTGAAAAATA-T 217
                   120 ||||||||||||||||||||||||||||||||||||||||||||||||||||||-..|||||||||||||.|||||||||||||||||||||.||||||-| 219
query              119 GAAATTTAAATTTTCTGAAAAAAAAATATTTTGGCGGGAAATTTAAATTTTCTG-TGAAAAATATTTTGGAGGGAAATTTAAATTTTCTGTGTAAAATATT 217
```

k

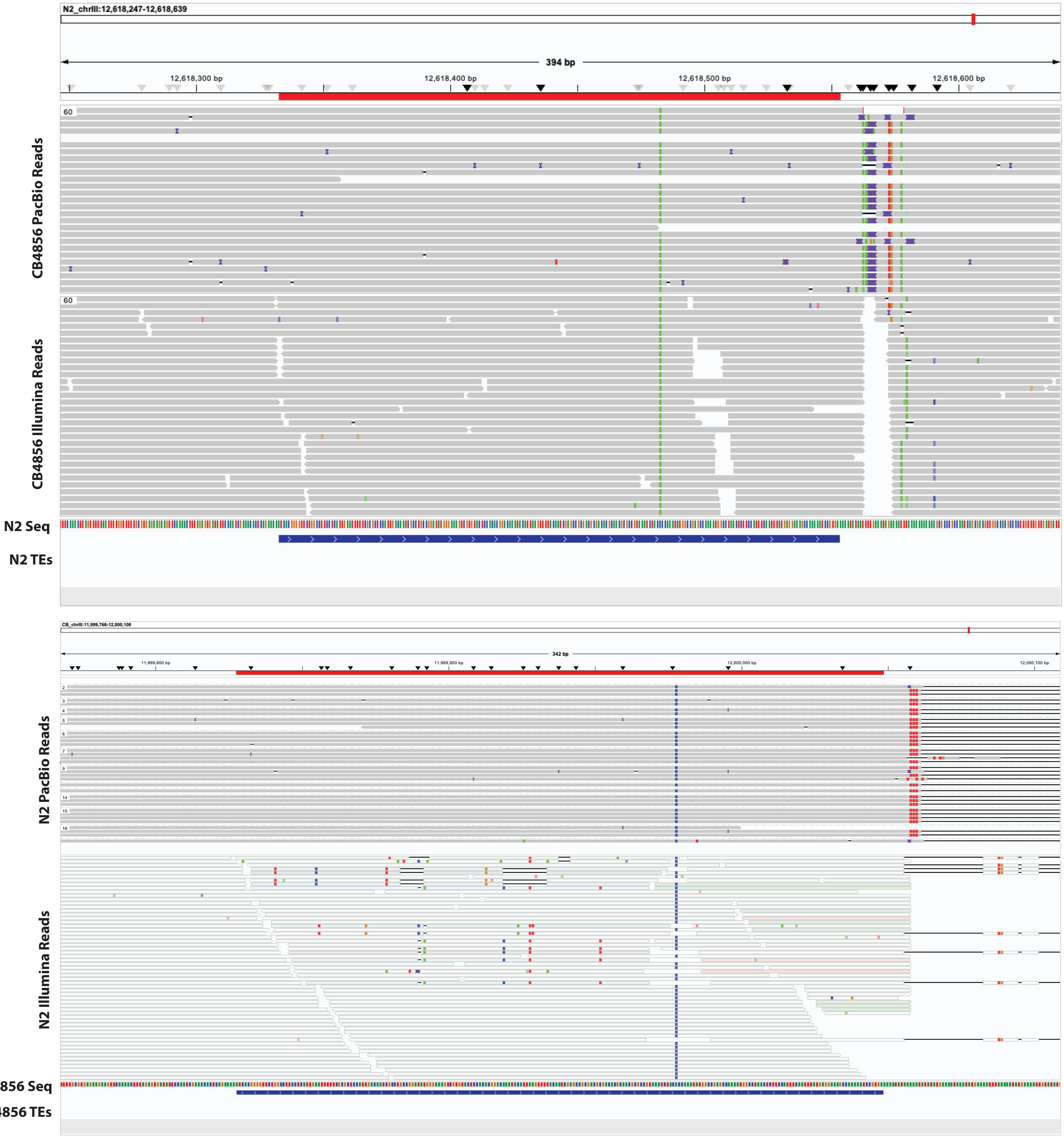


```
target           0 AAGAAGTTCAAAACTTTAGAGTGATATTCCAATCGAAACACTCAGAAAAATCACGGGGTTCTGGCCTTCCTCATTGAATTTTTCGCGCTCCATTGACAATCGCCTGCCGGATATGGGAAA
                 0 ||||||||||||||||||||||||||||||||||||||||||||||||||||||||||||||||||||||||||||||||||||||||||||||||||||||||||||||||||||||||
query            0 AAGAAGTTCAAAACTTTAGAGTGATATTCCAATCGAAACACTCAGAAAAATCACGGGGTTCTGGCCTTCCTCATTGAATTTTTCGCGCTCCATTGACAATCGCCTGCCGGATATGGGAAA

target         120 GTCGTGTACTCCACACGGACAAATACATTAAGTTTTACAATCGAGCCGCGACGCGACACGCAACGCGCCGTAAATCTACCCCAAATATGGCCGAGCCAAAATGGCCTAACTCTTCCATTT
               120 |||||||||||||||||||||||||||||||||||||||||||||||||||||||||||||||||||||||||||||||.||||||||||||||||||||||||||||||||||||||||
query          120 GTCGTGTACTCCACACGGACAAATACATTAAGTTTTACAATCGAGCCGCGACGCGACACGCAACGCGCCGTAAATCTACACCAAATATGGCCGAGCCAAAATGGCCTAACTCTTCCATTT

target         240 CAATTTATGAGGAAAGCCAAAAATCCGTGAAAAATAT---AAT-T-T-TG-----AAATA-GAAAATTTAAAAAAAAAAAGCGCCCGCCAAT 320
               240 |||||||||||||||||||||||||||||||||||||---|||-|-|-||-----|||||-|||||||||||||.|---------.|--||- 332
query          240 CAATTTATGAGGAAAGCCAAAAATCCGTGAAAAATATAAAAATATATATGAAAATAAATATGAAAATTTAAAAATA---------TG--AA- 320
```

I

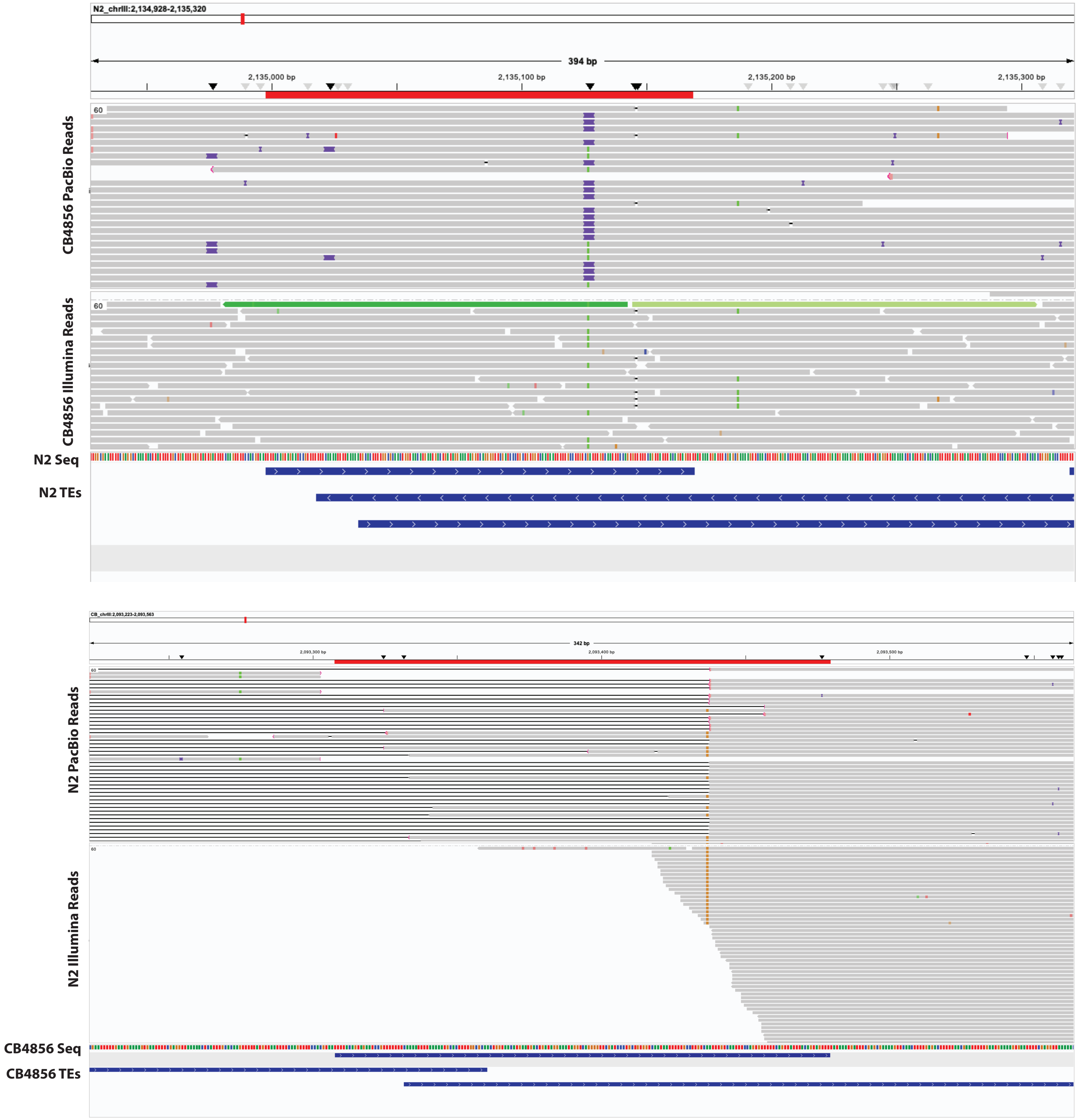


```
target            0 ATACTTATTTTTGCTGA--TTTGTGTATTGATTTTCAAGTTTTCCACGTAATTTTACAGTAGAATCCGTTTTTTTCAAAATTTTAAAAATGCACTTTTGGCGCAACTGGTAACATGAATG
                  0 |.|||.|--|||||..|--|||.|.|.||.||||||||||||||||||||||||||||||||||||||||||||||||||||||||||||||||||||||||||||||||||||||||||
query             0 AAACTCA--TTTGCACAAGTTTTTATGTTAATTTTCAAGTTTTCCACGTAATTTTACAGTAGAATCCGTTTTTTTCAAAATTTTAAAAATGCACTTTTGGCGCAACTGGTAACATGAATG

target          118 ACTGATAGGAAATAGGTCAGGGGTTCGATCCCTGCTGTGGTCAATAATTTTTGCCTTGTCGATTTGTCAATTTCCAGTGTTTTTCAGTTATTTACAGTAGTTTTATAAATGATTTCGAGC
                120 ||||||||||||||||||||||||||||||||||||||||||||||||||||||||||||.|||||||||||||||||||||||||||||||||||||||||||||||||||||||||||
query           118 ACTGATAGGAAATAGGTCAGGGGTTCGATCCCTGCTGTGGTCAATAATTTTTGCCTTGTCAATTTGTCAATTTCCAGTGTTTTTCAGTTATTTACAGTAGTTTTATAAATGATTTCGAGC

target          238 GAAAAGTTCAACTTTTGAGACTTTTTAACGAAT 271
                240 ||||||||||||||||||||||||||||||||| 273
query           238 GAAAAGTTCAACTTTTGAGACTTTTTAACGAAT 271
```

m

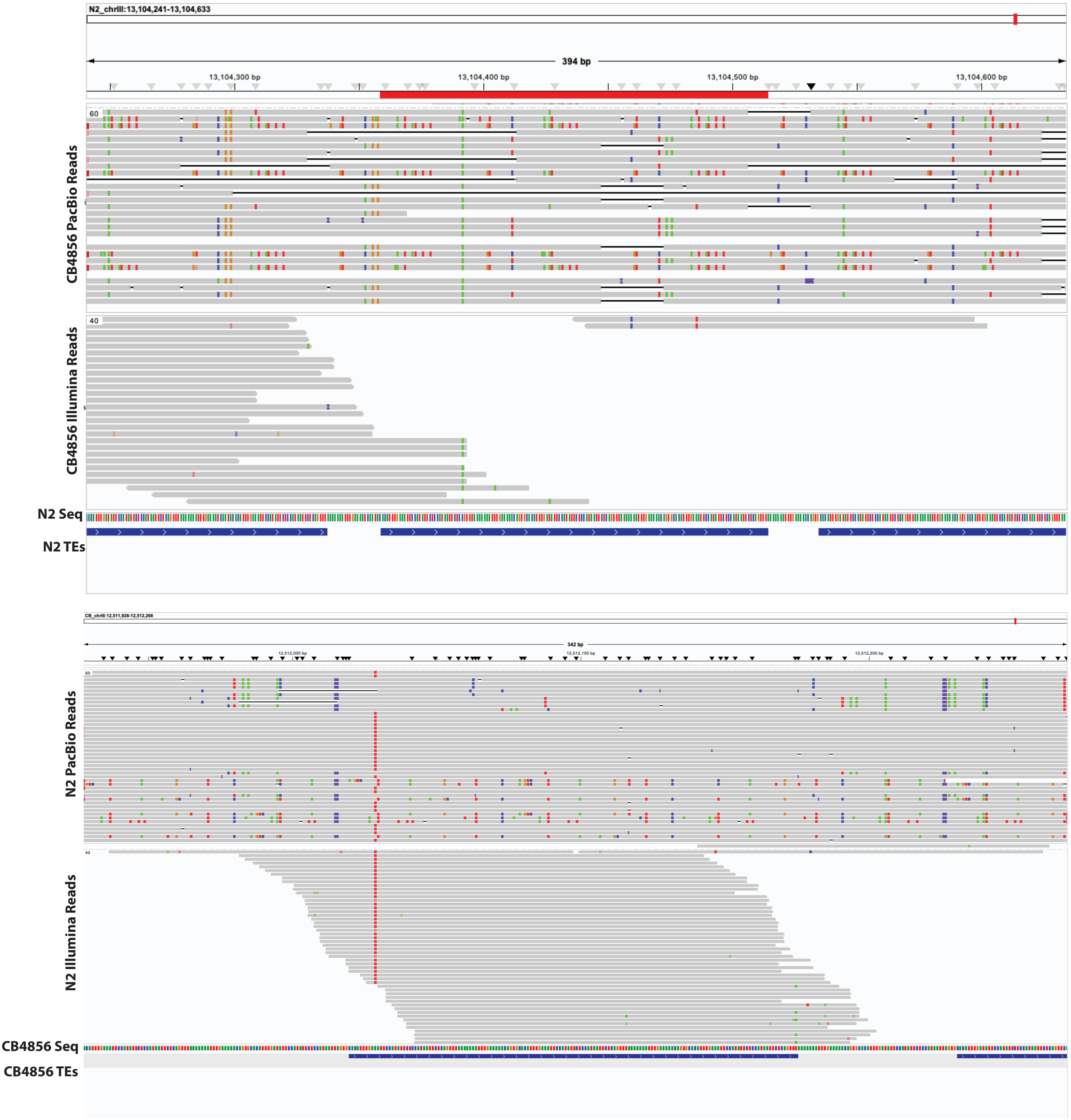


```
target            0 ATTCCATATTCTCCATAATTCTCAGTTTAAAAAAATTTGAAAATCAATACACATTTGAAATTCCATATTCTCCATAATTCTCCGTTTAAAAAAATTTGAAAAACAGTGCACATTTGATAT
                  0 ||||||||||||||||||||||||||||||||||||||||||||||.|.|||||||||||||||||||||||||||||||||.||||||||||||||||||||||||||||||||||.||
query             0 ATTCCATATTCTCCATAATTCTCAGTTTAAAAAAATTTGAAAATCAGTGCACATTTGAAATTCCATATTCTCCATAATTCTCAGTTTAAAAAAATTTGAAAAACAGTGCACATTTGAAAT

target          120 TCCATATTCTCCATAATTCTCAGTTTAAAAAAATTTGAAAAACAGTGCACATTTGAAATTCCATATTCTCCATAATTCTCAGTTTAAAAAAATTTGAAAAACAGTGCACATTTGATATTC
                120 |||||||||||||||||||||||||||||||||||||||||||||||||||||||||||||||||||||||||||||||||||||||||||||||||||||||||||||||||||.||||
query           120 TCCATATTCTCCATAATTCTCAGTTTAAAAAAATTTGAAAAACAGTGCACATTTGAAATTCCATATTCTCCATAATTCTCAGTTTAAAAAAATTTGAAAAACAGTGCACATTTGAAATTC

target          240 CATATTCTCCATAAT 255
                240 ||||||||||||||| 255
query           240 CATATTCTCCATAAT 255
```

n

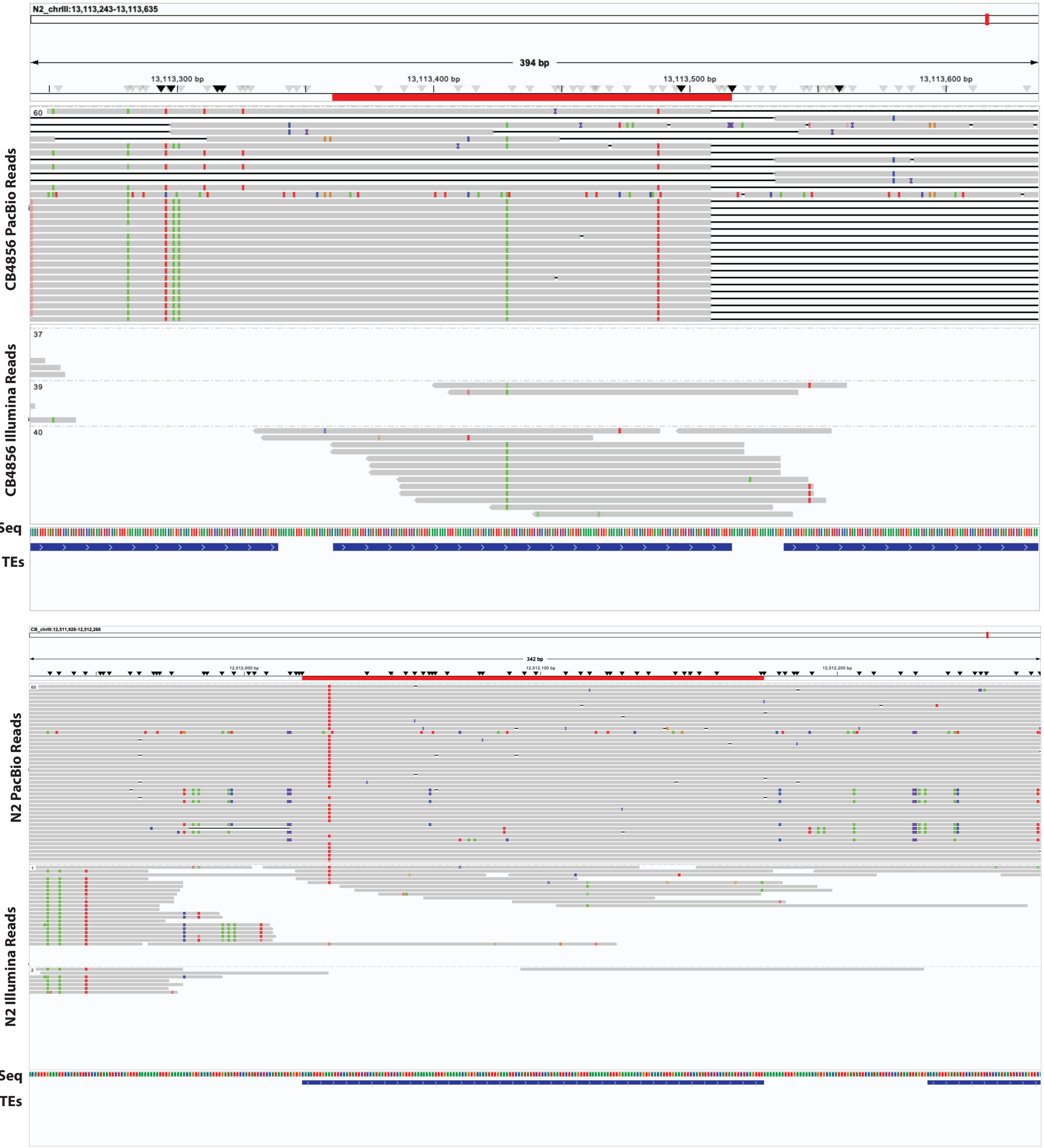


```
target            0 ATT-------C----CATATTCTCCATAATTCTCAGTTTAAAAAAATT-T-GAAAATCAATACACATTTGAAATTCCATATTCTCCATAATTCTCAGTTTAAAAAAATTTGAAAAACAGT
                  0 |||-------|----||.|||-|-.||-||||-||---|------|||-|-.|.|||.|.|.||||||||||||||||||||||||||||||||||||||||||||||||||||||||||
query             0 ATTTGAAAAACAGTGCACATT-T-GAT-ATTC-CA---T------ATTCTCCATAATTAGTGCACATTTGAAATTCCATATTCTCCATAATTCTCAGTTTAAAAAAATTTGAAAAACAGT


target          107 GCACATTTGATATTCCATATTCTCCATAATTCTCAGTTTAAAAAAATTTGAAAAACAGTGCACATTTGAAATTCCATATTCTCCATAATTCTCAGTTTAAAACAATTTGAAAAACAGTGC
                120 ||||||||||.|||||||||||||||||||||||||||||||||||||||||||||||||||||||||||||||||||||||||||||||||||.|||||||||||||||||
query           107 GCACATTTGAAATTCCATATTCTCCATAATTCTCAGTTTAAAAAAATTTGAAAAACAGTGCACATTTGAAATTCCATATTCTCCATAATTCTCAGTTTAAAAAAATTTGAAAAACAGTGC


target          227 ACATTTGAAATTCCATATTCTCCATAAT 255
                240 ||||||||.||||||||||||||||||| 268
query           227 ACATTTGATATTCCATATTCTCCATAAT 255
```

o

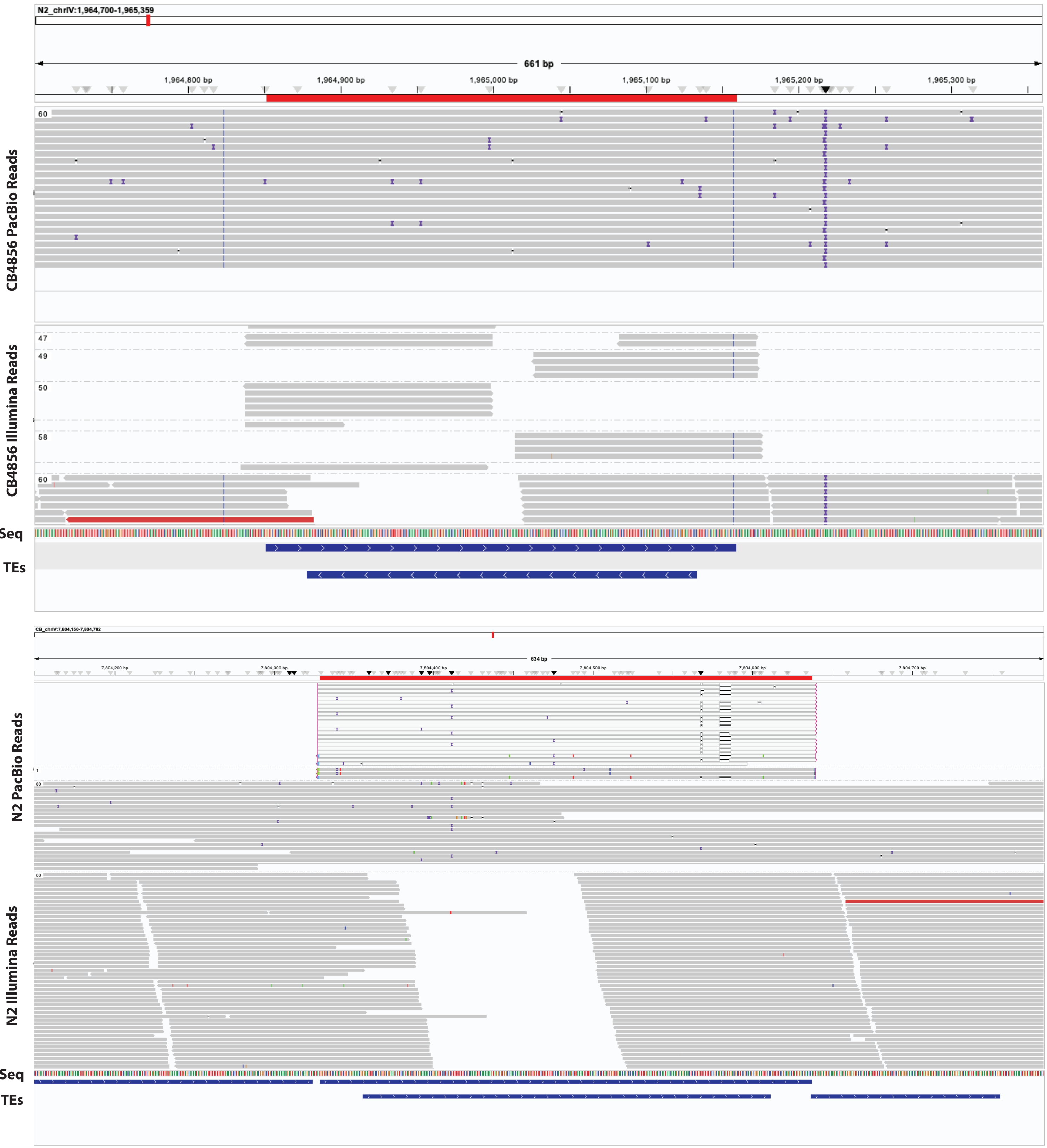


```
target            0 A--TAATTC---A-GTTTTTG-AA-AAAATTGATTTTTCGAAA-ACAATCATTGTTCATGGTGCGTCCATAGTCTGCGGAAAACGGGAATTTCCCGCGTGCGGAAGCGTCCATAGTCTGC
                  0 |--|||--|---|-|....||-||-||||..||.|-----|||-.|||-.|.|.||-||||||||||||||||||||||||||||||||||||||||||||||||||||||||||||||
query             0 AATTAA--CGAGACGGGCCTGCAATAAAAAAGAGT-----AAACGCAA-GACTATT-ATGGTGCGTCCATAGTCTGCGGAAAACGGGAATTTCCCGCGTGCGGAAGCGTCCATAGTCTGC

target          111 GGAAAAACCGGAGTTCCTCGCTTTTTTTTCCTCAAAATTCAAAAAAGTAGGCGTGGCCAACCAATCAGCTGTTGTTTCTTGTTTTCCCATTGCTCAGCTTAAAATTTTACAGCCTCTAAT
                120 ||||||||||||||||||||||||||||||||||||||||||||||||||||||||||||||||||||||||||||||||||||||||||||||||||||||||||||||||||||||||
query           111 GGAAAAACCGGAGTTCCTCGCTTTTTTTTCCTCAAAATTCAAAAAAGTAGGCGTGGCCAACCAATCAGCTGTTGTTTCTTGTTTTCCCATTGCTCAGCTTAAAATTTTACAGCCTCTAAT

target          231 TGGTTGAACACGCCCACTATTTTGAAATTGACCAATAACAAAGCGAGAAACTCCTTGTTTTTTTCCGCAGACTATGGACGCTTCCGCACGCGGGAATTTCCCGTTTTCCGCAGACTATGG
                240 ||||||||||||||||||||||||||||||||||||||||||||||||||||||||||||||||||||||||||||||||||||||||||||||||||||||||||||||||||||||||
query           231 TGGTTGAACACGCCCACTATTTTGAAATTGACCAATAACAAAGCGAGAAACTCCTTGTTTTTTTCCGCAGACTATGGACGCTTCCGCACGCGGGAATTTCCCGTTTTCCGCAGACTATGG

target          351 ACGCATCATCAGAAAATTTCCACGAAATTTCGAAAAAAAATCGGTG--AT-----TTTTCAGAA--- 408
                360 |||||.|||.|||.|--.|.||.||-----|.||.|...|---|||--||-----|||||.|||--- 427
query           351 ACGCACCATTAGAGA--GTGCAAGA-----CTAATAGGGA---GTGCCATACTAATTTTCGGAAGGT 408
```

p

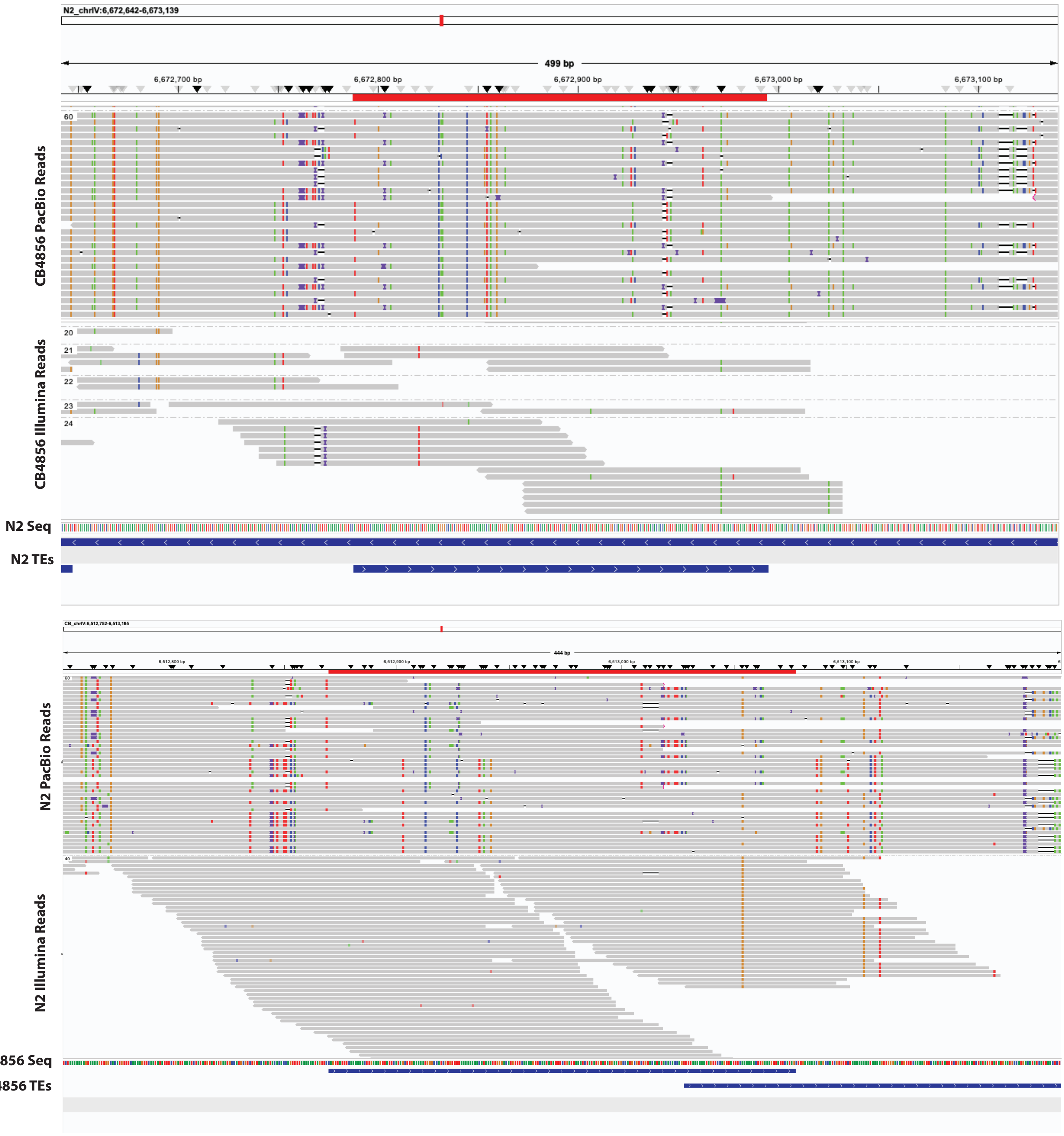


```
target            0 TGAATGCCATGCGTCTGTACATATACACACCAGTTTTAAAAAATAGTTCCAGTTATTTCAAAAAACTTGAAAGTTATTCATAGCTTGAAAAATGGGCCATCGTGTTTTAAAAAAAAACATA
                  0 ||||||||||||||||||||||||||||||||||||||||||||||||||||||||||||||||||||||||||||||||||||||||||||||||||||||||||||||||||||||||
query             0 TGAATGCCATGCGTCTGTACATATACACACCAGTTTTAAAAAATAGTTCCAGTTATTTCAAAAAACTTGAAAGTTATTCATAGCTTGAAAAATGGGCCATCGTGTTTTAAAAAAAAACATA

target          120 AATTGGGACATGTTTCAAAAGTTCAATATATTTCTGATTAAATCTATATGTATATGAATGCCATGCGTCTGTACATATACACACCAGTTTTAAAAAATAGTTCCAGTTATTTCGAAAAAC
                120 |||||||||||||||||||||||||||||||||||||||||||||||||||||||||||||||||||||||||||||||||||||||||||||||||||||||||||||||||.||||||
query           120 AATTGGGACATGTTTCAAAAGTTCAATATATTTCTGATTAAATCTATATGTATATGAATGCCATGCGTCTGTACATATACACACCAGTTTTAAAAAATAGTTCCAGTTATTTCAAAAAAC

target          240 TTGAAAGTTATTCATAGCTTGAAAAACGAGCCATCGTGTTCTAAAAAGAA-TAAATGTTGGGACATGT 307
                240 ||||||||||||||||.|||||||||||.||||||||..||||||||-||-||||.|||||.|||||| 308
query           240 TTGAAAGTTATTCATATCTTGAAAAACGTGCCATCGTAATCTAAAAA-AAGTAAAGTTGGCACATGT 307
```

q

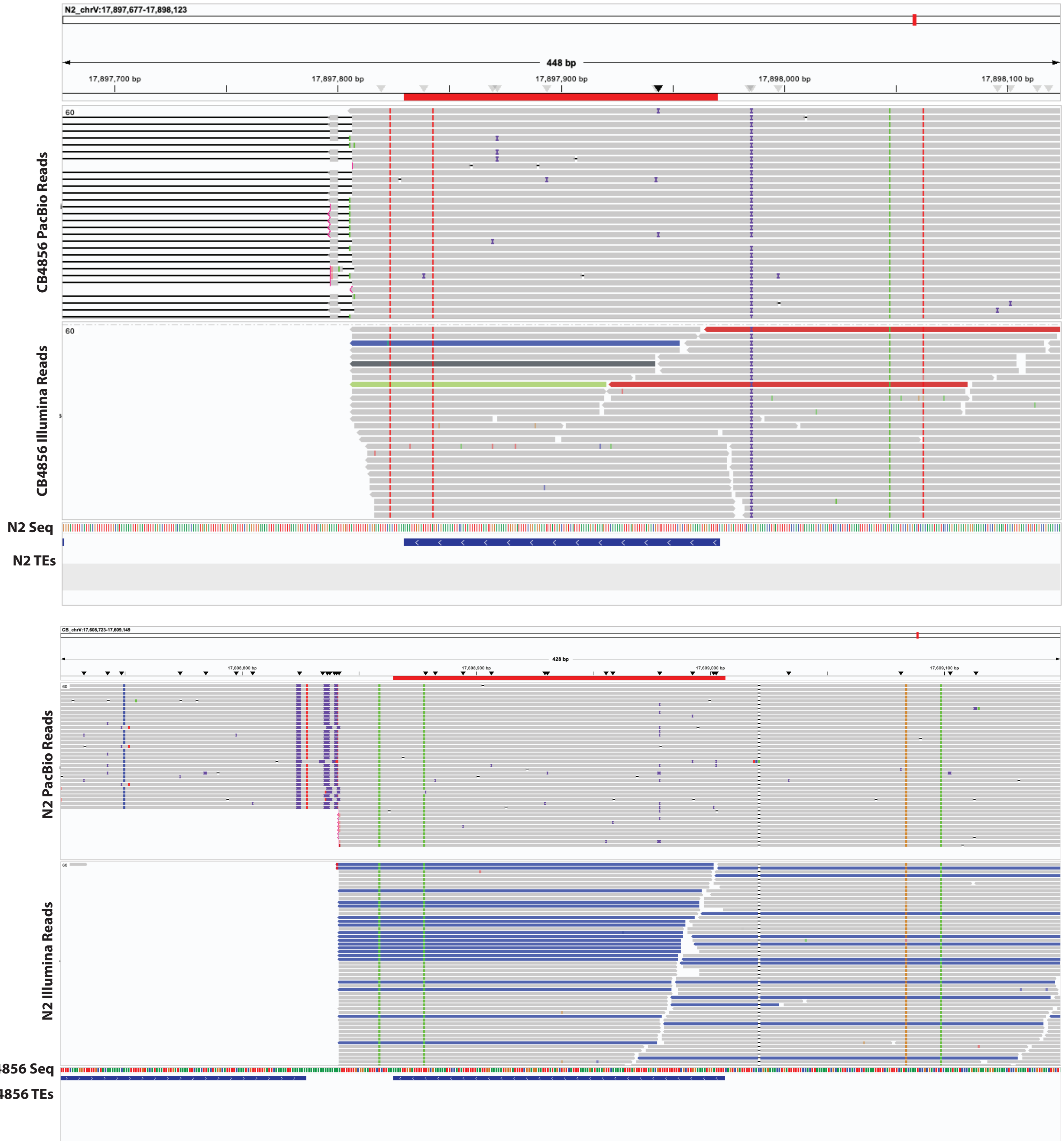


```
target            0 -AAGTTATCTTTCAGGGAAAATTGGTTATTTTTTGCGGAATTTCACTTAAAAAATTGGATTTTATGCGAAAAATTCCATATTTTTTAATTGGAAAAATGGGATTTTCGGAGGAATTTATA
                  0 -||.|||-.....|...||||......|||||||||||||||||.||||||||||||||||||.||||||||||||||||||||||||||||||||||||||||||||||||||||||||
query             0 AAAATTA-AAAAAAAAAAAAAAAAAAAATTTTTTGCGGAATTTCTCTTAAAAAATTGGATTTTTTGCGAAAAATTCCATATTTTTTAATTGGAAAAATGGGATTTTCGGAGGAATTTATA

target          119 GCTGTCATTTAAAAGATAAAGTTCAAAAATTTAGTTTTTAGACGTTTTTTTTTCGCTTGGAAAATGGATTTTCAGTCGATTTTTCA-CGAGAAACCTGATTTTTTCCACTTAAAAATCGC
                120 ||||||||||||||||||||||||||||||||||||||||||||||||||||||||||||||||||||||||||||||||||||||-|||||||||||||||||||||||||||||||||
query           119 GCTGTCATTTAAAAGATAAAGTTCAAAAATTTAGTTTTTAGACGTTTTTTTTTCGCTTGGAAAATGGATTTTCAGTCGATTTTTCACCGAGAAACCTGATTTTTTCCACTTAAAAATCGC

target          238 AAT 241
                240 ||- 243
query           239 AA- 241
```

Supplementary Figure S6. Visualization of inter- and intra-chromosomal TE movement. (a-h) Pairs of IGV visualizations and pairwise sequence alignments depicting inter-chromosomal transposition events. (i-q)Pairs of IGV visualizations and pairwise sequence alignments depicting intra-chromosomal transposition events. For all IGV visualizations, the top image shows the alignment of PacBio HiFi long reads and Illumina short reads from our CB4856 Hawaiian genome to our N2 Bristol genome as the reference sequence. All images on the bottom of each panel show the alignment of PacBio HiFi long reads and Illumina short reads from our N2 Bristol genome to our CB4856 Hawaiian genome as the reference sequence. In each IGV image, TE annotations are shown in the lowest track below the reference sequence. Transposition is visualized by: 1) differences in genomic coordinates; 2) the lack of high quality reads spanning the TE sequences and their flanks in either genome (i.e. fewer reads from the TE site in N2 Bristol should align well to the translocation region in the CB4856 Hawaiian genome if the flanking sequences are very different); 3) the alignment of clipped/fragmented reads (shown with red caps at clipped read ends) that do not span TE flanks due to lack of homology; and, 4) deletions called by IGV that span TE sequences in one genome combined with a low degree of homology when comparing TE-flanking sequences. To aid visualization of the specific TE sequences analyzed for transposition, red bars adjacent to the genomic coordinates indicate the region of interest overlapping each TE sequence. In each panel, pairwise sequence alignments are shown for the N2 Bristol TE sequence (target) and the CB4856 Hawaiian TE sequence (query), and each alignment includes the 50 bp flanking sequences upstream and downstream of each TE.
