# Supplementary figures and images for: Transposable elements and heterochromatic regions are enriched for structural variation and sequence divergence in the genome of wild-type Caenorhabditis elegans
Source: G3 (Bethesda). 2025 Apr 30;15(7):jkaf092. doi: 10.1093/g3journal/jkaf092 (PMC12239620; doi:10.1093/g3journal/jkaf092)

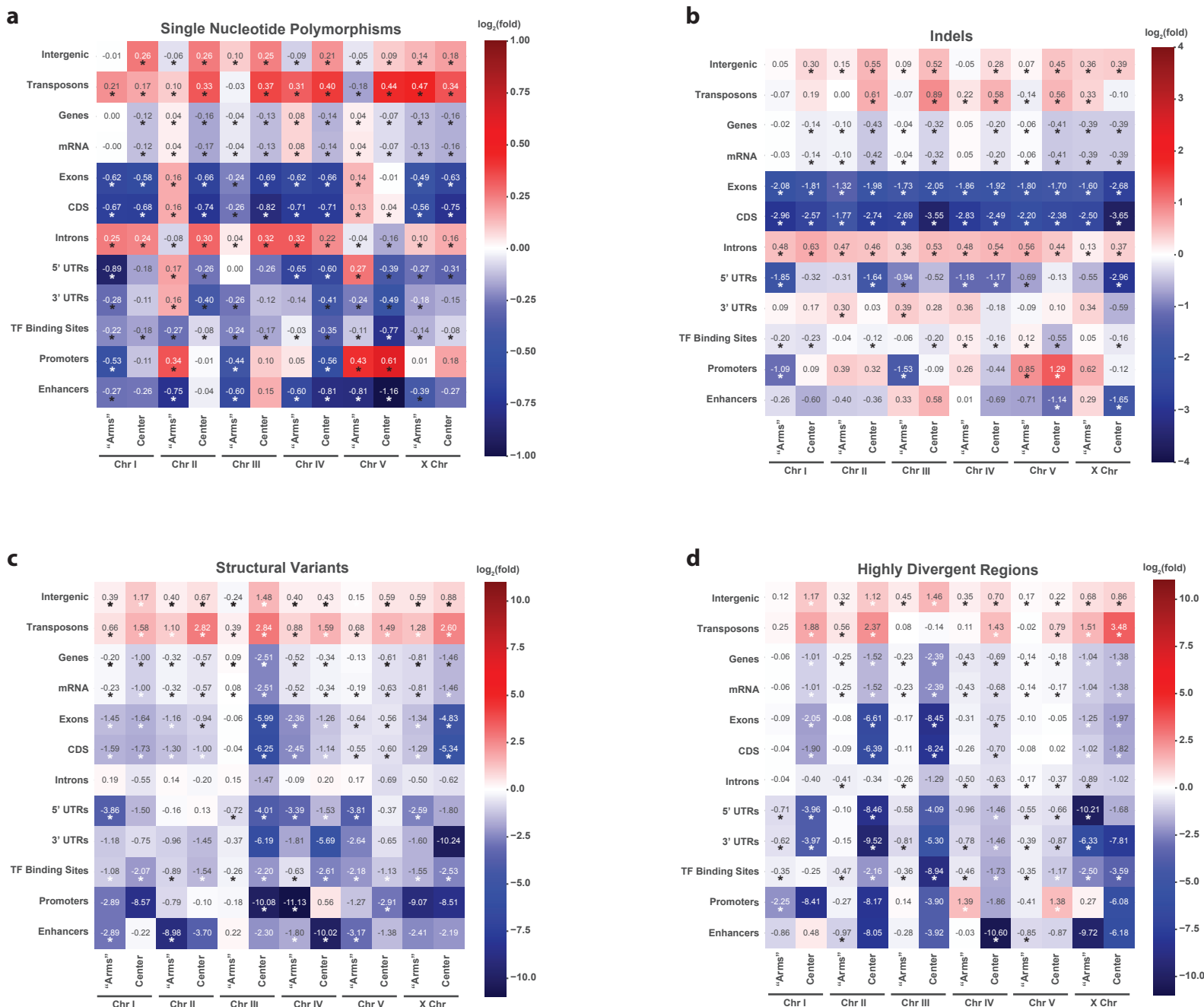

Supplement: jkaf092_Supplementary_Data [file jkaf092_supplementary_data.zip › 28833551/Supplemental_Figure_S2.pdf]
